# Mapping the evolving landscape of super-enhancers during cell differentiation

**DOI:** 10.1186/s13059-021-02485-x

**Published:** 2021-09-15

**Authors:** Yan Kai, Bin E. Li, Ming Zhu, Grace Y. Li, Fei Chen, Yingli Han, Hye Ji Cha, Stuart H. Orkin, Wenqing Cai, Jialiang Huang, Guo-Cheng Yuan

**Affiliations:** 1 Department of Pediatric Oncology, Dana-Farber Cancer Institute and Harvard Medical School, Boston, MA 02115 USA; 2 Cancer and Blood Disorders Center, Boston Children’s Hospital and Dana-Farber Cancer Institute, Harvard Medical School, Boston, MA 02115 USA; 3 State Key Laboratory of Cellular Stress Biology, Innovation Center for Cell Signaling Network, School of Life Sciences, Xiamen University, Xiamen, 361102 Fujian China; 4 Howard Hughes Medical Institute, Boston, MA 02115 USA; 5 Department of Genetics and Genomic Sciences, Charles Bronfman Institute for Precision Medicine, Icahn School of Medicine at Mount Sinai, New York, NY 10029 USA

**Keywords:** Super-enhancers, Enhancer, Differentiation, Dynamics, 3D genome, Hierarchy

## Abstract

**Background:**

Super-enhancers are clusters of enhancer elements that play critical roles in the maintenance of cell identity. Current investigations on super-enhancers are centered on the established ones in static cell types. How super-enhancers are established during cell differentiation remains obscure.

**Results:**

Here, by developing an unbiased approach to systematically analyze the evolving landscape of super-enhancers during cell differentiation in multiple lineages, we discover a general trend where super-enhancers emerge through three distinct temporal patterns: conserved, temporally hierarchical, and de novo. The three types of super-enhancers differ further in association patterns in target gene expression, functional enrichment, and 3D chromatin organization, suggesting they may represent distinct structural and functional subtypes. Furthermore, we dissect the enhancer repertoire within temporally hierarchical super-enhancers, and find enhancers that emerge at early and late stages are enriched with distinct transcription factors, suggesting that the temporal order of establishment of elements within super-enhancers may be directed by underlying DNA sequence. CRISPR-mediated deletion of individual enhancers in differentiated cells shows that both the early- and late-emerged enhancers are indispensable for target gene expression, while in undifferentiated cells early enhancers are involved in the regulation of target genes.

**Conclusions:**

In summary, our analysis highlights the heterogeneity of the super-enhancer population and provides new insights to enhancer functions within super-enhancers.

**Supplementary Information:**

The online version contains supplementary material available at 10.1186/s13059-021-02485-x.

## Background

Enhancers are distal regulatory elements critical to establishing and maintaining cell type-specific gene expression programs. Dense clusters of enhancers, known as stretch enhancers or super-enhancers (SEs), are occupied by high density of master regulators, cohesins, mediators, and coactivators and are decorated with unusually high H3K27ac signals [1–4]. SEs are thought to play an important role in the maintenance of cell identity, and disruption of their activities, such as by genetic mutation, has been linked to higher susceptibility to diseases [2, 4, 5]. Recent studies suggest that a subset of SEs contain hierarchical structure [6–10]. Within a hierarchical SE, the hub enhancer plays a critical role in orchestrating its structural and functional organization [7].

It remains poorly understood how SEs are established temporally during cell differentiation or in response to an environmental cue. Previous studies are sporadic and limited in scope [11–14]. These analyses suggest that entire SEs may be established rapidly to initiate inflammatory response [11]. A few case studies have dissected the evolving SE landscape during differentiation in a limited number of loci. For example, two studies [10, 15] examined the progressing landscape of one SE near the *Wap* locus during mammary gland’s differentiation and found that there is temporal and functional hierarchy between the elements. Despite these advances, a systematic understanding of the dynamic establishment of the SE landscape during cell differentiation is still lacking.

**Fig. 1:**
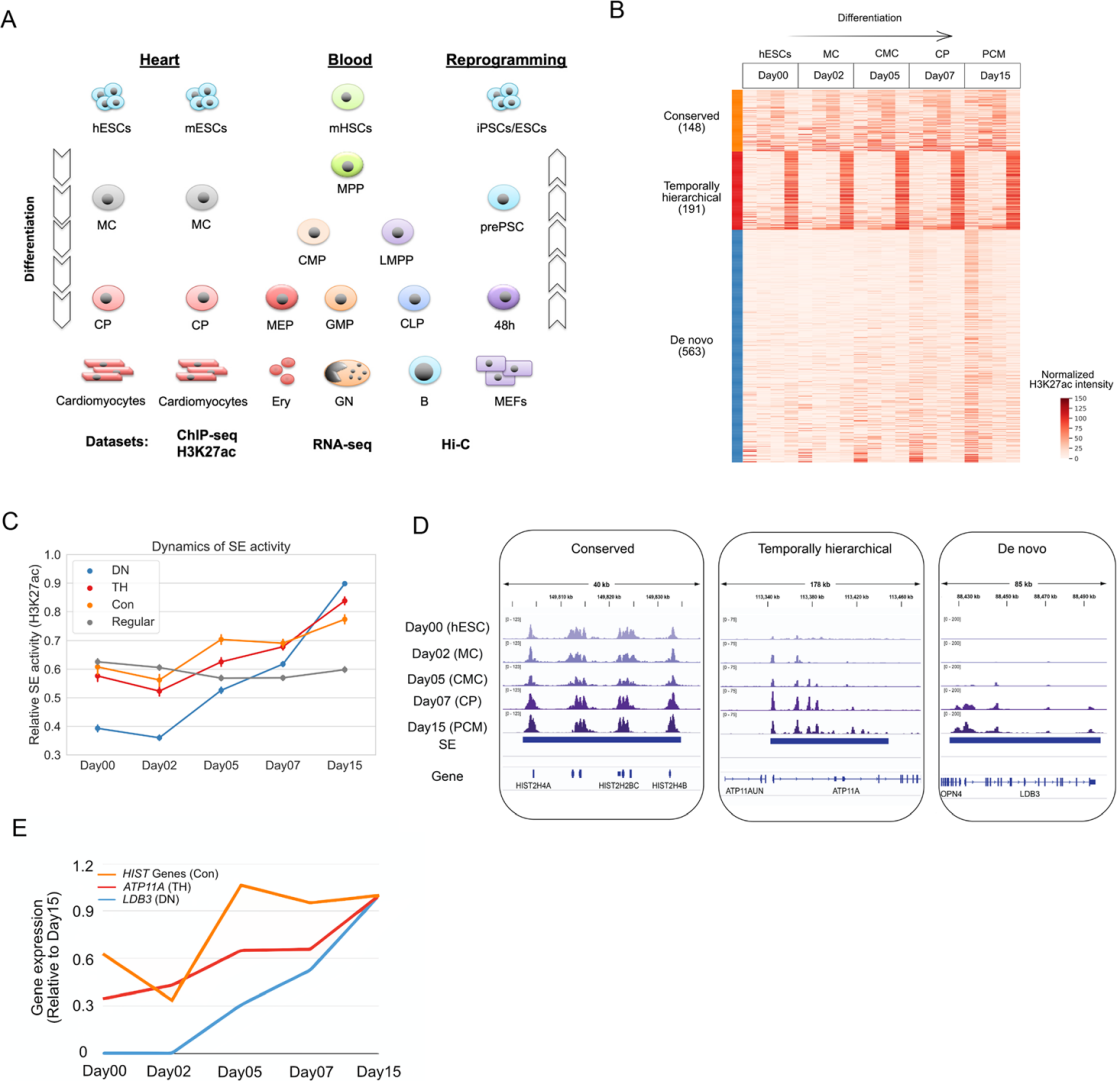
Mapping the evolving landscape of SEs reveals three distinct temporal patterns. **a** An overview of the interrogated systems in this study. **b** K-means clustering of the SE signal vectors across the five differentiation stages. Each row represents the signals of a SE during differentiation. Each SE is represented by 4 representative elements (sub-columns) generated by a linear interpolation approach (see the “Methods” section for details). **c** Average dynamic profiles of the three types of SEs during differentiation. Each SE is normalized to its maximum H3K27ac intensity during differentiation and the average profile is shown. **d** Genome browser snapshots showing typical examples for Con, TH, and DN SEs. The tracks shown are H3K27ac ChIP-seq signals. **e** Expression profile of the genes shown in **d**

To investigate the general patterns of SE establishment during cell differentiation, here we developed a systematic approach to map the evolving landscape of SEs during cell differentiation based on the dynamic pattern of chromatin states. We applied this method to several publicly available time-course chromatin immunoprecipitation followed by sequencing (ChIP-seq) data (Fig. 1a). Our analysis identified three distinct SE subtypes, each associated with a distinct temporal pattern. We integrated RNA-seq and Gene Ontology (GO) information to gain functional insights and interrogated Hi-C data to dissect the features associated with higher-order genome organization. We further applied CRISPR/Cas9 genome-editing assays to dissect the functionality of different constituent elements within temporally hierarchical SEs. We found that both the early- and late-emerged enhancers during differentiation are indispensable for target gene expression in differentiated cells. In summary, our analyses highlight the heterogeneity of the SE population and provide new insights into the dynamic establishment of SEs during cell differentiation.

## Results

### Mapping the evolving landscape of super-enhancers during differentiation reveals three distinct patterns

To map the evolving landscape of SEs in differentiated cells, we developed an unbiased clustering approach to classify SEs according to temporal histone modification patterns during cell differentiation. We focused on the H3K27ac mark, which is the most well-characterized histone mark associated with enhancer activities and often used as the basis to identify SEs [1]. To this end, we first identified constituent elements by calling H3K27ac peaks using MACS1.4 [16] and then identified SEs by using the ROSE program [1]. The temporal pattern of H3K27ac signals associated with each SE can be quantified by a numerical vector, where each element corresponds to the H3K27ac intensity of a constituent element at a time point. The dimensionality of these vectors varies from one SE to another because the number of constituent elements is not a constant (Additional file 1: Figure S1a), which leads to differential dimensions for clustering. To overcome this challenge, we standardized the vector dimension by using a linear interpolation approach (see Methods for details, Additional file 1: Figure S1b). We then applied k-means clustering (*K*=3) to divide SEs into distinct groups based on the Euclidean distance between the standardized vectors. We validated this classification approach by using various parameters and obtained similar results, suggesting that this method is robust to the choice of different parameter values (Additional file 1: Figure S1c-d, see Methods for details).

We first applied this approach to dissect the evolving landscape of SEs during human cardiomyocyte differentiation, by using a public ChIP-seq dataset [17]. In this study, the authors carried out ChIP-seq experiments to profile the dynamic changes of genome-wide H3K27ac signals during the differentiation from human embryonic stem cells (hESCs) to primitive cardiomyocytes (PCM). Our analysis identified three types of SEs with distinct temporal patterns (Fig. 1b–d): (a) conserved (Con, Fig. 1b), in which H3K27ac peaks are persistent throughout differentiation (e.g., *HIST2* gene cluster, Fig. 1d); (b) temporally hierarchical (TH, Fig. 1b), in which a SE contains at least one H3K27ac peak that is established at the early stage and other H3K27ac peaks that are gained gradually during the differentiation course (e.g., *ATP11A*, Fig. 1d); and (c) de novo (DN, Fig. 1b), in which constituent peaks gain high H3K27ac signals simultaneously at late stages (e.g., *LDB3*, Fig. 1d). On average, the H3K27ac intensities of the three types of SEs exhibit distinct temporal dynamics during differentiation (Fig. 1c), where Con and TH SEs show a relatively stable or moderate increasing profile and DN SEs show steep increases. As exemplified in Fig. 1e, the expression levels of the genes associated with the three types of SEs show a dynamic pattern that is concordant with enhancer activities. In summary, using the cardiomyocyte differentiation of hESCs as an example, we found that SEs in the differentiated cells show distinct temporal dynamics and applied a simple clustering approach to identify their temporal subtypes.

### SEs with different temporal patterns are associated with distinct properties and biological functions

**Fig. 2:**
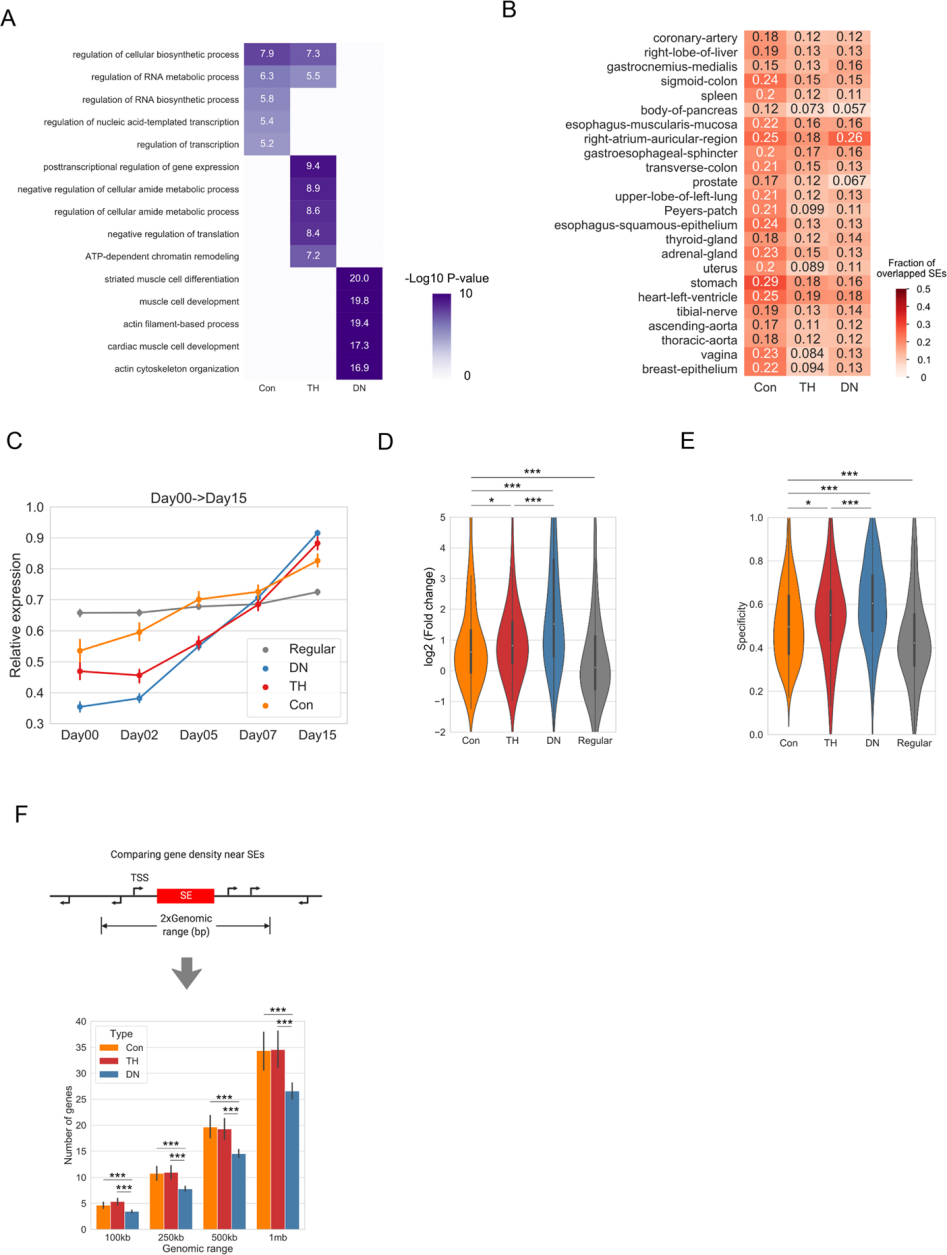
Characterizations of the three types of SEs. **a** GO enrichment pattern of the three types of SEs. Top 5 GO terms for each type of SEs are shown after removing redundant terms. **b** Heatmap showing the overlap between the three types of SEs identified in human cardiomyocytes with SEs in the other 24 tissue types. Numbers in the heatmap stand for the percentage of SEs overlapped with the 24 tissue types. **c** Line plot showing the relative expression profiles for genes associated with each type of SEs. Each gene is normalized to its maximum value during differentiation and the average is shown. **d**, **e** Comparison of the gene expression fold changes (**d**) and specificity (**e**) for the three types of SEs. Fold change is defined as the gene expression in the last stage (day 15) divided by the expression level at the first stage (day 00). Specificity measures how the genes are specifically expressed on day 15 comparing to other time points (see the “Methods” section for details). *P < 0.05, **P < 0.01, ***P < 0.001. P-values are determined by using the Mann-Whitney *U* test. **f** Comparison of gene density near the three types of SEs. *P < 0.05, **P < 0.01, ***P < 0.001. P-values are determined by using the Mann-Whitney *U* test

To investigate whether our temporal-based SE classification is functionally relevant, we performed functional enrichment analysis by using gene ontology (GO) analysis [18]. Briefly, all expressed genes within 100kb of a SE were deemed as the potential target genes. To identify the SEs’ target genes of high confidence, we further used the correlation between H3K27ac signal intensity and gene expression to filter the gene set, an approach that is also used by previous studies ([19], see the “Methods” section for details). We found that the three types of SEs exhibit distinct properties and biological functions. DN SEs are enriched in biological functions highly specific to cardiomyocytes, such as “striated muscle cell differentiation” and “cardiac muscle cell development” (*P* < 1e−15, hypergeometric test), while Con and TH SEs are enriched in more general functions (e.g. “regulation of cellular biosynthetic process” and “regulation of RNA metabolic process” for conserved SEs and “post-transcriptional regulation of gene expression” and “regulation of cellular amid metabolic process” for TH SEs) (*P* < 1e−5, hypergeometric test, Fig. 2a). Importantly, although Con and TH SEs are enriched in fewer specific functions than DN SEs, only 7~29% of the Con and TH SEs are characterized with conserved activity when comparing with SEs identified from other cell types (Fig. 2b). Furthermore, most of their associated genes (70%, 66%, and 78% for Con, TH and DN SEs, respectively, Additional file 1: Figure S2a) are not house-keeping genes [20].

We hypothesized that the temporal differences between the three types of SEs may reflect distinct regulatory roles at different developmental stages. Therefore, we compared the temporal expression patterns of their target genes and observed striking differences. On average, DN SE-associated genes undergo the most significant changes in transcriptional activities during differentiation, whereas Con and TH SE-associated genes are upregulated at a more moderate level (Fig. 2c, d). Moreover, DN SE-associated genes are more specifically expressed to the final stage (Fig. 2e). Consistent with this general trend, cardiomyocyte marker genes, such as *MYH7* and *ACTN2*, are associated with DN SEs (Additional file 1: Figure S2c-e) and their expression levels display a significant increase during differentiation (Additional file 1: Figure S2b). Of note, DN SEs tend to locate in regions with lower gene densities (Fig. 2f), suggesting that cell-type-specific functions are more likely to be regulated by long-range chromatin interactions comparing to less-specific functions.

### Characterizing the three types of SEs and their dynamics by chromatin architecture features

The changes in transcriptional regulation landscape during cell differentiation are usually coupled with dynamic 3D chromatin organization, whereas how 3D genome organization correlates with the establishment of SEs remains poorly understood [21, 22]. Therefore, we were motivated to investigate whether the evolving landscape of SEs is associated with the 3D chromatin organization dynamics. To this end, we analyzed the accompanying Hi-C data for cardiomyocyte differentiation [17] and quantified the dynamic changes of several well-characterized higher-order chromatin features in the following.

**Fig. 3:**
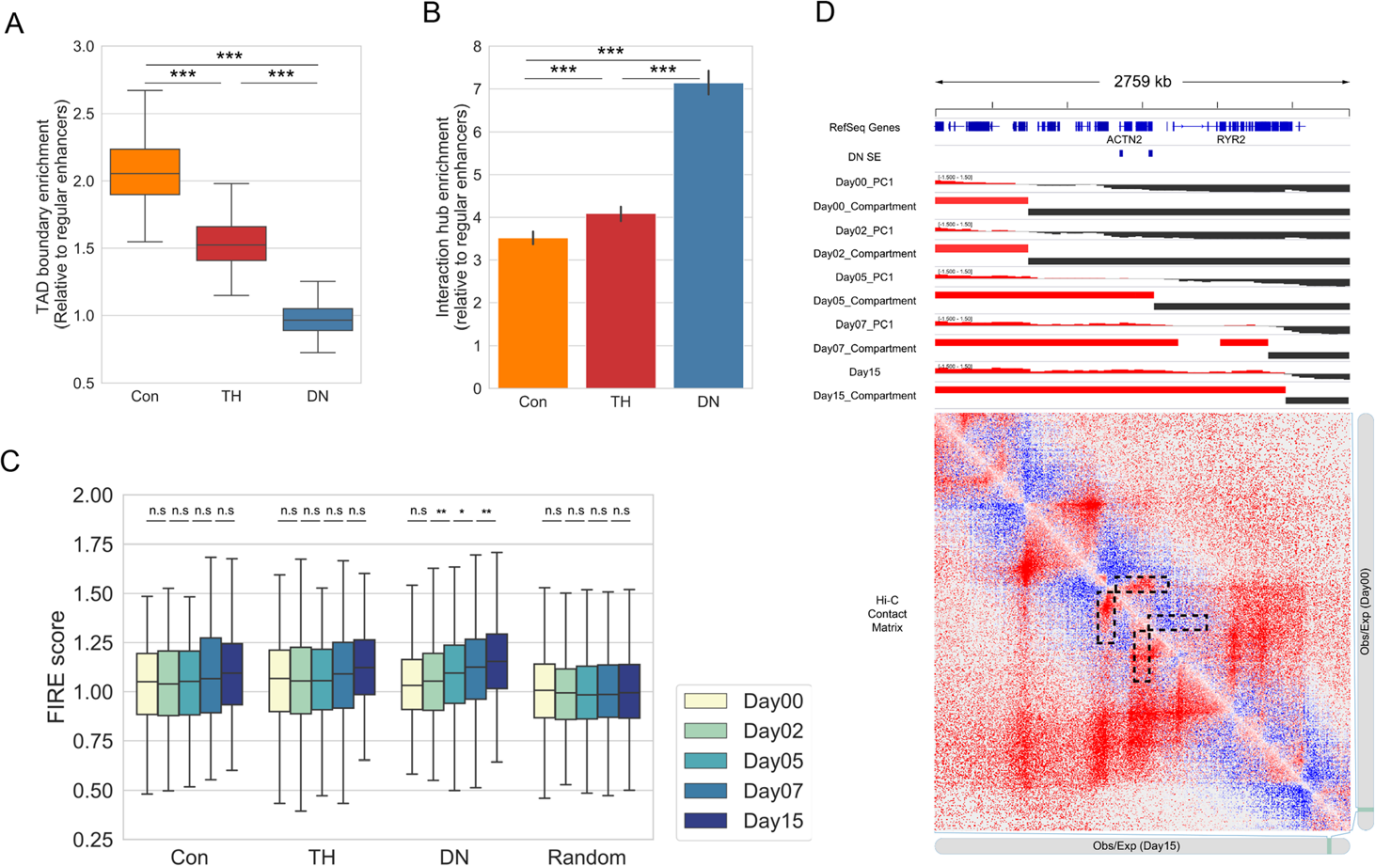
Higher-order chromatin features of the three types of SEs. **a** Box plot showing the fold enrichment of SEs at the TAD boundaries over regular enhancers. ***P < 0.001, Mann-Whitney *U* test. **b** Bar graph showing the fold enrichment of the three types of SEs on the FIRE elements in day 15 cells over random regions. ***P < 0.001, Mann-Whitney *U* test. **c** Box plot showing the FIRE scores of the three types of SEs during differentiation. n.s., not significant, *P < 0.05, **P < 0.01. P-values are determined by using the Mann-Whitney *U* test. **d** Genome browser snapshot showing two DN SEs within dynamic compartments and increased local contacts. Tracks from top to bottom, RefSeq genes, PC1 compartment signals, and compartment types from day 00 and day 15, where red represents compartment A and black represents compartment B. The contact matrix shows interaction patterns for day 00 (above diagonal) and day 15 (below diagonal), respectively. The rectangular boxes indicated by the black dashed lines highlight the increased local contacts in the SE regions

First, on the broadest scale, the chromatin is divided into two distinct types of compartments: A and B [23]. The A compartments are typically open and enriched with protein-coding genes, whereas the B compartments are closed and gene-poor [23]. During differentiation, switching from B to A compartments provides a mechanism for mediating the transcriptional levels of important developmental or lineage-specific genes [21, 24, 25]. To investigate to what extent compartment switching contributes to the activities of different types of SEs, we divided the genome into four types: stable A or B, dynamic compartments “A to B,” and “B to A” by comparing the Hi-C compartment signals between the first and last stage of differentiation. While most SEs are located in stable A compartments (Additional file 1: Figure S3a), we also observed subtle differences among the three subtypes. DN SEs show a slightly higher percentage located in dynamic compartments (“B to A” or “A to B”) than Con and TH SEs, although the differences are not statistically significant (*P*=0.11 for DN vs. TH SEs; *P*=0.17 for DN vs. Con SEs, chi-square test, Additional file 1: Figure S3a). For example, two DN SEs controlling lineage-specific genes, *ACTN2* (encoding an alpha-actinin isoform that is expressed in cardiac muscles [26]) and *RYR2* (encoding the RYR2 protein that functions as the major component of calcium channel that supplies ions to the cardiac muscle [27]), undergo dynamic compartment switching during cell differentiation (Fig. 3d).

Topologically-associating domains (TADs), defined as the genomic regions with high intra-domain interactions and few inter-domain interactions, act as structural units in genome organization and transcriptional regulation [28]. We then inspected the genomic position of the three types of SEs with respect to TAD boundaries. Using randomly sampled regular enhancers as the control, we found that Con and TH SEs are enriched at the boundaries of TADs (Fig. 3a, see the “Methods” section for details). Consistent with previous studies [29, 30], this analysis suggests that TAD boundaries are enriched in SEs with constitutive activity.

Previous studies from our and other groups have reported that enhancers with high local chromatin interactions, such as hub enhancers [7] and frequently interacting regions (FIREs) [31, 32], play a profound role in gene regulation. FIREs are chromatin regions that show significantly high enrichment on local chromatin contacts. Genes associated with SEs associated with higher FIRE scores usually have higher expressions (Additional file 1: Figure S3b). As such, we next asked whether the three types of SEs are differentially enriched in local chromatin interactions. To this end, we first identified FIREs at day15 cells by using FIREcaller [31] and tested the overlap between SEs and FIREs. DN SEs are more enriched with FIREs than Con and TH SEs, while all three types of SEs show significant enrichment in FIREs compared to random genomic regions (Fig. 3b). To check the dynamics of chromatin interaction at the three types of SEs, we then compared the FIRE scores at the three types of SEs during differentiation. As shown in Fig. 3c, DN SEs exhibit a more variable pattern in FIRE score during differentiation than Con and TH SEs. Despite the mild changes of FIRE scores between two neighboring stages, Con and TH SEs have increased spatial interactions between day 15 and day 0, suggesting that the local interactions contribute to the stage-specific gene regulation during differentiation. As exemplified in Fig. 3d, two DN SEs located in dynamic compartments are associated with a concomitant increase of the local enrichment of Hi-C interactions. Altogether, these results demonstrate that the dynamic chromatin conformation and local chromatin states work in concert to turn on lineage-specific genes.

### SE temporal classification is reproduced in other systems

We then asked whether the patterns described above were a unique phenomenon for human cardiomyocyte differentiation or generally applicable to other differentiation processes or species. To this end, we repeated this procedure to analyze several public datasets.

First, we considered a public H3K27ac ChIP-seq dataset for mouse cardiomyocyte differentiation [33], which covers four differentiation stages: embryonic stem cells (ESCs), mesoderm (MES), cardiac precursor (CP) cells, and cardiomyocytes (CM). We identified three types of SEs with similar temporal patterns, which were annotated again as Con, TH, and DN, respectively (Additional file 1: Figures S4a-b, S9a). Of note, the relative proportion of each type is similar between the two species (1:1.5:4.2 for mouse versus 1:1.4:4.4 for human), suggesting the temporal patterns of SE activities are evolutionary conserved. Like its human counterpart, we observed significant differences in the target gene expression patterns across these three types of SEs, with the target genes of DN SEs undergoing the most dynamic changes in transcriptional activities (Additional file 1: Figure S4c-e). Functional enrichment analysis also led to similar results, where DN SEs are enriched with cardiomyocytes related functions, such as “actin filament-based process” and “heart development” (*P* < 1e−10, hypergeometric test, Additional file 1: Figure S4f). Furthermore, compared to Con and TH SEs, DN SEs are associated with lower gene density, consistent with the pattern in human cardiomyocytes (Additional file 1: Figure S4g). Altogether, these results suggest that our findings are conserved between human and mouse cardiomyocytes.

**Fig. 4:**
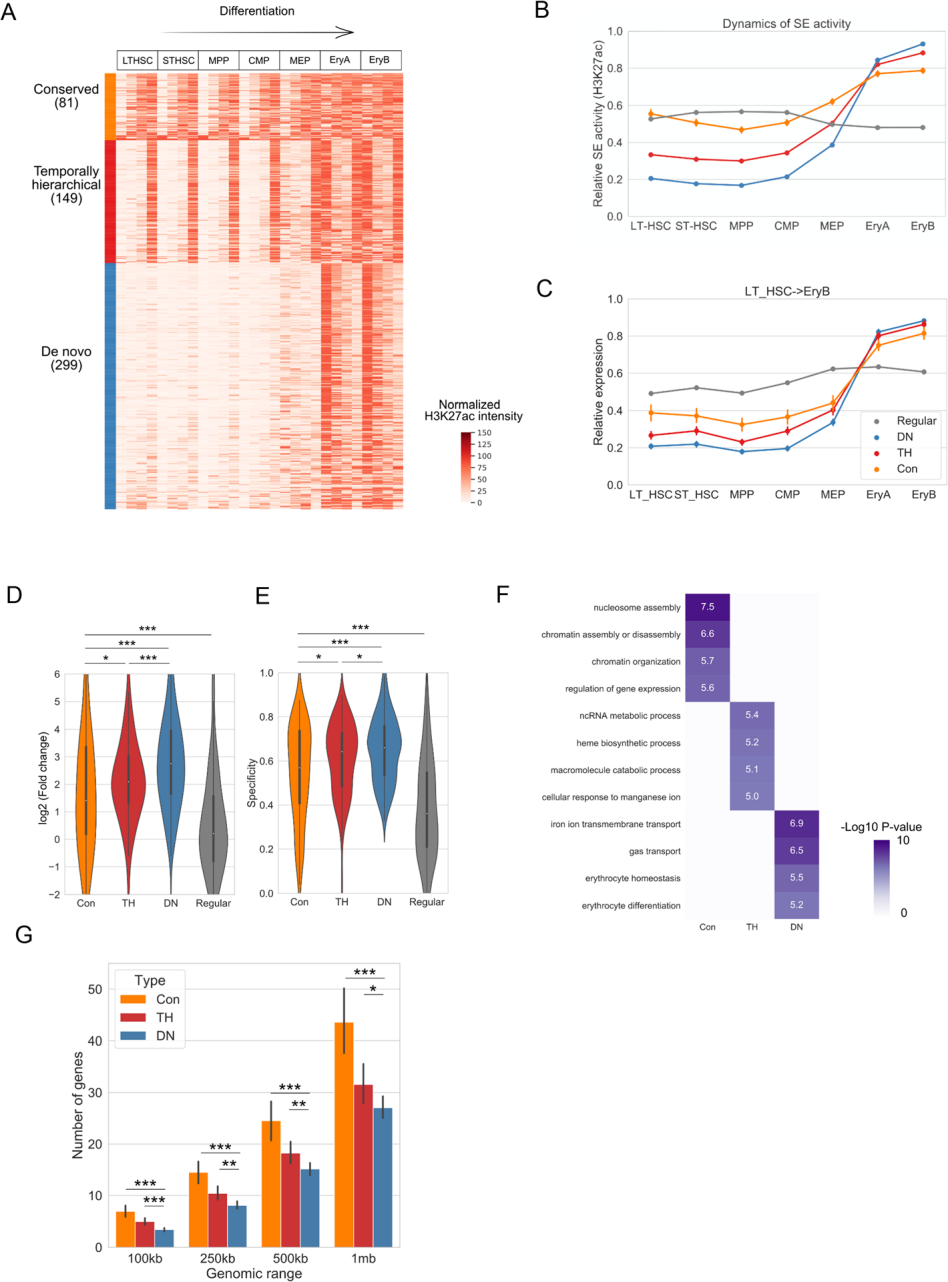
Mapping the evolving landscapes of SEs in mouse erythroid cells reveals similar patterns. **a** K-means clustering of the SE signal vectors across erythroblast differentiation. LTHSC, long-term hematopoietic stem cells; STHSC, short-term hematopoietic stem cells; MPP, multipotent progenitor cells; CMP, common myeloid progenitor; MEP, megakaryocyte erythroid progenitor; EryA and EryB, erythroblasts. **b** Average dynamic profiles of the three types of SEs during differentiation. Each SE is normalized to its maximum H3K27ac intensity during differentiation and the average profile is shown. **c** Line plot showing the relative expression profiles for genes associated with each type of SEs. Each gene is normalized to its maximum value during differentiation and the average profile is shown. **d**, **e** Comparison of the gene expression fold changes (**d**) and specificity (**e**) during differentiation for the three type of SEs. *,*P* < 0.05, **,*P* < 0.01, ***,*P* < 0.001. P-values are determined by using the Mann-Whitney *U* test. **f** GO enrichment pattern of the three types of SEs. **g** Comparison of gene density near the three types of SEs. *P < 0.05, **P < 0.01, ***P < 0.001. P-values are determined by using the Mann-Whitney *U* test

Next, we tested whether the observed patterns can be generalized to other lineages. To this end, we analyzed the data from Astiaso et al. [34], which profiled the chromatin state dynamics across multiple cell types of hematopoietic differentiation for mouse in vivo. We dissected the evolving landscape of SEs in three cell types: erythroblasts, B cells, and granulocytes (GN), respectively, and found that the SEs show similar patterns. For instance, during erythropoiesis, unbiased clustering of SEs revealed three temporal patterns: Con, TH, and DN (Fig. 4a, b, Additional file 1: Figure S9b). The three types of SEs demonstrate the same hierarchy in terms of the potential in up-regulating the expression of target genes: genes associated with DN SEs are characterized with the highest fold changes during differentiation while those associated with Con SEs show relatively stable expression (Fig. 4c–e). In concordance with the higher specificity levels of genes associated with DN SEs (Fig. 4e), GO enrichment shows that DN SEs are highly enriched in biological processes that are specific to erythroid cells, such as “erythrocyte homeostasis” and “erythrocyte differentiation” (*P* <1e−5, hypergeometric test, Fig. 4f). In parallel, DN SEs are more likely to locate in less gene-dense regions than Con and TH SEs (Fig. 4g). Consistently, the same results were obtained for both B and GN cell differentiation (Additional file 1: Figures S5 and 6). Collectively, these results suggest that the three types of evolving landscape for SEs can be generalized to the development of blood lineages.

In addition to normal differentiation, cell reprogramming is induced by forced expression of master regulators and accompanied by the re-establishment of cell-type-specific chromatin states and gene expression programs. We next asked whether the identified temporal patterns could also be applied to this TF-directed reprogramming process. To this end, we analyzed the data from Chronis et al. [35], in which mouse embryonic fibroblasts (MEF) were reprogrammed to pluripotency induced by the Yamanaka factors (Oct4, Sox2, Klf4, and cMyc). We observed similar patterns except that the endpoint now is the pluripotent state (Additional file 1: Figures S7a-e, S9e). Consistent with this, the functional enrichment analysis shows that DN SEs are more enriched in terms specific to ESCs, such as “multicellular organism development” and “embryo development” (*P* < 1e−5, hypergeometric test, Additional file 1: Figure S7f). The three types of SEs also exhibit differences in terms of their preferences in genomic locations, i.e., DN SEs are more likely to be located in less gene-dense regions than Con and TH SEs (Additional file 1: Figure S7g). Collectively, these results provide strong evidence that the temporal hierarchy of SE organization is generally applicable to the differentiation of diverse cell lineages and species.

### Decommission of SEs follows reversed temporal dynamics

Cell differentiation is a highly dynamic process that not only requires the activation of a set of genes and enhancers specific to terminal linages, but also necessitates the decommission of genes and enhancers controlling pluripotency. Therefore, how SEs in the pluripotent cells are lost during differentiation is an intriguing question. To this end, we classified the temporal pattern of SE decommission.

**Fig. 5:**
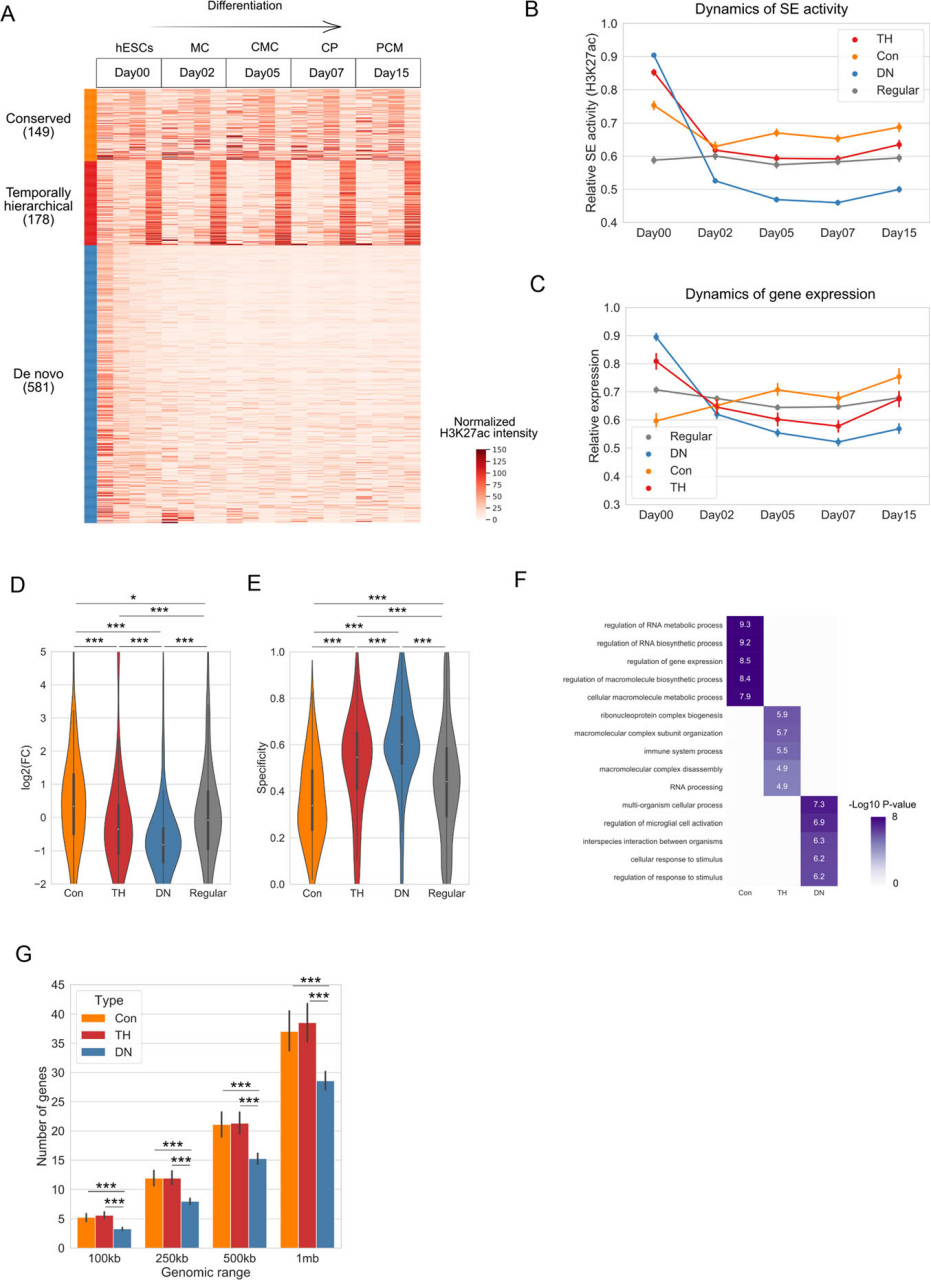
Decommission of SEs in early stages follows a similar pattern as in SE activation. **a** Heatmap showing the three types of SEs defined by progressing landscape during differentiation. **b** Average dynamic profiles of the three types of SEs during differentiation. Each SE is normalized to its maximum H3K27ac intensity during differentiation and the average profile is shown. **c** Line plot showing the relative expression profiles for genes associated with each type of SEs. Each gene is normalized to its maximum value during differentiation and the average profile is shown. **d**, **e** Violin plots showing the fold changes (day 15/day 00) (**d**) and specificity (**e**) of genes expression associated with the three types of SEs. **f** GO analysis showing the enriched functions for the three types of SEs. **g** Comparison of gene density near the three types of SEs. *P < 0.05, **P < 0.01, ***P < 0.001. P-values are determined by using the Mann-Whitney *U* test

We first demonstrated the characterization of SE decommissions in the human cardiomyocyte differentiation system. We identified SEs in ESCs and repeated the same procedure to classify them into different groups based on the dynamics during differentiation (Fig. 5a, Additional file 1: Figure S9f). Consistent with the SE activation analysis, the three patterns were also observed for SE decommission: conserved (Con), temporally hierarchical (TH), and de novo (DN), but in reverse temporal order. In contrast to Con SEs whose signals are relatively stable, DN SEs display a rapid loss of H3K27ac signals during differentiation and TH SEs demonstrate a gradual loss of H3K27ac signals (Fig. 5b). Expression levels of the genes associated with these SEs (Fig. 5c) show similar dynamics to the SE activation process. Furthermore, significant differences in the target gene expression patterns were observed for the genes associated with the three types of decommissioned SEs, with the target genes of DN SEs undergoing the most significant decrease in expression (Fig. 5c–e). GO enrichment analysis reveals that the DN SEs, which were decommissioned fast, are enriched in biological functions that are specific to ESCs, such as “multi-organism cellular process” (*P* < 1e–6) (Fig. 5f). Moreover, DN SEs in the SE decommission process are more likely to locate in less gene-dense regions than Con and TH SEs (Fig. 5g). Consistent with the results in human cardiomyocytes, the same results for the decommissioned SEs during differentiation of the erythroid cells were also obtained (Additional file 1: Figure S8, Additional file 1: Figure S9g). In summary, our results suggest that SE decommission follows reversed temporal dynamics to the SE activation.

### Genome editing analysis within TH SEs reveals that both early- and late-emerged enhancers are indispensable in differentiated cells

To gain mechanistic insights into the role of temporal dynamics of SE activities during cell differentiation, we tested the effects of perturbing specific constituent elements that are activated at different stages within TH SEs on gene expression. We focused on the well-characterized in vivo mouse erythropoiesis system due to the availability for genetic manipulation. The genomic locations of the SEs and corresponding types were identified by our computational analysis described above (Fig. 4). We focused on the TH SEs because they contain enhancer elements that come into commission at different stages.

**Fig. 6:**
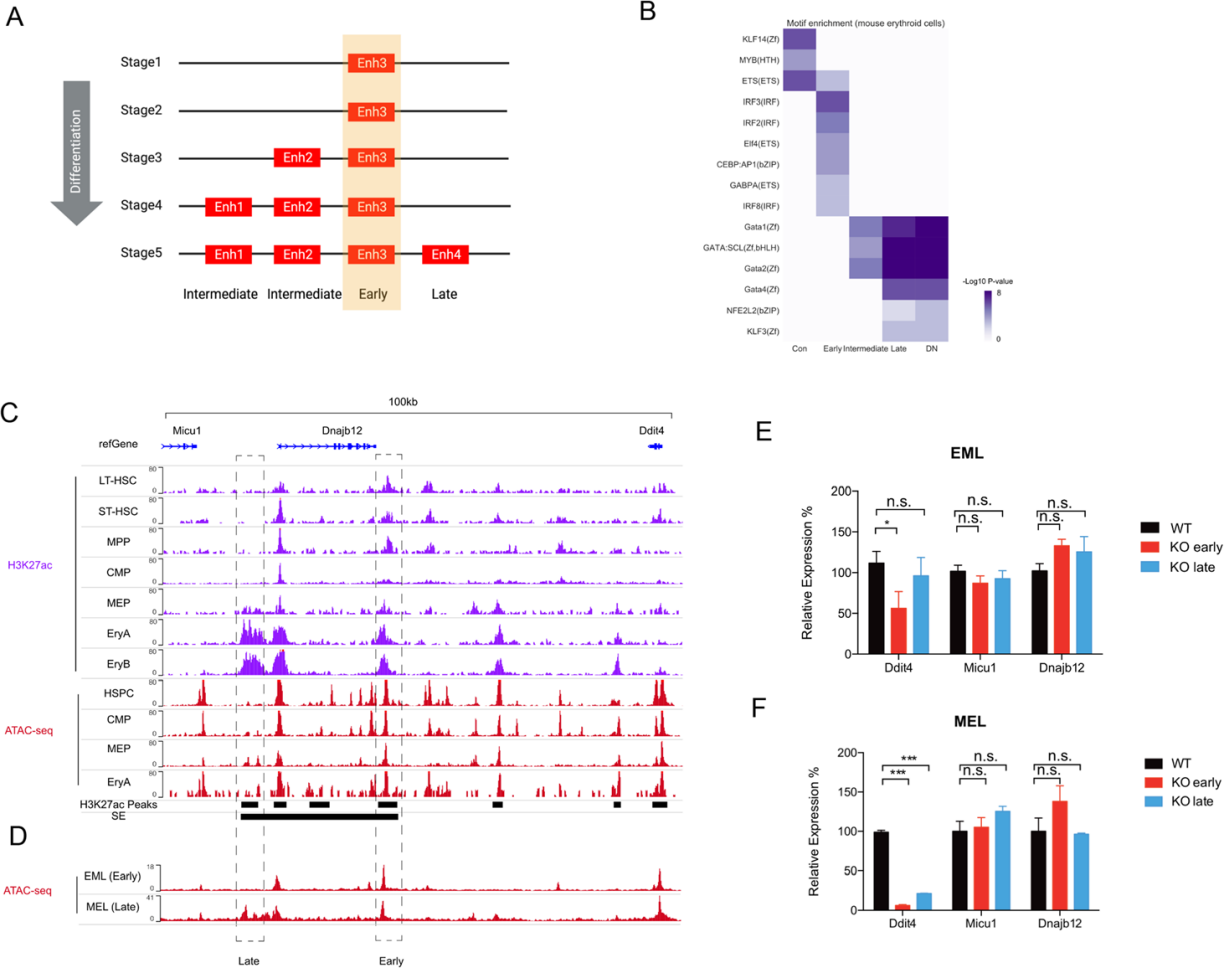
Dissection of the constituent elements within TH SEs. **a** An illustration of the early, intermediate, and late elements within TH SEs. **b** Heatmap showing the differential motif enrichment pattern for the three types of elements within TH SEs as well as the elements in Con and DN SEs. Color represents the –log10 (*P* value). **c** Genome browser snapshot showing the progressing landscape of the *Dnajb12* SE locus during erythroid cell differentiation. Early and late enhancers are defined according to the temporal order of emergence. **d** ATAC-seq data showing chromatin accessibility landscape of the SE locus in EML and MEL cells. EML and MEL cells represent the hematopoietic pluripotent cells and differentiated erythroid cells, respectively. The early enhancer is activated in EML and MEL cells, while the late enhancer is activated in MEL cells. **e** Comparison of gene expressions between wild type (WT) and enhancer-knockout EML cells. **P < 0.01; n.s., not significant. . P-values are determined by using the Student’s *t* test. **f** Comparison of gene expressions between wild type (WT) and enhancer-knockout MEL cells. **, *P* < 0.01; n.s., not significant.  P-values are determined by using the Student’s *t* test

Based on the temporal order of enhancers’ commission, we classified the enhancers within each SE into three types: early, intermediate, and late, as illustrated in Fig. 6a. Motif enrichment analysis reveals that early and late elements are enriched in distinct sets of TFs. For mouse erythroblasts, the late enhancers show a similar enrichment pattern with enhancers in DN SEs and are enriched in TFs with well-known functions specific to the erythroid lineage, such as Gata1 and GATA:SCL, while early enhancers are enriched with factors that are characterized with relatively general functions (e.g., ETS [36], Fig. 6b), suggesting the proper control of gene expression requires the coordinated activity of multiple factors in a temporally ordered manner. Furthermore, to test if there are factors that bind to the different enhancers in a quantitatively different way that may be omitted by motif analysis, we used Binding Analysis for Regulation of Transcription (BART) [37]. BART is a bioinformatics tool to infer functional transcription factor binding by leveraging thousands of real ChIP-seq datasets from the public domain for regulator prediction. Consistent with the motif analysis, the master regulators for erythropoiesis, such as GATA1, TAL1, and LDB1, were found to be more enriched in late enhancers than early enhancers or conserved enhancers (Additional file 1: Figure S10f). Interestingly, the architectural proteins CTCF and RAD21 also show an increasing enrichment in late enhancers (Additional file 1: Figure S10f), likely suggesting that late enhancers are more involved in regulation related to higher-order chromatin structure. Similarly, the differences in enriched motifs are also consistent in other lineages as well as the reprogrammed ESCs [38] (Additional file 1: Figure S10a-e). Taken together, the enrichment of distinct sets of motifs for early and late elements within TH SEs suggests that the temporal order of the establishment of elements is directed by the binding of transcription factors.

To rigorously evaluate the function of different constituent elements within TH SEs during development, we performed CRISPR-Cas9-mediated genome editing in two complementary in vitro systems: mouse erythroid-myeloid-lymphoid (EML) cells and mouse erythroleukemia (MEL) cells. EML cells, representing multipotent hematopoietic precursor cells, provide an ideal validation system in hematopoietic cells at an early stage [39], while MEL cells, immortalized erythroid cells derived from mouse adult spleens, provide a model system for mature erythroid cells at a late stage during hematopoietic differentiation [39, 40]. We focused on the *Ddit4* locus (chr10:59,864,542-59,952,997), where the early enhancer in the locus was persistent during differentiation, whereas the late enhancer is erythroid-specific as H3K27ac peaks emerged at the MEP stage and are well established later in erythroblasts (Fig. 6c, d). For each system, we applied paired guide RNAs (sgRNAs) targeting 5′ and 3′ flanking regions of selected enhancers to achieve balletic editing of both enhancers respectively. The deletion size of the enhancers was refined to 1–1.5kb, including GATA1 motifs in the late enhancer and ETS motifs in the early enhancer. We then examined the expression levels of all nearby genes by qPCR experiments upon enhancer deletion in EML cells and MEL cells, respectively. In EML cells, *Ddit4* transcripts were reduced after the KO of the early enhancer but not the late enhancer, suggesting that the early enhancer is important for regulating the target gene expression (Fig. 6e). In contrast, *Ddit4* expression was remarkably reduced in the absence of either late or early enhancers in mature erythroid MEL cells, suggesting both early and late enhancers are required for gene expression at the late stage (Fig. 6f). Transcripts of *Micu1* and *Dnajb12* in the same locus remained unaffected upon enhancer deletion in either EML or MEL cells (Fig. 6e–f). Altogether, these results suggested that both enhancers that emerged at early and late stages are required for maintaining the proper gene expression during development, whereas late enhancers are only required for gene expression in differentiated cells.

## Discussion

To dissect the evolving landscape of SEs, here we developed an unbiased computational method to characterize the temporal hierarchy of SE activity based on time-course H3K27ac ChIP-seq data during cell differentiation. We identified three types of SEs with distinct temporal patterns, which were annotated as Con, TH, and DN, respectively. The target genes of these SEs differ in biological functions as well as transcriptional responses. In particular, the target genes of DN SEs are enriched with cell-type-specific functions and exhibit strong dynamic changes of transcriptional activities. DN SEs are also associated with significant dynamic changes of 3D chromatin organizations.

SEs have been well characterized in many cell types [41–43]; however, the temporal characterization of SEs has been limited in few systems or loci. We have analyzed the genome-wide temporal establishment of SEs in multiple biological systems, including cardio-genesis, hematopoiesis, and somatic reprogramming, and across human and mouse lineages. Observations from those biological systems are highly consistent, suggesting the temporal patterns, Con, TH, and DN, are general features of SE formation and are conserved in human and mouse differentiation.

TH SEs contain a set of temporally heterogeneous regulatory elements. Those elements come into commission at different time points during differentiation and are enriched in distinct sets of TFs. Specifically, the early ones are enriched in ETS factors and late ones enriched in lineage-specific factors, such as GATA1 in erythroid cells and MEF2C in cardiomyocytes, suggesting that these elements may differentially sense the external stimuli or signaling during differentiation. To dissect whether the temporal hierarchy between these enhancer elements correlates with functional hierarchy, we performed CRISPR-Cas9-based genome perturbation experiments for enhancers that emerged at early and late stages individually and found that both the early and late enhancers are indispensable to maintain the high expression of target genes in differentiated cells, whereas only early enhancers are required to maintain the expression of target genes in undifferentiated cells. Our finding is consistent with a recent study [44] that dissects the temporal and functional contributions of individual elements in the *Fgf5* enhancer cluster during exit from naïve pluripotency. These pieces of evidence suggest the proper control of some genes requires the coordinated activity of multiple factors in a temporally ordered manner. Another intriguing question is whether the formation of late enhancers is dependent on the early elements. Previous studies on this aspect have led to inconsistent findings. In some cases, such as the *Fgf5* enhancer cluster [44], the activities of late enhancers are not affected by the deletion of the early enhancer, whereas in other cases, such as the *Wap* enhancer cluster [10, 15], the early enhancer is required to activate the late enhancers. As such, the dependency among different constituent elements is locus-dependent. The computational approach developed in this paper will provide a valuable guide for future mechanistic investigations.

## Methods

### H3K27ac peak calling and unification of peaks

MACS1.4 [16] was used to call peaks for H3K27ac, with *P*=1e–5 as the statistical cutoff. Redundant reads were removed before peak calling. Peaks overlapped with the mm10 or hg19 blacklist ([45], https://github.com/Boyle-Lab/Blacklist/tree/master/lists) were filtered out using “bedtools intersect -v” [46].

To study enhancer dynamics across differentiation stages, we first created a peak set with unified genomic coordinates. To do this, we pooled peaks from each stage into one file and merged the overlapped peaks using “bedtools merge -d 0,” which resulted in a unified set of peaks without overlapping. Next, we compared the peaks from each individual stage with the unified peak set, and any peaks showing overlap (≥1bp) with the peaks in the unified set were added to the stage-specific peak set using the coordinates of the unified peak. This stage-specific peak set with the coordinates of the unified peaks was used for calling SEs later.

We quantified the H3K27ac signals based on the unified peak set. Briefly, we calculated the reads per million mapped reads (RPM) for each peak at each time point, which gave a peak by time point matrix. The peak by time point matrice for all the interrogated systems were summarized in Additional file 5: Table S4. To mitigate the effects of differential signal-to-noise ratios among the ChIP-seq data, we then applied quantile normalization to normalize the signals across time points. The normalized matrix was used for the following clustering analysis.

### Identification of SEs and target genes

SEs were identified using the ROSE algorithm [1]. Bam files of the H3K27ac ChIP-seq data after removing redundant reads and the stage-specific peak set with unified coordinates were used as input. Other parameters were set to default. We noticed that some SEs contain a high percentage of promoters; therefore, we further filtered out the SEs whose promoter peak percentage is higher than or equal to 50%. A promoter peak is defined as a peak that is fully contained by the TSS+/- 2kb region. By applying this criterion, a SE containing 3 or 4 peaks in total is allowed to have at most one promoter peak.

To identify the targeted genes of SEs, we took a two-step approach. First, all the expressed genes (RPKM ≥2) within 100kb of a SE were selected as potential targets. Second, to improve the accuracy of target gene identification, we further calculated the Pearson correlation coefficient between the gene expressions and SEs’ H3K27ac signals across differentiation stages, and only genes showing high correlations (Pearson correlation > 0.75) were identified as the target genes.

### Classification of the temporal pattern of SEs 

To compare the temporal patterns of SEs , we restricted the H3K27ac intensity matrix constructed as above to each SE, and then carried out a series of transformation steps to facilitate comparison. First, we sorted the columns according to the temporal variation of H3K27ac signals, which is quantified by the relative standard deviation (RSD) (defined as the standard deviation divided by the mean). Here, we used RSD to sort the enhancers to enable that the enhancers with similar variation/dynamics are grouped together. Since the dimension of each submatrix is different (due to the variation of the numbers of constituent elements within a SE), we further standardized the number of columns to 4. Specifically, we inferred the 0th, 33.3th, 66.6th, and 100th percentiles of H3K27ac signal for each time point, termed as the four representative enhancers, and then constructed a new submatrix based on values corresponding to these percentiles. We then employed K-means clustering (*K*=3) from scikit-learn [47] to cluster the SEs based on the four representative enhancers at different time points. 100 rounds of K-means clustering using randomly picked seed numbers were performed, and consensus clustering ([48], https://github.com/GGiecold/Cluster_Ensembles) was applied to generate a stable clustering result.

In our method, there are two parameters that could possibly impact the classification of SEs: the number of representative enhancers and the choice of *K* in K-means clustering. To show that our method is robust to the choice of the number of representative enhancers in the clustering step, we compared the clustering result using 4 enhancers with the results using 5 or 6 enhancers. We found that the results are similar (Additional file 1: Figure S1c). Moreover, we found that as the number of cluster K increases, the overall pattern shown in different clusters are highly similar (Additional file 1: Figure S1d), despite that the increased number of *K* used for clustering can reveal the SE dynamics in finer resolution. Therefore, our approach is robust to the choice of parameters in revealing SEs’ temporal patterns.

### Gene expression specificity analysis

Gene expression matrices were downloaded from GEO for each dataset involved in this study (Additional file 2: Table S1). We adopted the gene expression specificity measure, a metric developed by Xiao et al. [49] for the tissue-specific gene expression database TiSGeD, to quantify the specificity of a gene to a time point. Specifically, the expression specificity of a gene G at time point M is defined as $\sqrt{\frac{X_M^2}{\sum \limits_{all\  time\ points}{X}_i^2}}$, where *X*_*M*_ is the expression level of G at time point M and *X*_*i*_ is the expression at time point *i*.

### House-keeping gene analysis

House-keeping genes were downloaded from [20] and were compared with the genes associated with the three types of SEs.

### Hi-C data processing and compartment identification

Raw Hi-C data in human cardiomyocytes were downloaded from GEO with accession number GSE116862 [17]. SRA files were dumped to fastq format and then were aligned to the hg19 reference genome using HiC-Pro version 2.11.1 [50], with the default configuration parameters. A/B compartments were then identified at 50kb resolution by using the “runHiCpca.pl” script from Homer [51]. For visualization, “.hic” files were downloaded from GSE116862 and visualized using Juicebox [52].

### Identification of TAD boundaries and enrichment of SEs at boundaries

We used the insulation score approach by Crane et al. ([53], https://github.com/dekkerlab/crane-nature-2015) to identify TADs based on the normalized contact matrices at 40 kb resolution, with 600 kb and 200 kb being used as the insulation square and delta size respectively. Bins with insulation scores higher than 0.5 (≥0.5) were identified as TAD boundaries. In total, there are 3164 TAD boundaries for the day 15 sample. To test the enrichment of SE in TAD boundaries, we calculated the percentage of SE constituent enhancers overlapping with TAD boundaries for each type of SEs and compared them to the percentage of 1000 regular enhancers randomly sampled from the H3K27ac peaks. The sampling of 1000 regular enhancers was performed 20 times, and the ratio between the SE constituent enhancers and regular enhancers was calculated for plotting.

### Calculation of FIRE score and FIRE identification

We used FIREcaller [31] to detect frequently interacting regions from Hi-C data at 50kb resolution. Briefly, the raw Hi-C contact matrix was used as input and the total number of local interactions (<200kb) for each genomic locus was calculated as the raw FIRE score. FIRE scores were then normalized within and across samples by FIREcaller. Within each time point, the regions with the top 10% FIRE scores were identified as FIREs.

### Motif enrichment analysis

The “findMotifsGenome.pl” script from the HOMER package [51] was used to search for enriched motifs. The whole genome was used as the background. Factors with *P* values lower than 1e−2 were considered as enriched. Redundant enriched motifs were merged and represented by the one with the most significant *P* value. Enriched factors were further filtered out if the expression level of the corresponding gene is lower than 2 RPKM value throughout the differentiation.

### Cell culture, RNA extraction, and qRT-PCR

EML cells were cultured in IMDM media supplemented with 20% fetal bovine serum, 2 mM l-glutamine, 1.5 g/L sodium bicarbonate, and 100 ng/ml mouse SCF. MEL cells were cultured in a low-glucose DMEM supplemented with 10% fetal bovine serum, 2 mM l-glutamine, and Penicillin and Streptomycin. 2% DMSO in culture medium was used to induce MEL cell differentiation. Medium was changed every other day.

The total RNA was extracted with TRIzol (Thermo Fisher) and reverse transcribed to cDNA with QuantiTect Reverse Transcription Kit (Qiagen) according to the manufacturer’s instructions. cDNA samples were subjected to qRT-PCR using the iQ SYBR Green Supermix (Bio-Rad) in the CFX384 Touch Real-Time PCR Detection System (Bio-Rad). Primer sequences are listed in Additional file 4: Table S3. Values are expressed as log_10_2^DeltaCt using *Actin beta* (*Actb*) *or Gapdh* as a control gene.

Each transcript analysis experiment was repeated at least twice using a minimum of three biological replicates per condition. Statistical analysis was performed with an unpaired Student’s *t* test. Error bars indicate the S.E.M.; *n*=3 and 5 in Fig. 6d, e respectively. *P* values were calculated and statistical significance indicates *P < 0.05, **P < 0.01, and ***P < 0.001.

### CRISPR-Cas9-mediated perturbation of enhancers

To generate biallelic deletion of the late enhancer in SE loci, sgRNAs targeting 5′- and 3′-flanking regions of the targeted enhancers were designed and synthesized respectively. sgRNA sequences are listed in Additional file 3: Table S2. Two overlapping oligonucleotides carrying sgRNA sequence targeting 5′-flanking region and two overlapping oligonucleotides carrying sgRNA targeting 3′-flanking region were annealed and cloned, respectively, as previously described [54]. In brief, 10-uM guide sequence oligos and 10-uM complement oligo were mixed with 1X ligation buffer supplemented with 5 U of T4 polynucleotide kinase (PNK) in 10 ul reaction. Anneal in a thermocycler using 37^o^C for 30 min; 95^o^C for 5 min and then ramp down to 25^o^C at 0.1^o^C/s. The annealed oligos were then ligated into pKLV-U6gRNA(BbsI)-PGK-BFP (Addgene #50946) vector using a Golden Gate assembly. Ligation mixture [100 ng vector, 1uM annealed oligos, 40 U BbsI restriction enzyme (NEB), 1 mM ATP, 0.1 mg/ml BSA and 750 U T4 DNA ligase (NEB), and 1X restriction enzyme buffer] were incubated in a thermocycler using 20 cycles of 37^o^C for 5 min, 20^o^C for 5 min; followed by 80^o^C for 20 min.

pKLV-U6gRNA(BbsI)-PGK-BFP construct with sgRNA targeting 5′-flanking region and pKLV-U6gRNA(BbsI)-PGK-BFP construct with sgRNA targeting 3′-flanking region were co-transfected with pCas9-GFP (Addgene #44719) at the ratio of 1:1:2 into MEL cells by Lipofectamine 2000 (Invitrogen). The top 5% of GFP^+^/BFP^+^ cells 48 h post-transfection were isolated by FACS. Single cell-derived colonies were screened for bi-allelic deletion of the targeting region.

A similar strategy with sgRNAs targeting flanking regions of selected enhancers has been applied to generate biallelic deletion of the late enhancer in *Ddit4* locus, and biallelic deletion of the early enhancer in *Ddit4* locus. sgRNA sequences and genotyping primer sequences are listed in Additional file 4: Table S3. Similar strategies were used to generate biallelic deletion of enhancers in EML cells with the exception that both Cas9 and sgRNA were stably expressed using lentivirus.

## Supplementary Information

**Additional file 1.** Supplementary figures (Figure S1 – Figure S10).

**Additional file 2: Table S1.** Summary of used datasets in the study.

**Additional file 3: Table S2.** List of target enhancers, guide RNAs sequences, and genotyping PCR primers.

**Additional file 4: Table S3.** List of qRT-PCR primers used for experimental validation.

**Additional file 5: Table S4.** Summary of the evolving signals of enhancers during differentiation for each interrogated system.

**Additional file 6.** Review History.

## Data Availability

The data of the human cardiomyocyte differentiation system were downloaded from the study by Zhang et al. [17] with accession number GSE116862 [55]. Mouse cardiomyocyte data were downloaded from Wamstad et al. [33] with accession number GSE47950 [56]. The data of the mouse erythroid, B and granulocytes were downloaded from Lara-Astiaso et al. with accession number GSE60103 [57]. The reprogramming data in mouse were downloaded from Constantinos Chronis et al. with accession number GSE90895 [58]. SEs of the compendium of tissue types used in this study were downloaded from Jiang et al. SEdb: a comprehensive human super-enhancer database (http://www.licpathway.net/sedb) [59]. A list of all used datasets and accession numbers were summarized in Additional file 2: Table S1. The evolving signals of enhancers during cell differentiation for all the interrogated systems in this study are summarized in Additional file 5: Table S4.

## Abbreviations

SE: Super-enhancer
ChIP-seq: Chromatin immune-precipitation followed by high-throughput sequencing
TSS: Transcription start site
Hi-C: Genome conformation capture
TAD: Topologically associating domain
FIREs: Frequently interacting regions
RSD: Relative standard deviation
hESC: Human embryonic stem cells
mESC: Mouse embryonic stem cells
MC: Mesodermal cells
CMC: Cardiac mesodermal cells
CP: Cardiac progenitors
PCM: Primitive cardiomyocytes
CM: Cardiomyocytes
LTHSC: Long-term hematopoietic stem cells
STHSC: Short-term hematopoietic stem cells
MPP: Multipotent progenitor
CMP: Common myeloid progenitor
MEP: Megakaryocyte erythroid progenitor
CLP: Common lymphoid progenitor
GMP: Granulocyte-monocyte progenitor
GN: Granulocyte
MEFs: Mouse embryonic fibroblasts
