# Supplementary material for: Mapping the evolving landscape of super-enhancers during cell differentiation
Source: Genome Biol. 2021 Sep 15;22:269. doi: 10.1186/s13059-021-02485-x (PMC8442463; doi:10.1186/s13059-021-02485-x)
Supplement: Supplementary file 1 — Additional file 1. Supplementary figures (Figure S1 – Figure S10). [file 13059_2021_2485_MOESM1_ESM.pdf]

Fig S1

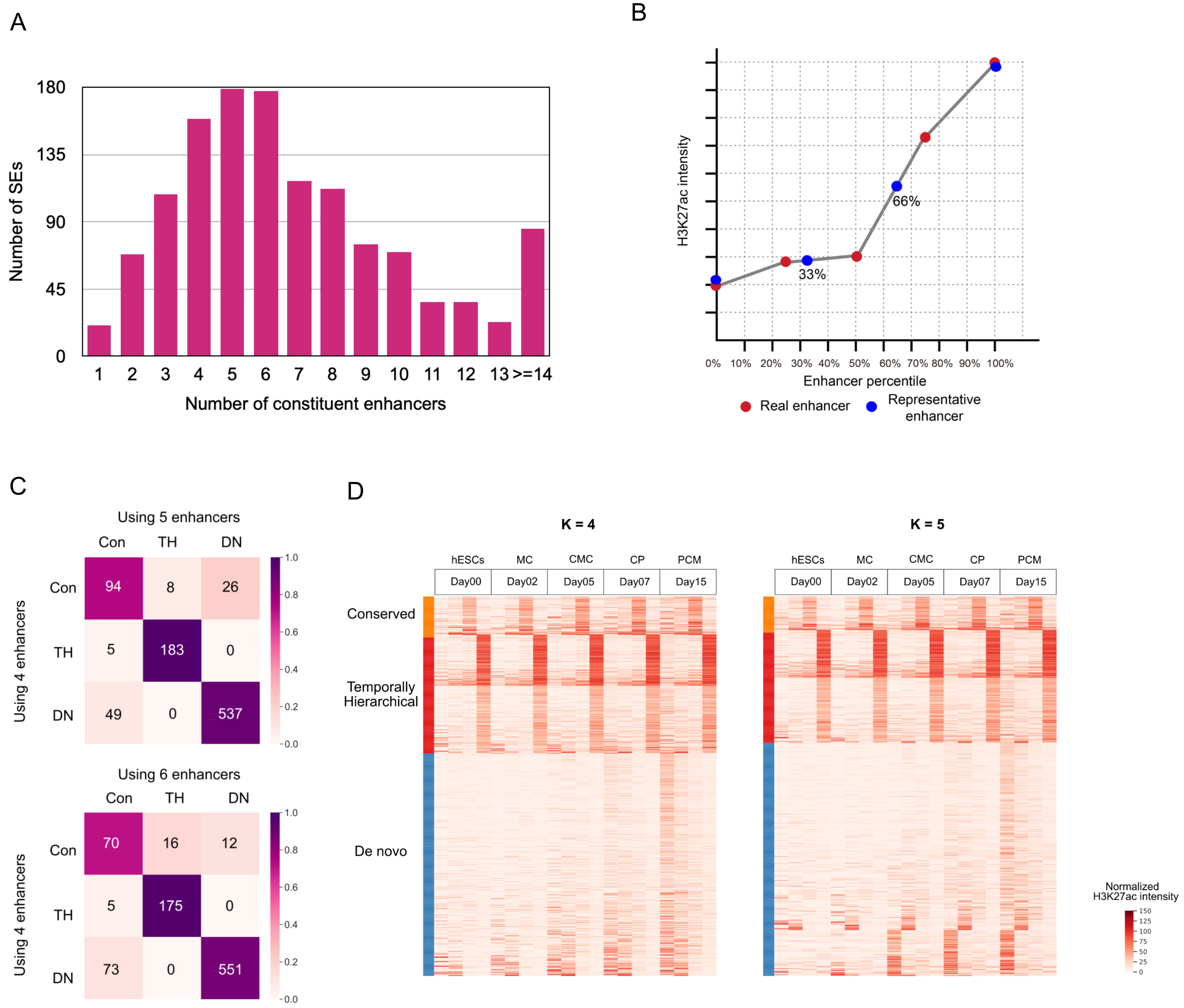

Fig S1 An unbiased approach to classify SEs according to their evolving landscapes during cell differentiation.
a Distribution of the number of constituent enhancers of SEs in Day15 cells.
b Illustration of selecting four representative elements from a SE with 5 elements using a step-wise linear interpolation approach. Constituent elements were ordered by the H3K27ac variation level (Relative Standard Deviation, std/mean) across the differentiation stages from high to low. The elements with the highest and lowest variation were represented by the actual data and the intermediate two elements were represented by step-wise linear interpolation.
c Confusion matrices representing the SE classification differences between using different numbers of representative enhancers. The numbers in each square represent the number of SEs, and the color of the square indicates the percentage of SEs in each row.
d Heatmaps showing the K-means clustering of SEs using different choices of K.

Fig S2

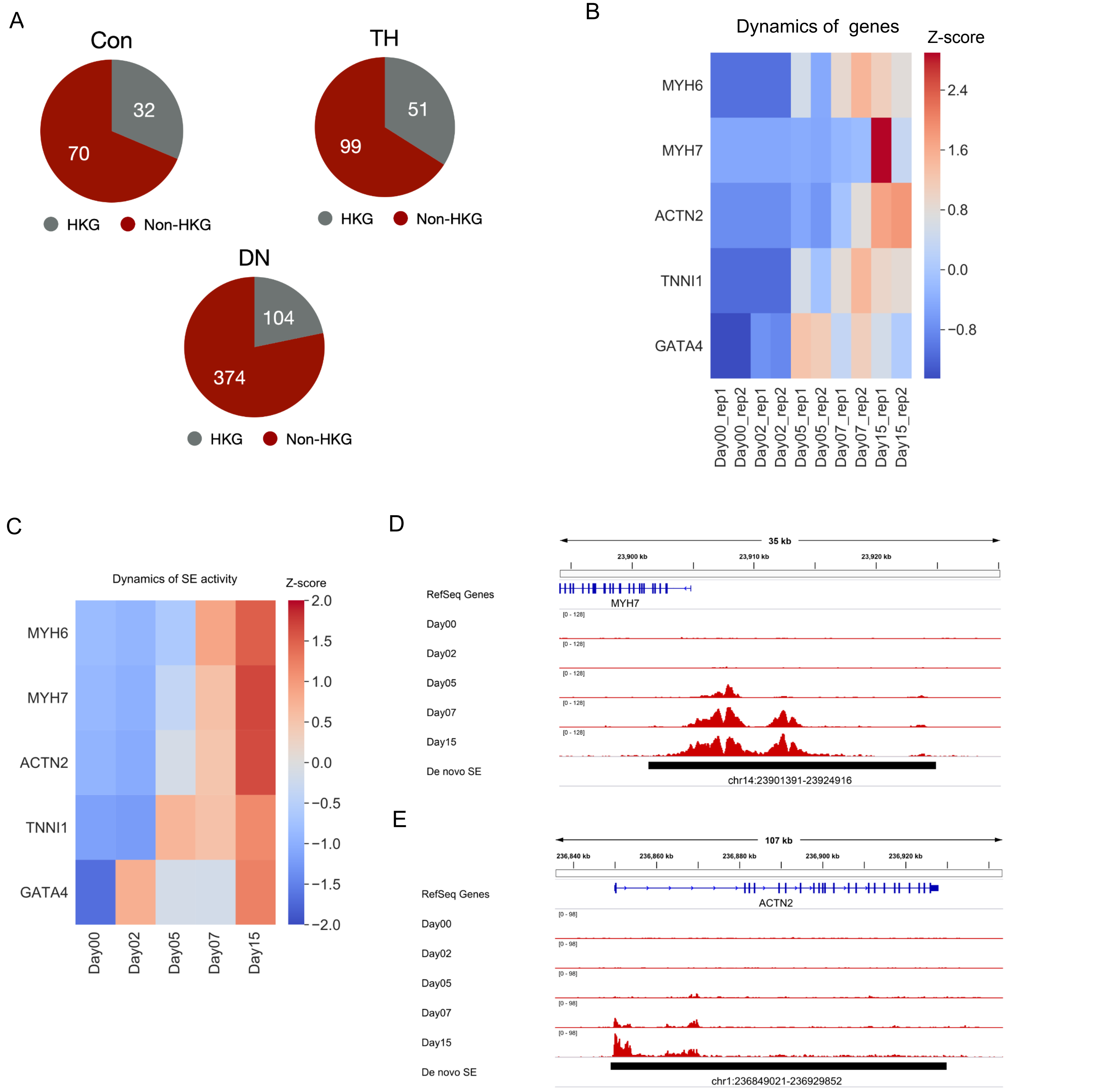

Fig S2 Dynamic activities of the three types of SEs and their associated genes during human cardiomyocyte differentiation.
a Overlapping between genes associated with the three types of SEs with house-keeping genes (HKGs).
b Gene expression profiles of selected cardiomyocyte marker genes that are associated with DN SEs. Z-score values during differentiation are shown.
c Z-score matrix showing the dynamics of the SEs associated with the selected cardiomyocyte marker genes.
d-e Genome browser screenshot for a DN SE associated with cardiomyocyte marker gene *MYH7* (**d**) and *ACTN2* (e).

Fig S3

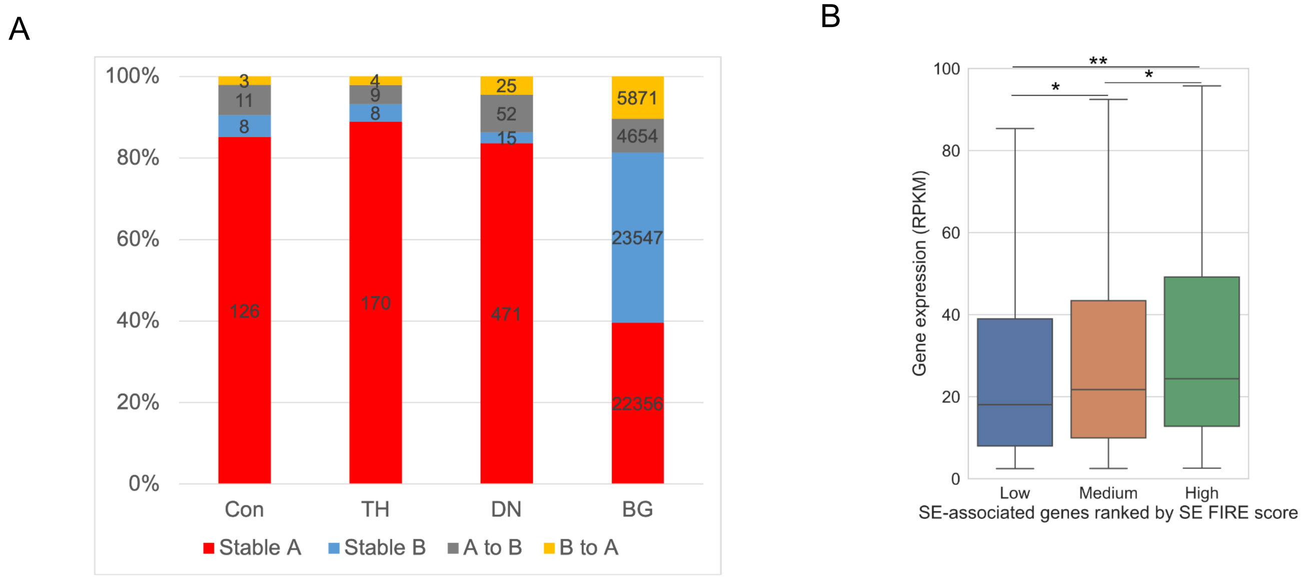

Fig S3 Higher-order chromatin features of the three types of SEs.
a Stacked bar chart showing the number of three types of SEs associated with different compartment types. Numbers in the 'BG' group (whole genome background) represent the total count of 50kb bins for each compartment type.
b Genes associated with SEs with higher FIRE scores have higher expression levels.
*, p<0.05, **, p < 0.01, Mann Whitney U test.

Fig S4

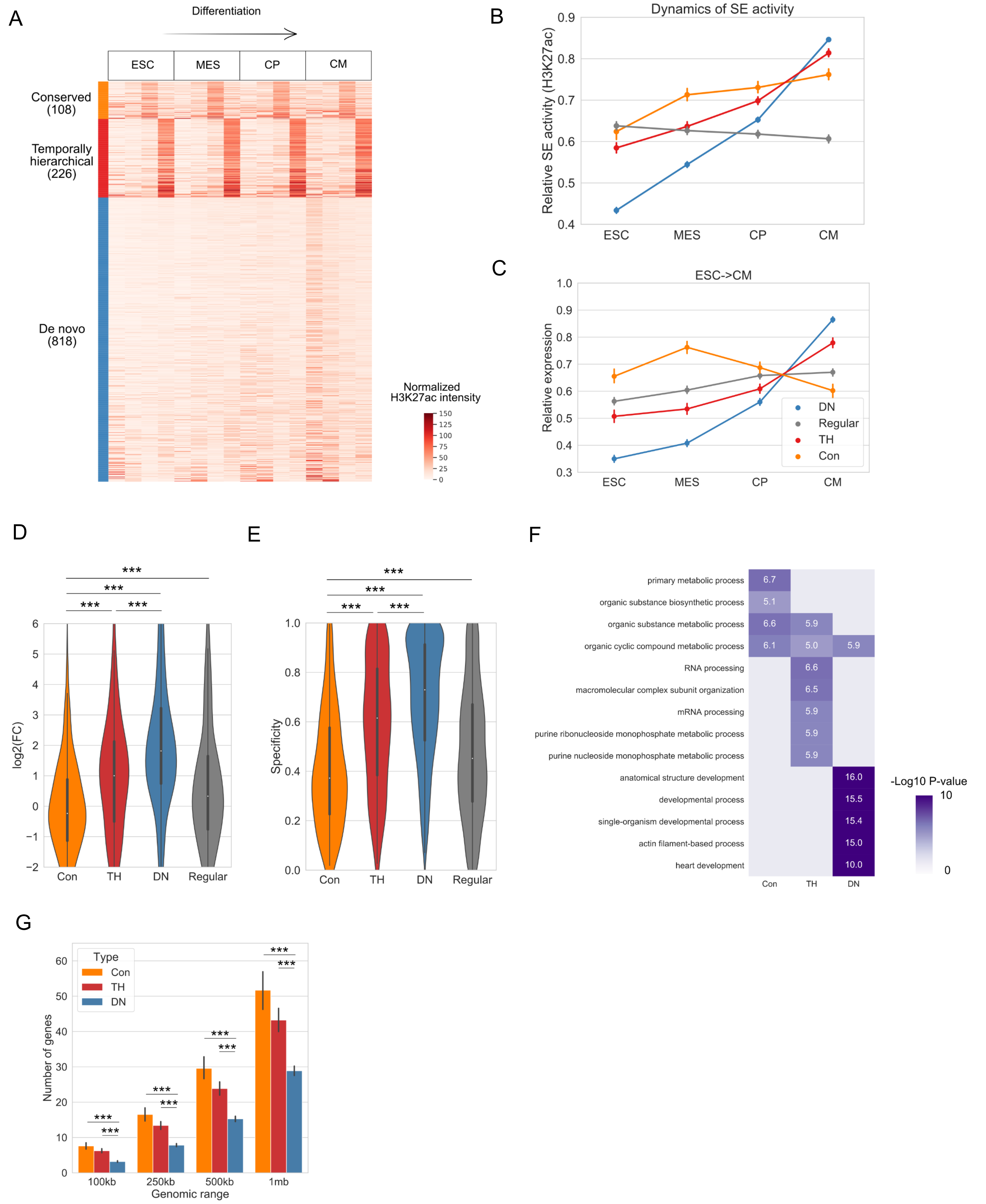

A
Differentiation
ESC
MES
CP
CM
Conserved (108)
Temporally hierarchical (226)
De novo (818)
Normalized H3K27ac intensity
150
125
100
75
50
25
0
B
Dynamics of SE activity
Relative SE activity (H3K27ac)
0.9
0.8
0.7
0.6
0.5
0.4
ESC
MES
CP
CM
C
ESC->CM
Relative expression
1.0
0.9
0.8
0.7
0.6
0.5
0.4
0.3
DN
Regular
TH
Con
ESC
MES
CP
CM
D
***
***
***
***
log2(FC)
6
5
4
3
2
1
0
−1
−2
Con
TH
DN
Regular
E
***
***
***
***
Specificity
1.0
0.8
0.6
0.4
0.2
0.0
Con
TH
DN
Regular
F
primary metabolic process
organic substance biosynthetic process
organic substance metabolic process
organic cyclic compound metabolic process
RNA processing
macromolecular complex subunit organization
mRNA processing
purine ribonucleoside monophosphate metabolic process
purine nucleoside monophosphate metabolic process
anatomical structure development
developmental process
single-organism developmental process
actin filament-based process
heart development
6.7
5.1
6.6
5.9
6.1
5.0
5.9
6.6
6.5
5.9
5.9
5.9
16.0
15.5
15.4
15.0
10.0
Con
TH
DN
-Log10 P-value
10
0
G
Type
Con
TH
DN
Number of genes
60
50
40
30
20
10
0
***
***
100kb
250kb
500kb
1mb
Genomic range

Fig S4 Mapping the evolving landscapes of SEs in mouse cardiomyocytes reveals similar patterns.

a Heatmap showing the three sub-classes of SEs defined by progressing landscape during differentiation.

b Dynamics of average SE activity during differentiation. For each SE, its signals during differentiation were represented by the signals relative to the maximum H3K27ac intensity across differentiation stages.

c Dynamics of genes associated with the three types of SEs. For each gene, its expressions during differentiation were represented by the expression values relative to the maximum expression across the stages.

d-e Violin plot showing the fold changes (CM/ESC) (**d**) and specificity (**e**) of genes associated with the three types of SEs.

f GO analysis showing the enriched functions for the three types of SEs.

g Comparison of gene density near the three types of SEs. *, $p < 0.05$, **, $p < 0.01$, ***, $p < 0.001$, Mann-Whitney U test.

Fig S5

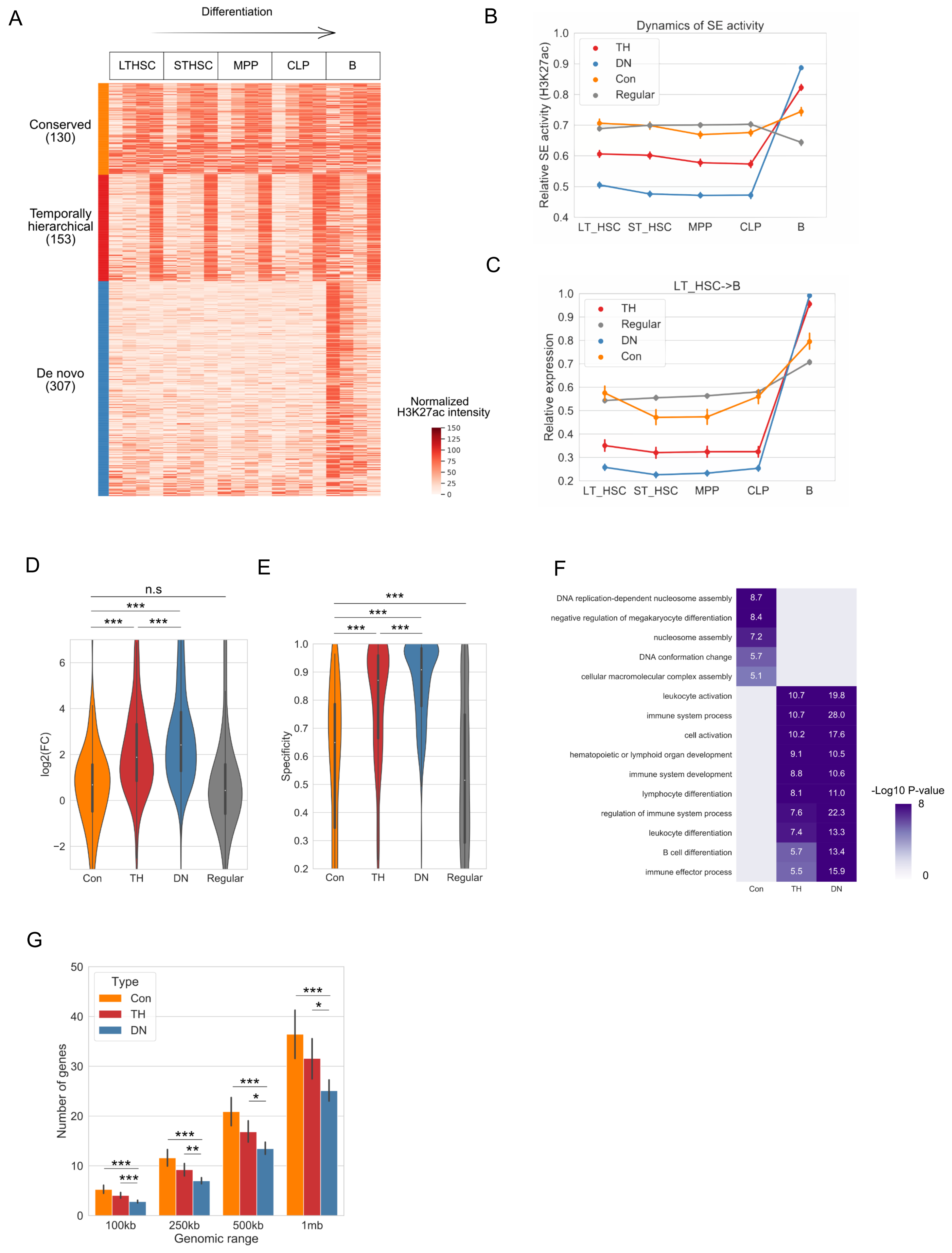
A
Differentiation
LTHSC
STHSC
MPP
CLP
B
Conserved (130)
Temporally hierarchical (153)
De novo (307)
Normalized H3K27ac intensity
150
125
100
75
50
25
0
B
Dynamics of SE activity
Relative SE activity (H3K27ac)
TH
DN
Con
Regular
LT_HSC
ST_HSC
MPP
CLP
B
C
LT_HSC->B
Relative expression
TH
Regular
DN
Con
LT_HSC
ST_HSC
MPP
CLP
B
D
n.s
***
log2(FC)
Con
TH
DN
Regular
E
***
Specificity
Con
TH
DN
Regular
F
DNA replication-dependent nucleosome assembly 8.7
negative regulation of megakaryocyte differentiation 8.4
nucleosome assembly 7.2
DNA conformation change 5.7
cellular macromolecular complex assembly 5.1
leukocyte activation 10.7 19.8
immune system process 10.7 28.0
cell activation 10.2 17.6
hematopoietic or lymphoid organ development 9.1 10.5
immune system development 8.8 10.6
lymphocyte differentiation 8.1 11.0
regulation of immune system process 7.6 22.3
leukocyte differentiation 7.4 13.3
B cell differentiation 5.7 13.4
immune effector process 5.5 15.9
Con
TH
DN
-Log10 P-value
8
0
G
Type
Con
TH
DN
Number of genes
***
**
*
100kb
250kb
500kb
1mb
Genomic range

Fig S5 Mapping the evolving landscapes of SEs in mouse B cells reveals similar patterns.

a Heatmap showing the three sub-classes of SEs defined by progressing landscape during differentiation.

b Dynamics of average SE activity during differentiation. For each SE, its signals during differentiation were represented by the signals relative to the maximum H3K27ac intensity across differentiation stages.

c Dynamics of genes associated with the three types of SEs. For each gene, its expressions during differentiation were represented by the expression values relative to the maximum expression across the five stages.

d-e Violin plot showing the fold changes (B cells/LTHSC) (**d**) and specificity (**e**) of genes associated with the three types of SEs.

f GO analysis showing the enriched functions for the three types of SEs.

g Comparison of gene density near the three types of SEs. *, $p < 0.05$, **, $p < 0.01$, ***, $p < 0.001$, Mann-Whitney U test.

Fig S6

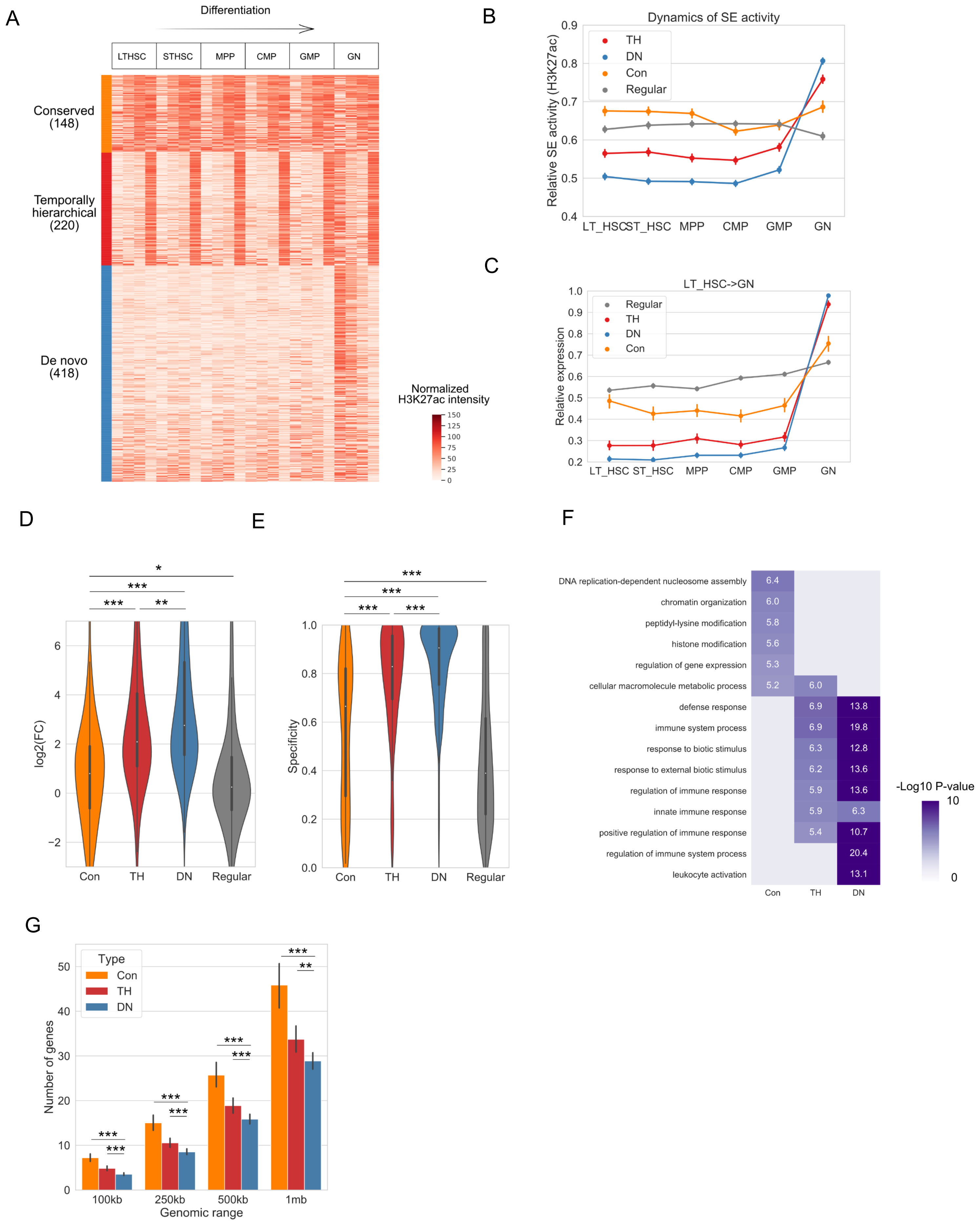
A
Differentiation
LTHSC
STHSC
MPP
CMP
GMP
GN
Conserved (148)
Temporally hierarchical (220)
De novo (418)
Normalized H3K27ac intensity
150
125
100
75
50
25
0
B
Dynamics of SE activity
Relative SE activity (H3K27ac)
TH
DN
Con
Regular
LT_HSC
ST_HSC
MPP
CMP
GMP
GN
C
LT_HSC->GN
Relative expression
Regular
TH
DN
Con
LT_HSC
ST_HSC
MPP
CMP
GMP
GN
D
log2(FC)
Con
TH
DN
Regular
E
Specificity
Con
TH
DN
Regular
F
DNA replication-dependent nucleosome assembly
chromatin organization
peptidyl-lysine modification
histone modification
regulation of gene expression
cellular macromolecule metabolic process
defense response
immune system process
response to biotic stimulus
response to external biotic stimulus
regulation of immune response
innate immune response
positive regulation of immune response
regulation of immune system process
leukocyte activation
6.4
6.0
5.8
5.6
5.3
5.2
6.0
6.9
13.8
6.9
19.8
6.3
12.8
6.2
13.6
5.9
13.6
5.9
6.3
5.4
10.7
20.4
13.1
Con
TH
DN
-Log10 P-value
10
0
G
Type
Con
TH
DN
Number of genes
100kb
250kb
500kb
1mb
Genomic range

Fig S6 Mapping the evolving landscapes of SEs in mouse granulocytes reveals similar patterns.
a Heatmap showing the three sub-classes of SEs defined by progressing landscape during differentiation.
b Dynamics of average SE activity during differentiation. For each SE, its signals during differentiation were represented by the signals relative to the maximum H3K27ac intensity across differentiation stages.
c Dynamics of genes associated with the three types of SEs. For each gene, its expressions during differentiation were represented by the expression values relative to the maximum expression across stages.
d-e Violin plot showing the fold changes (GN/LTHSC) (**d**) and specificity (**e**) of genes associated with the three types of SEs.
f GO analysis showing the enriched functions for the three types of SEs.
g Comparison of gene density near the three types of SEs. *, $p < 0.05$, **, $p < 0.01$, ***, $p < 0.001$, Mann-Whitney U test.

Fig S7

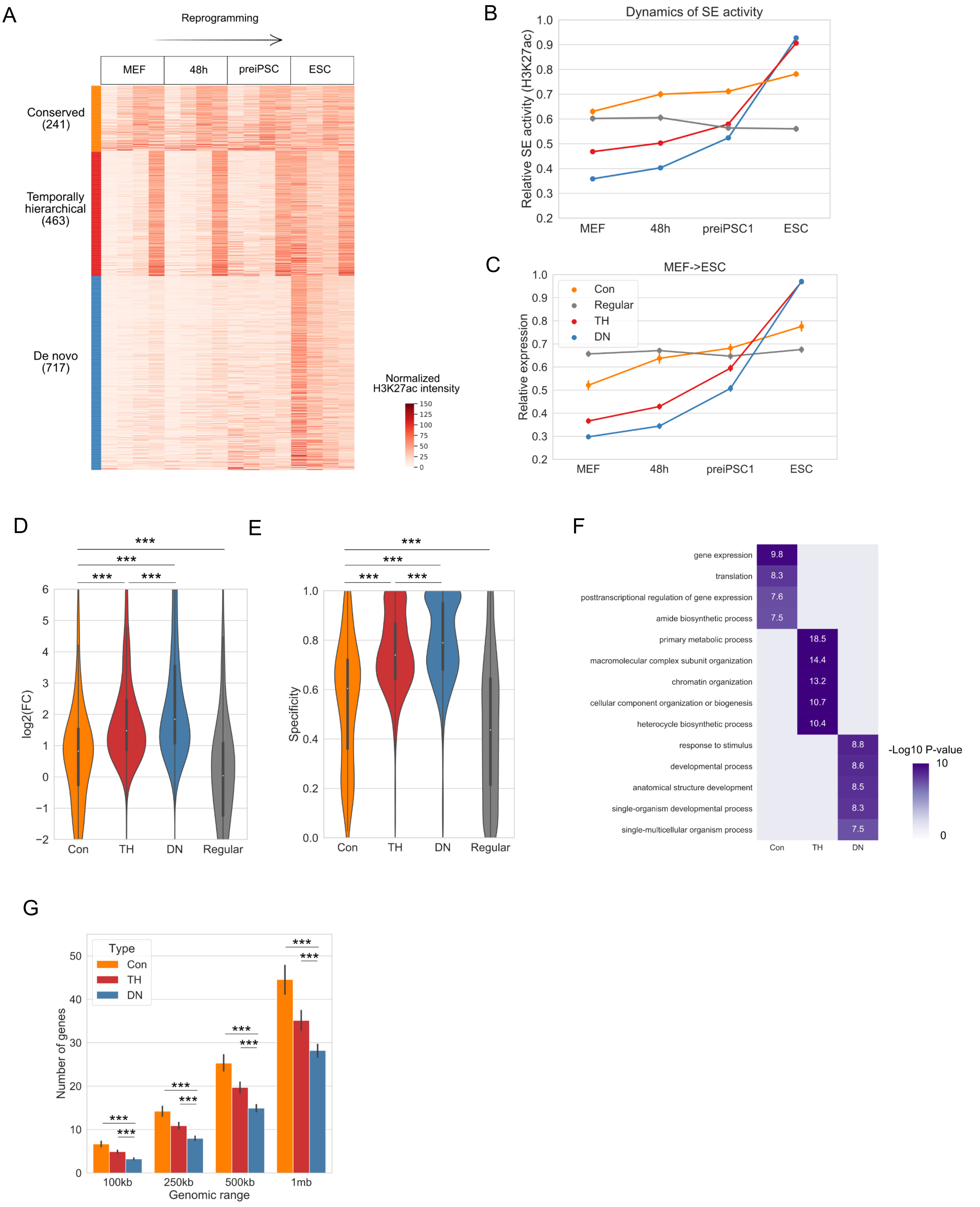
A
Reprogramming
MEF
48h
preiPSC
ESC
Conserved (241)
Temporally hierarchical (463)
De novo (717)
Normalized H3K27ac intensity
150
125
100
75
50
25
0
B
Dynamics of SE activity
Relative SE activity (H3K27ac)
1.0
0.9
0.8
0.7
0.6
0.5
0.4
0.3
0.2
MEF
48h
preiPSC1
ESC
C
MEF->ESC
Con
Regular
TH
DN
Relative expression
1.0
0.9
0.8
0.7
0.6
0.5
0.4
0.3
0.2
MEF
48h
preiPSC1
ESC
D
***
***
***
***
log2(FC)
6
5
4
3
2
1
0
−1
−2
Con
TH
DN
Regular
E
***
***
***
***
Specificity
1.0
0.8
0.6
0.4
0.2
0.0
Con
TH
DN
Regular
F
gene expression 9.8
translation 8.3
posttranscriptional regulation of gene expression 7.6
amide biosynthetic process 7.5
primary metabolic process 18.5
macromolecular complex subunit organization 14.4
chromatin organization 13.2
cellular component organization or biogenesis 10.7
heterocycle biosynthetic process 10.4
response to stimulus 8.8
developmental process 8.6
anatomical structure development 8.5
single-organism developmental process 8.3
single-multicellular organism process 7.5
Con
TH
DN
-Log10 P-value
10
0
G
Type
Con
TH
DN
***
***
Number of genes
50
40
30
20
10
0
100kb
250kb
500kb
1mb
Genomic range

Fig S7 The observed patterns are generalizable to reprogramming.
a Heatmap showing the three sub-classes of SEs defined by progressing landscape during differentiation.
b Dynamics of average SE activity during differentiation. For each SE, its signals during differentiation were represented by the signals relative to the maximum H3K27ac intensity across differentiation stages.
c Dynamics of genes associated with the three types of SEs. For each gene, its expressions during differentiation were represented by the expression values relative to the maximum expression across the four stages.
d-e Violin plot showing the fold changes (ESC/MEF) (**d**) and specificity (**e**) of genes associated with the three types of SEs.
f GO analysis showing the enriched functions for the three types of SEs.
g Comparison of gene density near the three types of SEs. *, $p < 0.05$, **, $p < 0.01$, ***, $p < 0.001$, Mann-Whitney U test.

Fig S8

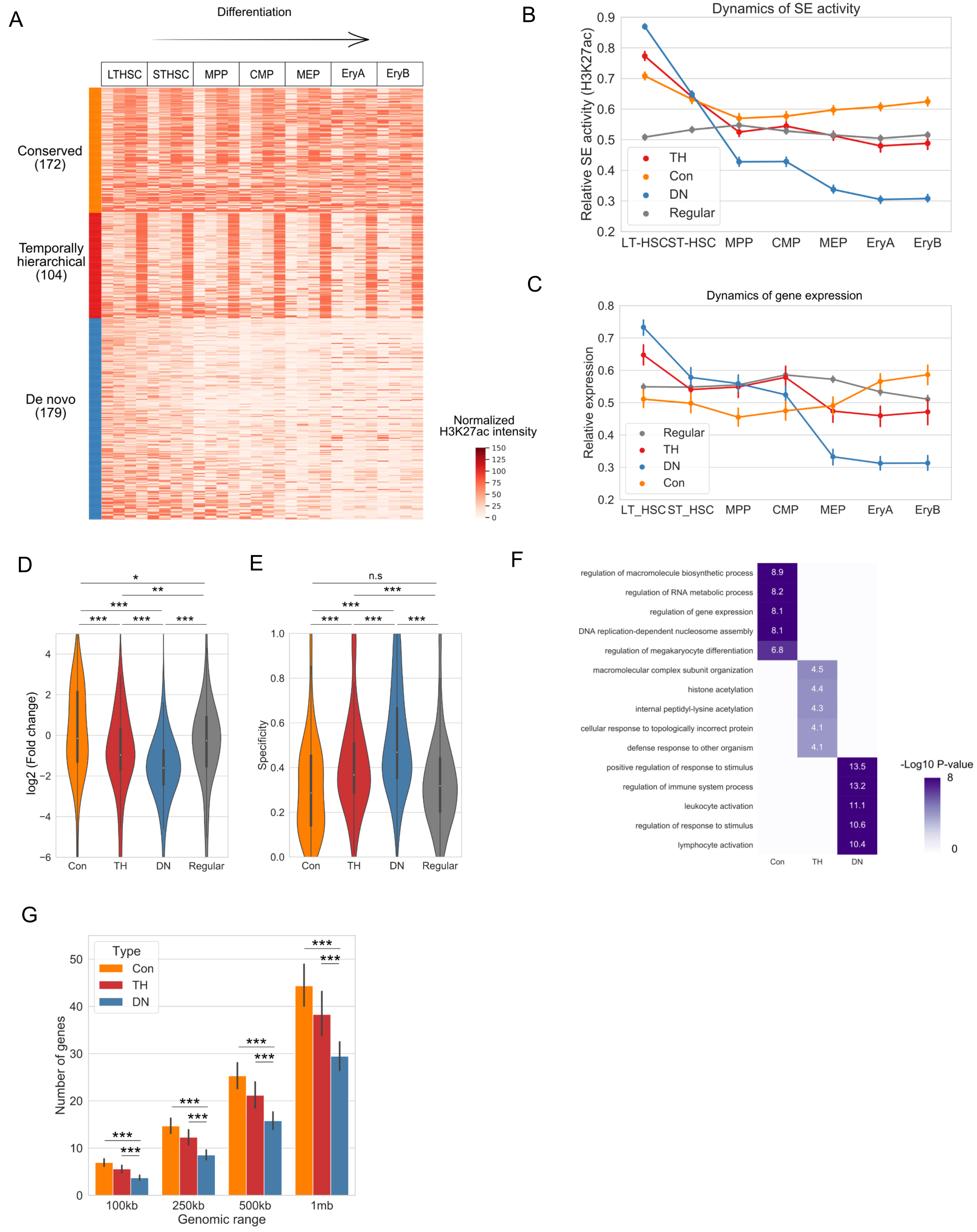
A
Differentiation
LTHSC
STHSC
MPP
CMP
MEP
EryA
EryB
Conserved (172)
Temporally hierarchical (104)
De novo (179)
Normalized H3K27ac intensity
B
Dynamics of SE activity
Relative SE activity (H3K27ac)
TH
Con
DN
Regular
LT-HSC
ST-HSC
MPP
CMP
MEP
EryA
EryB
C
Dynamics of gene expression
Relative expression
Regular
TH
DN
Con
LT_HSC
ST_HSC
MPP
CMP
MEP
EryA
EryB
D
log2 (Fold change)
Con
TH
DN
Regular
E
n.s
Specificity
Con
TH
DN
Regular
F
regulation of macromolecule biosynthetic process
regulation of RNA metabolic process
regulation of gene expression
DNA replication-dependent nucleosome assembly
regulation of megakaryocyte differentiation
macromolecular complex subunit organization
histone acetylation
internal peptidyl-lysine acetylation
cellular response to topologically incorrect protein
defense response to other organism
positive regulation of response to stimulus
regulation of immune system process
leukocyte activation
regulation of response to stimulus
lymphocyte activation
-Log10 P-value
Con
TH
DN
G
Type
Con
TH
DN
Number of genes
100kb
250kb
500kb
1mb
Genomic range

Fig S8 Decommission of SEs in hematopoietic stem cells follows a similar pattern as we observed in SE activation.

a Heatmap showing the three sub-classes of SEs defined by progressing landscape during differentiation.

b Dynamics of average SE activity during differentiation. For each SE, its signals during differentiation were represented by the signals relative to the maximum H3K27ac intensity across differentiation stages.

c Dynamics of genes associated with the three types of SEs. For each gene, its expressions during differentiation were represented by the expression values relative to the maximum expression across stages.

d-e Violin plot showing the fold changes (EryB/LTHSC) (d) and specificity (e) of genes associated with the three types of SEs.

f GO analysis showing the enriched functions for the three types of SEs.

g Comparison of gene density near the three types of SEs. *, $p < 0.05$, **, $p < 0.01$, ***, $p < 0.001$, Mann-Whitney U test.

Fig S9

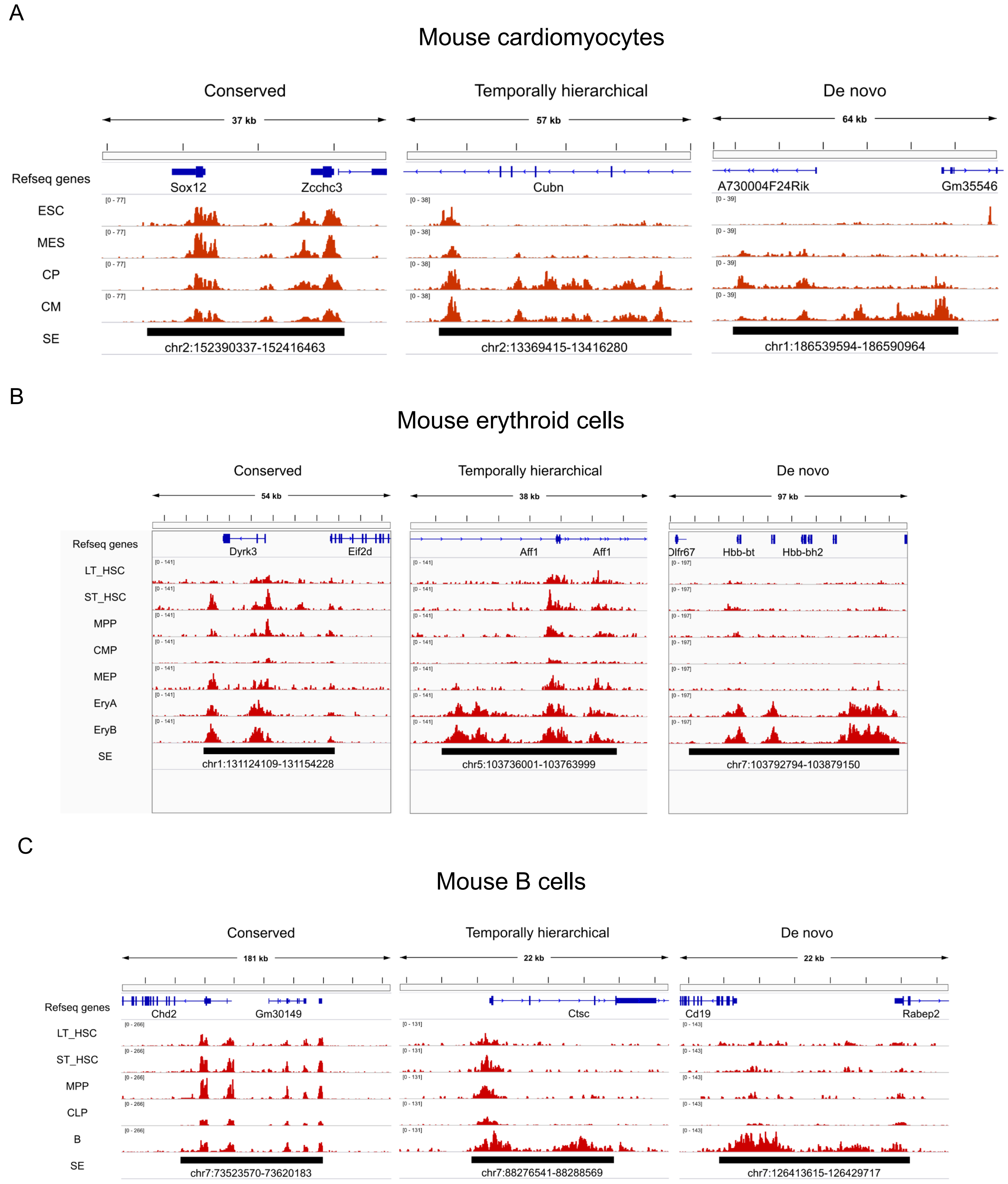
A
Mouse cardiomyocytes
Conserved
Temporally hierarchical
De novo
37 kb
57 kb
64 kb
Refseq genes
Sox12
Zcchc3
Cubn
A730004F24Rik
Gm35546
ESC
MES
CP
CM
SE
[0 - 77]
[0 - 38]
[0 - 39]
chr2:152390337-152416463
chr2:13369415-13416280
chr1:186539594-186590964
B
Mouse erythroid cells
Conserved
Temporally hierarchical
De novo
54 kb
38 kb
97 kb
Refseq genes
Dyrk3
Eif2d
Aff1
Aff1
Olfr67
Hbb-bt
Hbb-bh2
LT_HSC
ST_HSC
MPP
CMP
MEP
EryA
EryB
SE
[0 - 141]
[0 - 197]
chr1:131124109-131154228
chr5:103736001-103763999
chr7:103792794-103879150
C
Mouse B cells
Conserved
Temporally hierarchical
De novo
181 kb
22 kb
22 kb
Refseq genes
Chd2
Gm30149
Ctsc
Cd19
Rabep2
LT_HSC
ST_HSC
MPP
CLP
B
SE
[0 - 266]
[0 - 131]
[0 - 143]
chr7:73523570-73620183
chr7:88276541-88288569
chr7:126413615-126429717

Fig S9

D

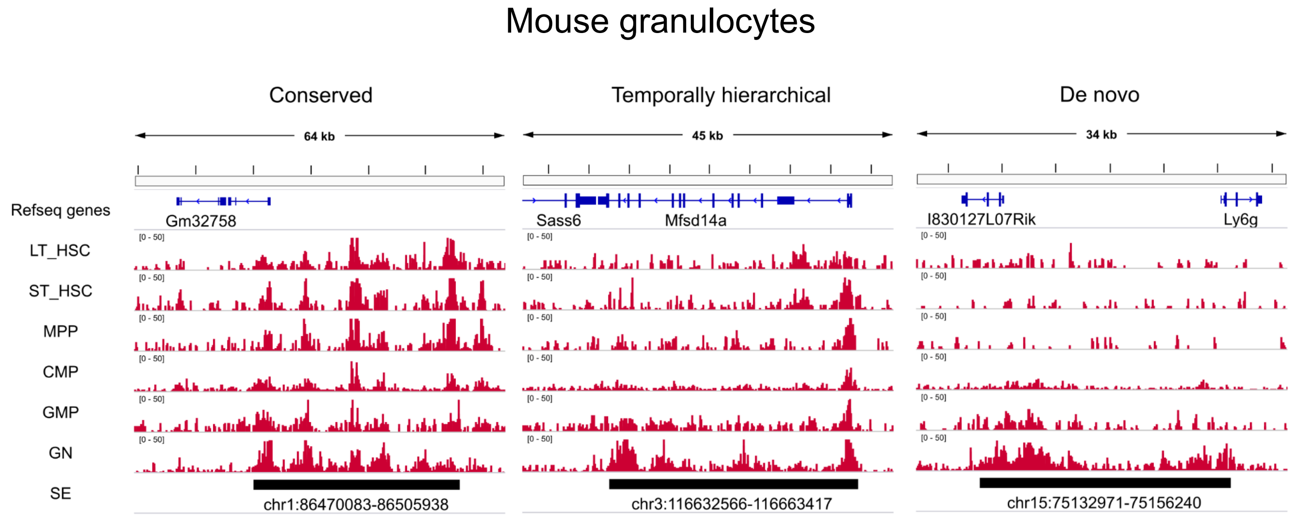
Mouse granulocytes
Conserved
Temporally hierarchical
De novo
64 kb
45 kb
34 kb
Refseq genes
Gm32758
Sass6
Mfsd14a
I830127L07Rik
Ly6g
LT_HSC
ST_HSC
MPP
CMP
GMP
GN
SE
chr1:86470083-86505938
chr3:116632566-116663417
chr15:75132971-75156240

E

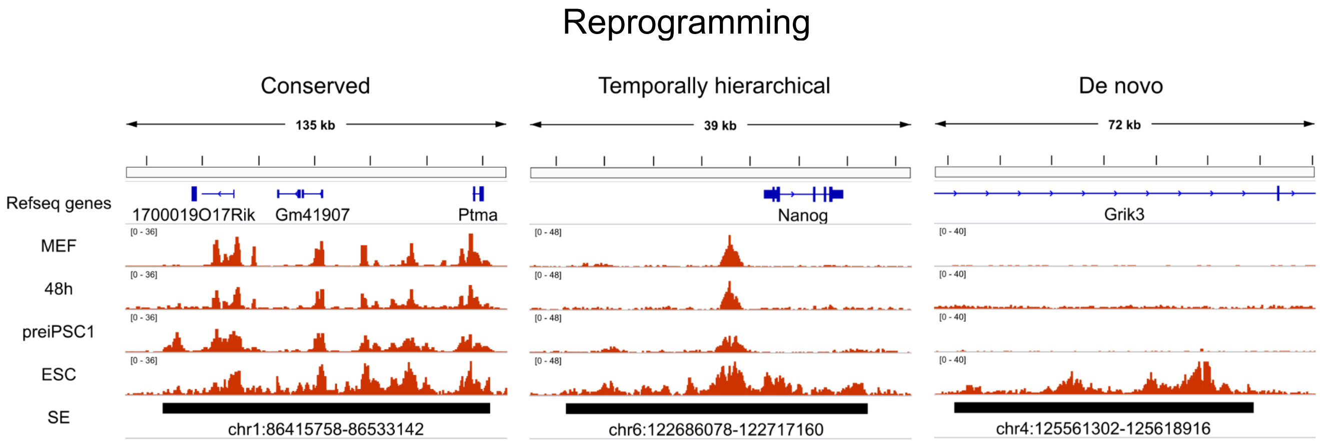
Reprogramming
Conserved
Temporally hierarchical
De novo
135 kb
39 kb
72 kb
Refseq genes
1700019O17Rik
Gm41907
Ptma
Nanog
Grik3
MEF
48h
preiPSC1
ESC
SE
chr1:86415758-86533142
chr6:122686078-122717160
chr4:125561302-125618916

F

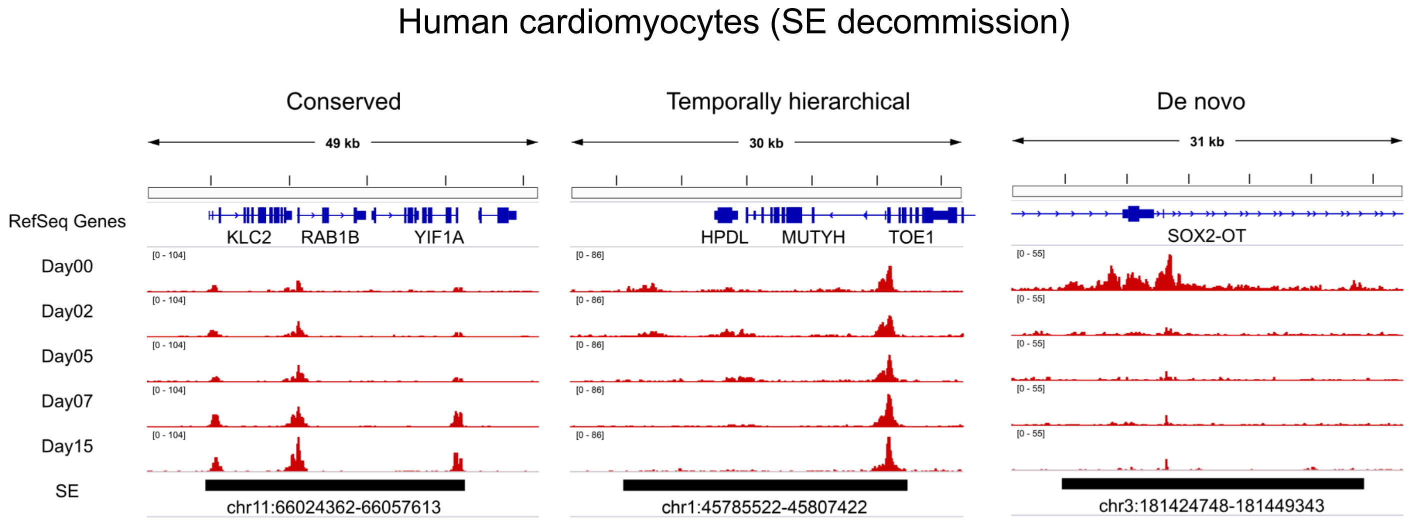
Human cardiomyocytes (SE decommission)
Conserved
Temporally hierarchical
De novo
49 kb
30 kb
31 kb
RefSeq Genes
KLC2
RAB1B
YIF1A
HPDL
MUTYH
TOE1
SOX2-OT
Day00
Day02
Day05
Day07
Day15
SE
chr11:66024362-66057613
chr1:45785522-45807422
chr3:181424748-181449343

Fig S9

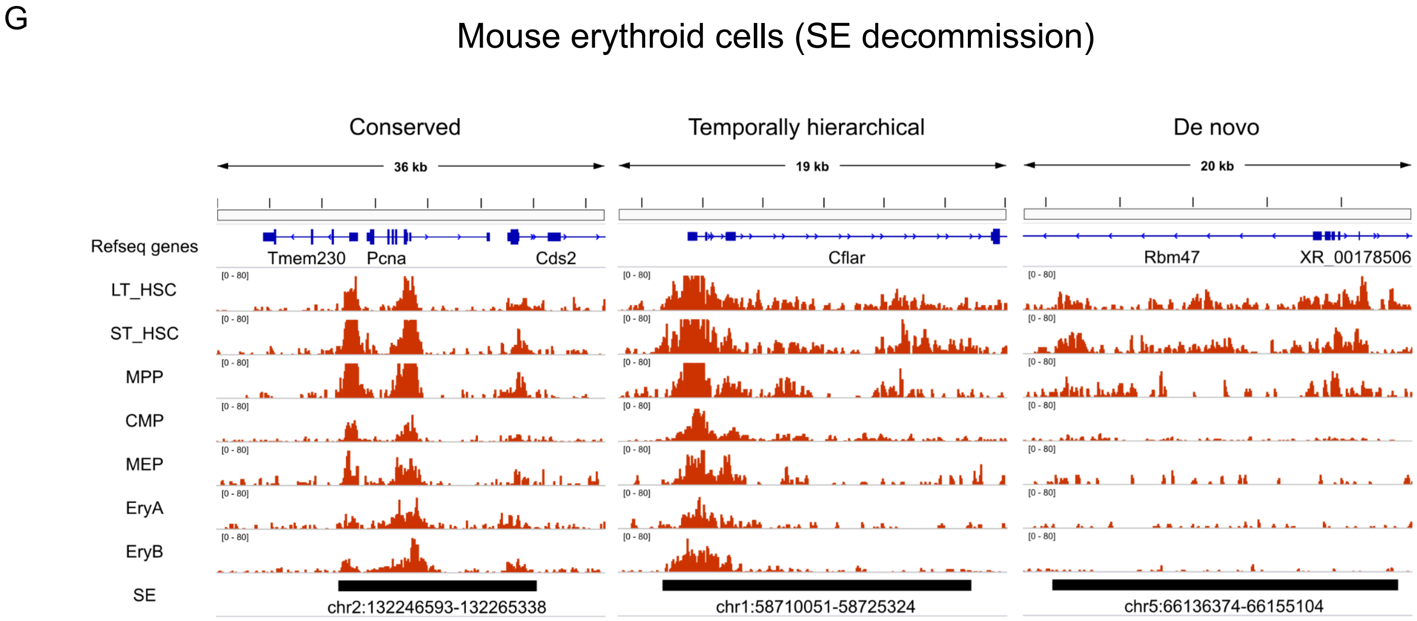

Fig S9 Sample genome browser tracks showing the typical SEs for each temporal pattern.

Fig S10

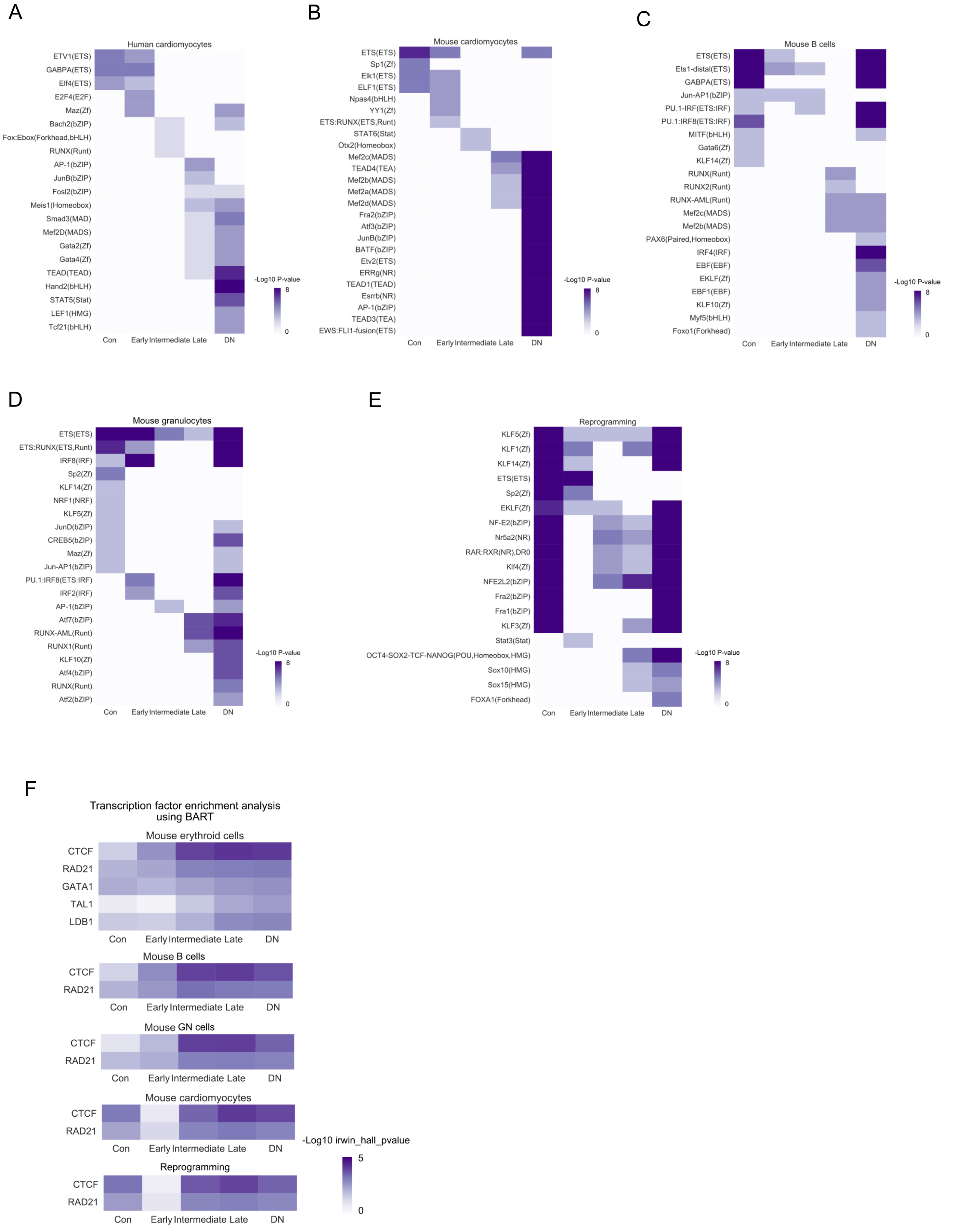

A
Human cardiomyocytes
ETV1(ETS)
GABPA(ETS)
Elf4(ETS)
E2F4(E2F)
Maz(Zf)
Bach2(bZIP)
Fox:Ebox(Forkhead,bHLH)
RUNX(Runt)
AP-1(bZIP)
JunB(bZIP)
Fosl2(bZIP)
Meis1(Homeobox)
Smad3(MAD)
Mef2D(MADS)
Gata2(Zf)
Gata4(Zf)
TEAD(TEAD)
Hand2(bHLH)
STAT5(Stat)
LEF1(HMG)
Tcf21(bHLH)
Con
Early Intermediate Late
DN
-Log10 P-value
8
0
B
Mouse cardiomyocytes
ETS(ETS)
Sp1(Zf)
Elk1(ETS)
ELF1(ETS)
Npas4(bHLH)
YY1(Zf)
ETS:RUNX(ETS,Runt)
STAT6(Stat)
Otx2(Homeobox)
Mef2c(MADS)
TEAD4(TEA)
Mef2b(MADS)
Mef2a(MADS)
Mef2d(MADS)
Fra2(bZIP)
Atf3(bZIP)
JunB(bZIP)
BATF(bZIP)
Etv2(ETS)
ERRg(NR)
TEAD1(TEAD)
Esrrb(NR)
AP-1(bZIP)
TEAD3(TEA)
EWS:FLI1-fusion(ETS)
Con
Early Intermediate Late
DN
-Log10 P-value
8
0
C
Mouse B cells
ETS(ETS)
Ets1-distal(ETS)
GABPA(ETS)
Jun-AP1(bZIP)
PU.1-IRF(ETS:IRF)
PU.1:IRF8(ETS:IRF)
MITF(bHLH)
Gata6(Zf)
KLF14(Zf)
RUNX(Runt)
RUNX2(Runt)
RUNX-AML(Runt)
Mef2c(MADS)
Mef2b(MADS)
PAX6(Paired,Homeobox)
IRF4(IRF)
EBF(EBF)
EKLF(Zf)
EBF1(EBF)
KLF10(Zf)
Myf5(bHLH)
Foxo1(Forkhead)
Con
Early Intermediate Late
DN
-Log10 P-value
8
0
D
Mouse granulocytes
ETS(ETS)
ETS:RUNX(ETS,Runt)
IRF8(IRF)
Sp2(Zf)
KLF14(Zf)
NRF1(NRF)
KLF5(Zf)
JunD(bZIP)
CREB5(bZIP)
Maz(Zf)
Jun-AP1(bZIP)
PU.1:IRF8(ETS:IRF)
IRF2(IRF)
AP-1(bZIP)
Atf7(bZIP)
RUNX-AML(Runt)
RUNX1(Runt)
KLF10(Zf)
Atf4(bZIP)
RUNX(Runt)
Atf2(bZIP)
Con
Early Intermediate Late
DN
-Log10 P-value
8
0
E
Reprogramming
KLF5(Zf)
KLF1(Zf)
KLF14(Zf)
ETS(ETS)
Sp2(Zf)
EKLF(Zf)
NF-E2(bZIP)
Nr5a2(NR)
RAR:RXR(NR),DR0
Klf4(Zf)
NFE2L2(bZIP)
Fra2(bZIP)
Fra1(bZIP)
KLF3(Zf)
Stat3(Stat)
OCT4-SOX2-TCF-NANOG(POU,Homeobox,HMG)
Sox10(HMG)
Sox15(HMG)
FOXA1(Forkhead)
Con
Early Intermediate Late
DN
-Log10 P-value
8
0
F
Transcription factor enrichment analysis using BART
Mouse erythroid cells
CTCF
RAD21
GATA1
TAL1
LDB1
Con
Early Intermediate Late
DN
Mouse B cells
CTCF
RAD21
Con
Early Intermediate Late
DN
Mouse GN cells
CTCF
RAD21
Con
Early Intermediate Late
DN
Mouse cardiomyocytes
CTCF
RAD21
Con
Early Intermediate Late
DN
Reprogramming
CTCF
RAD21
Con
Early Intermediate Late
DN
-Log10 irwin_hall_pvalue
5
0

Fig S10 Heatmap showing the differential motif enrichment pattern for the three types of elements in TH SEs as well as the elements in DN SEs. Color represents the –log10 P-value.

a-b Motif enrichment pattern for cardiomyocytes in human and mouse. The specific elements are enriched in cardiomyocyte-specific factors, such as MEF2D and TEAD.

c Motif enrichment pattern for B cells. Specific elements in mouse B cells are enriched in B cell-specific factors (e.g RUNX).

d Motif enrichment pattern for GN cells. Specific elements in GN cells are enriched in factors specific to GN (e.g. NFE2L2 and Pax7).

e Motif enrichment pattern for induced ESCs. Early elements were enriched in Myc, while the late elements were enriched in "OCT3-SOX2-TCF-NANOG". This motif enrichment pattern is consistent with previous studies reporting the two waves of transcription during somatic reprogramming, where the first transcriptional wave is mediated by c-Myc in all cells whereas the second wave is conducted via the expression of Oct4 and Sox2 targets in reprogrammable cells.

f BART analysis reveals that architectural proteins CTCF and RAD21 are more enriched in late enhancers than early enhancers.
