# Supplementary material for: Mapping the evolving landscape of super-enhancers during cell differentiation
Source: Genome Biol. 2021 Sep 15;22:269. doi: 10.1186/s13059-021-02485-x (PMC8442463; doi:10.1186/s13059-021-02485-x)
Supplement: Supplementary file 3 — Additional file 3: Table S2. List of target enhancers, guide RNAs sequences, and genotyping PCR primers. [file 13059_2021_2485_MOESM3_ESM.pdf]

Table S2: List of target enhancers, guide RNAs sequences, and genotyping PCR primers

Enhancer	Genomic coordinates		gRNA sequence	Flanking primers	Internal primers
Early (*Dnajb12*)	chr10: 59099303-59900057	**5’**	CAAGAAATACAGCTTCTCAG	CCACCCTGGCCTAGAGAGAT	CTGACACCAGCCCTAGATGG
		**3’**	AGATAGCAGGTAGGTCCCTG	GAAAGGGCTGACTTCACAGC	CCTGACACCAGCCCTAGATG
Late (*Dnajb12*)	chr10: 59873182-59873967	**5’**	GAAGAAGACTGTGGTAACAG	CCACCCTGGCCTAGAGAGAT	CTGACACCAGCCCTAGATGG
		**3’**	CACTTTAACCCCATTCCCCG	GAAAGGGCTGACTTCACAGC	TAACAAGTGGCAGCTTGCAG
