# Supplementary material for: Mapping the evolving landscape of super-enhancers during cell differentiation
Source: Genome Biol. 2021 Sep 15;22:269. doi: 10.1186/s13059-021-02485-x (PMC8442463; doi:10.1186/s13059-021-02485-x)
Supplement: Supplementary file 4 — Additional file 4: Table S3. List of qRT-PCR primers used for experimental validation. [file 13059_2021_2485_MOESM4_ESM.pdf]

Table S3: qRT-PCR primers

Gene Name	Gene ID	Accession #	Forward primer	Reverse primer
Aff1	17355	NM_133919.4	GAAGGAAAGACGCAACCAAGA	TAGCTCATCGCCTTTTGCAGT
Ddit4	74747	NM_029083.2	CAAGGCAAGAGCTGCCATAG	CCGGTACTTAGCGTCAGGG
Dnajb12	56709	NM_019965.3	CACGAGTCAGCGCACTGAT	TGGGTAGTGTCTGTGGGTTGA
Micu1	216001	NM_001359267.1	ACACCCTCAAGTCTGGCTTAT	TTCCCATCTTTGAAGTGCTTCTT
