# Supplementary material for: Mapping the evolving landscape of super-enhancers during cell differentiation
Source: Genome Biol. 2021 Sep 15;22:269. doi: 10.1186/s13059-021-02485-x (PMC8442463; doi:10.1186/s13059-021-02485-x)
Supplement: Supplementary file 6 — Additional file 6. Review History. [file 13059_2021_2485_MOESM6_ESM.docx]

**Review history**

**First round of review**

**Reviewer 1**

The manuscript "Mapping the Evolving Landscape of Super-Enhancers During Cell Differentiation" by Yan Kai and colleagues focuses on the dynamic restructuring of super-enhancers (SEs) in differentiating cardiomyocytes. The authors propose clustering SEs into three groups based on their presence during development - conserved, temporarily hierarchical (TH), and de novo. They demonstrate that the acquisition of SEs during development results in elevated expression of their target genes. They also demonstrate that all three classes of SEs are associated with the open chromatin. The observed difference between the three classes of SEs is in the GO annotation of their target genes (with the de novo class being strongly associated with cardiomyocyte-specific gene activity), difference in bound transcription factors (TFs), distance to the transcription start site of their target gene, and local chromatin interactions. The experimental validation of a few enhancers within two SEs confirms the important contribution of individual SE enhancer modules to SE activity. Overall, it's an interesting study focusing on a dynamically-active sub-class of SEs.

Major concerns:

    *     I'm surprised the authors haven't been able to identify a sub-class of SEs that demonstrates a gradual loss of SEs during differentiation, a trend opposite to the TH SEs. Given the well-known activity of SEs during early development, I'd expect this class to exist. If this sub-class does exist, I find it essential to report and contrast it to the TH SE sub-class in order to present a full picture of the dynamic SE landscape changes during differentiation.

    *     Assigning a SE to a single gene and doing so without chromatin contact data is a questionable assumption. SEs are long genomic regions that often span multiple gene loci. It is not uncommon for SEs to regulate multiple genes in their proximity. It might eliminate some concerns with this arbitrary selection of SE target genes, if the authors could demonstrate that an alternative assignment of SEs to all expressed genes in their proximity, for example, wouldn't change the reported results. Using chromatin contact data for establishing the set of target genes for an SE would be an alternative option.

    *     The role of SEs in A/B compartment switching during development is an interesting hypothesis, which finds no validation by this study. All three sub-classes of SEs are strongly enriched in the "Stable A" set and the differences between the three classes are not significant. Therefore, I'd strongly recommend either moving this analysis to Supplemental Materials or reevaluating the reported conclusions, as they are currently rather overenthusiastic and misleading.

    *     One of my main concerns is the lack of focus on the TH SEs dataset, which is the key novelty of the presented study. The effects reported for the two other ("stable") datasets are rather expected and reiterate what has been reported on SEs in the past (elevated gene expression, open chromatin state, multiple chromatin contacts, presence of key activator binding sites, etc, etc). The TH sub-class is novel and represent a dynamic change in SE landscape during development. In the current version of the manuscript, it is characterized as an "intermediate" sub-class effectively "averaging" between the conserved and de novo sub-classes of SEs. There is little if any investigation of the role of TH SEs during development and the essentiality of this sub-class of SEs for differentiation.

    *     Finally, I find the experimental component of the study interesting but rather irrelevant to the presented study itself. While the manuscript focuses on the three sub-classes of SEs, the experimental work seeks to distinguish between dynamically-active individual enhancers within a SE. The experiments focus on early, intermediate, and late enhancers within two selected TH SEs and were performed in a mature cell line. The observed result of the importance of individual enhancers for SE activity aligns well with what had been reported in the past. However, this addresses neither the dynamic activity of TH SEs during development nor even the dynamic activity of individual TH SE enhancers during development. Would the KO of two individual enhancers have the same effect during development when one of the enhancers is supposedly inactive? Would that imply that TH SEs gradually gain strength during development or there is an activation threshold in the number of individual enhancers, which acts as an on/off SE switch?


Minor comments:

    *     Line 175, why DN SEs show a lower enrichment in TAD boundaries as compared to Con and TH SEs but contain more frequently interacting regions? 
    *     Line 279, the authors suggest a correlation between the order of elements and TF binding, but didn't discuss why it is the ETS family and not other TF families is enriched in early enhancers? Is there a functional difference between early and late enhancers? 
    *     Figure 5E, does the notation "EnhB" and "EnhA" implies "early" and "late" enhancers. Please clarify.
    *     Figure 2B, the distribution shows a gradual increase in gene expression associated with the  three sub-classes of SEs? Were there any cases of an opposite trend?
    *     Figure 3D, the color-code legend for is missing.
    *     There are multiple typos throughout the manuscript. The manuscript would definitely benefit from a careful proofreading.

**Reviewer 2**

The manuscript from Kai et al. uses a suite of computational approaches to uncover patterns of super-enhancer (SE) activation during differentiation.  The patterns they are most interested in describe whether SEs emerge as whole units at distinct stages or whether the constituent enhancers of SEs appear in a stepwise manner during a differentiation pathway.  To study these patterns, the authors use published high-throughput sequencing data from a range of differentiation systems.  The approaches applied to these data show that developmental SEs fall into both de novo and temporally hierarchical classes (as well as a third, stable class active in all differentiation stages).  The authors then classify individual enhancers within SEs into their patterns of activity and perform experiments to demonstrate that both constituent enhancers that appear in the middle of the differentiation and constituent enhancers that appear at the final stage matter for end-stage gene expression.

The existence of SEs and their roles in driving cell identity programs is largely well-established.  Indeed, the authors note several efforts at dissecting the relationship of constituent enhancers of SEs, but these have generally been performed at steady state in one cell identity.  The kinetics of SE activation remain less understood.  To that end, this manuscript makes an admirable first step toward understanding how differentiating cells "build" SEs.  The clever computational device used here, namely forcing SE constituents into a four-constituent array, makes biological and computational sense, and the authors use it to make important observations about SE biology.

I nevertheless have concerns about the method itself, largely around a reader's ability to interpret the results from the figures presented.    

MAJOR POINTS:
1) I recognize that the data analyzed in this manuscript are in public repositories, but I wonder if the authors can briefly comment on whether each system has distinct biological stages with intermediate, meta-stable cell identities or whether each system is actually an artificial dissection of what is really a continuum of heterogeneous cells at each timepoint.  This is especially important to address given the authors' exclusive focus on activation.  One expectation if these are indeed distinct cell identities is that SEs would be activated and deactivated at each timepoint.  Do any SEs become decommissioned?  Do SEs become partially activated in a TH manner only to not reach final SE status in the terminal state?

2) The major innovation in this manuscript's analysis is in a framework for analyzing SE constituents across all SEs, which are heterogeneous in their numbers of constituents.  The authors break SEs into four "interpolated" enhancer constituents.  The most signal-rich and least signal-rich constituents are represented by actual data, but the signal of intermediate signal-rich constituents are interpolated geometrically.  This is a clever technique.  I am, however, having substantial difficulty interpreting heatmap figures (e.g. 1B, 4A) in these terms.  My main difficulty is created by observing multiple patterns: Con constituents are generally most signal-rich in the middle two columns for each cell type; TH constituents are generally most signal-rich in the right-most column for each cell type; DN constituents are generally most signal-rich in the left-most column for each cell type.  This phenomenon appears to be consistent across systems as well.  To put a fine point on it, what does each of the four columns represent with respect to the SE they're contained in?  They do not appear to be ordered by their dominant signal, as otherwise the right-most column would have the same general character in all three SE groups.  These figures would benefit strongly from a better labeled X axis.  

3) One of the initial attempts at understanding the functions of SEs and the heterogeneous activities of their constituents was performed by the original SE-identifying group (PMID: 25801169).  The major takeaway is that SE constituents are generally bound by master transcription factors and specific subsets of constituents are bound by the terminal transcription factors of signaling pathways.  It has also been observed that, during differentiation, master TFs tend to recruit signaling TFs to their sites (PMID: 22036566).  Although potentially following a slightly different chronology, the authors' data show signs that this model is applicable to SE development.  Indeed, nuclear factors, STATs, TCFs, NFkB, etc. whose motifs are observed as enriched in differentiation-specific SEs in 5B, SF7 are such signaling TFs.  It would be useful to understand whether these signaling factors are specifically enriched at a special subset of constituents.  This may be especially interesting because of the stringent 2 RPKM cutoff for assigning TF motif hits to a given TF, as many signaling TFs can recognize highly similar sequences.  

4) The data structure developed here is of great utility in asking about SE building during differentiation.  There are multiple questions that, if asked with this same structure, would strengthen the impact of this paper on the SE literature.  
---Figure 1C shows an SE that grows from the leftmost constituent to the rightmost.  Another model for SE growth, partially supported by 5D is that the inside constituents precede the outer ones.  What is the fraction of SEs that grow from the inside vs the outside?
---These data could be better integrated with the looping data to answer which event happens first: enhancer signal at a given constituent or its interaction with other enhancers?

5) I have some basic technical concerns about the computational aspects of the approach:
5a. In my own experience, I have found that MACS2 broad-peaks mode has a substantial false positive rate, where a false positive refers to passing an eye test while viewing coverage tracks.  I suspect the authors ran into this same issue, which is why they chose to remove peaks narrower than 300bp.  I can see several instances where peaks called by MACS2 are not credible based on local background (Figure 2E constituent #3, Figure 5C constituent #6, Figure 5D constituent #1, #4).  I recommend attempting these analyses with peaks called by MACS 1.4.  The peak-calling is much more stringent in my own experience and has been used by many of the SE-calling papers (Whyte et al. 2013, Hnisz et al. 2013, etc.) to identify H3K27ac constituents.
5b. Assigning enhancers to their likely target genes is an outstanding challenge in the field.  The authors apply a multi-step approach using both proximity and expression correlation to pick single targets of individual genes.  An approach identifying the single most proximal gene to an SE is supported by high-confidence looping in at least the vast majority of cases (83%, PMID: 25303531), so I am confident in the assignment to proximal genes.  I am less convinced that applying a correlation metric (enhancer signal vs. gene expression) is safely applicable here.  My main issue is in applying it to de novo enhancers.  One would expect the target genes of de novo enhancers to be largely unexpressed in the system until the final timepoint.  Therefore, the majority of the values in the correlation would be zero or almost zero, incorporating much noise into such a correlation.  I would not be too concerned about this assignment procedure if it weren't for the authors' attempt to argue that de novo enhancers are further away from their likely target genes.  


MINOR POINTS:
1) The authors provide helpful context for the special characteristics of SEs and in characterizing the subtypes of SEs in some figures, but do not carry the same technique throughout.  Specifically, I believe the background of typical enhancers shown in Figure 3 would provide helpful context for understanding all characterization analyses and not just the compartment analyses (e.g. the TAD boundary analysis (3B), the FIRE analyses (3C, D).  If this is not desirable, I would suggest a background shuffling approach would provide similar context.
2) Figure 1D is missing a comparison between Con and DN for completeness.
3) ACTN2 is called out as being associated with a DN SE, but it is not displayed as a track similar to MYH7 is in Figure 2E.  I also have trouble believing MYH7 is actually a DN SE, as it has signal at constituent #1 in D5, D7, and D15, while constituent #2 has appreciable signal only at D7 and D15; arguably, constituents #3 and #4 never have appreciable signal, which may reflect my major point 5a above.  
4) The applications of the temporal approaches to other systems (murine cardiomyocytes, murine hematopoiesis, and transdifferentiation) would benefit from sample tracks showing genes and SEs and their change over differentiation
5) The "peak by time point matrix" mentioned in the text would be a useful supplemental table, which might contain the coordinates of the peak, the signal in each sample, and whether that enhancer forms part of an SE in given samples
6) 2E is really a de novo SE?

**Reviewer 3**

In this paper, Kai et al., analyzed the process of super enhancer formation during cell differentiation and reprogramming. Using time-course H3K27Ac ChIP-seq data, they showed that super enhancers can be divided into three different groups, conserved, temporally hierarchical and de novo. Analysis of RNA-seq data showed that temporal changes of super enhancer activity correlate with the level of target gene expression. Specifically, de novo super enhancers are suggested to regulate cell-type specific genes via long-range interaction. On the other hand, conserved and temporally hierarchical super enhancers are frequently located around TAD boundaries, suggesting that each group of super enhancers has distinct characteristics. Genome editing analysis of endogenous loci revealed that both early and late enhancers are required for the activity of temporally hierarchical super enhancers. I found that the results of the study are potentially interesting. However, to warrant publication in Genome Biology, the current form of manuscript should be extensively revised by addressing number of issues.

Major point
1.     First of all, authors abuse abbreviations in the text and figures, which makes it hard for readers to understand the paper. They should revise the manuscript to minimize the use of abbreviations.
2.     Throughout this study, definition of super enhancers is solely dependent on H3K27Ac, which was recently shown to be dispensable for enhancer activity in mouse ES cells (Zhang et al., Genome Biol 2020). To avoid potential mis-annotation of super enhancers in this study, authors should also take account the profile of H3K4me1 and other key regulatory factors such as Mediator, Brd4, CBP/P300 and cohesin. They are all shown to be enriched for super enhancers (Whyte et al., Cell 2013; Hnisz et al., Cell 2013).
3.     In all figures except Fig3B, authors only focus on super enhancers, and thus it is not clear if the finding and conclusion are unique features of super enhancers or conserved for canonical enhancers as well. All data should be compared with the analysis of canonical enhancers.
4.     In Fig1B, authors show temporal changes of the level of H3K27Ac. To address if these dynamic transitions are functionally important, authors should also examine the binding profile of other regulatory proteins such as Mediator, Brd4 and CBP/P300.
5.     In Fig1B and other places, authors show only three classes of super enhancers. I'm wondering if there are any enhancers whose activities are initially high and progressively lost during differentiation. These enhancers might be important for the maintenance of pluripotency in stem cells.
6.     In page 6, authors wrote that "each SE was mapped to one target gene based on genomic distance as well as correlation between H3K27ac signal intensity and gene expression". In the case of Fig1C, which one particular gene is assigned as a target of defined super enhancer? In the left panel, one might consider that four different enhancers are regulating individual HIST2H genes. Is it reasonable to assume that one super enhancer is regulating only one gene in the HIST2H cluster?
7.     In FigS2B and other parts of the figure, authors compare the distance between super enhancers and target genes. I speculate that this analysis is sensitive to the definition of enhancer-target gene pairs. Does author's conclusion hold true even when they use different methods for enhancer-gene assignment?
8.     In Fig2B, what does FC stand for? It is not defined in the main text or legend. Also, in Fig2C, what does the number in Y-axis mean? This kind of insufficient description can be found in many places. For example, in FigS3D, labeling of Y-axis in the color bar is missing. In Fig3D, it is not explained which color corresponds to which cell types. In Fig5E and F, it is not explained what kind of experiment did the authors perform to quantify the expression level. In addition, "EnhA" and "EnhB" are not defined in Fig5E. In the current form of the manuscript, readers will have difficulties in understanding the data. Authors should extensively revise text, legends and figures to clarify these and other issues.
9.     In Fig2D, authors show the expression profiles of ATP11A and LDB3. Doesn't the level of their neighboring genes such as ATP11AUN and OPN4 change over time? If they exhibit similar patterns, then is it reasonable to assign super enhancer shown in Fig1C to only one particular target gene? I also noticed that authors examined the expression of HITS genes as a group instead of analyzing individual gene in the cluster. This appears to be inconsistent with enhancer-target gene assignment approach authors employed. By the way, gene name should be written in italic.
10.     Also, in Fig2D, these trends can be seen in genome-wide?
11.     In the main text, authors discuss MYH7 and ACTN2 by referring Fig2E. However, only MYH7 locus is shown in the figure, which is confusing. More critically, based on the result shown in FigS2C, authors mention that there was significant increase in the expression of MYH7 after differentiation. However, the data of Day 15 replicate 2 appears not to support this conclusion. In addition, H3K27Ac profile and expression data are separately shown in different figures, and information of H3K27Ac is missing for ACTN2, MYH6, TNNI1 and GATA4. Both time-course H3K27Ac profiles and expression data should be shown in the same figure side-by-side for all analyzed genes.
12.     In Fig3B, why the minimum value of Y-axis is 0.5, but not 0?
13.     Do genes regulated by enhancers with higher FIRE score tend to exhibit higher expression level?
14.     In Fig5C, early enhancer is located in the intronic region of the gene. Did the authors confirm if deletion affects splicing of Aff1 transcripts or not? It might be possible that decreased level of Aff1 in Fig5E is mainly attributed to destabilization of aberrantly processed mRNA rather than enhancer deletion.
15.     In Fig5D, tested enhancers appear to be more proximal to Dnajb12 or Micu1, but result indicates that they specifically regulate distal Ddit4 gene. Is this experimentally verified enhancer-gene pair consistent with computational enhancer-gene pair prediction used in Fig2 and elsewhere?
16.     Both in Fig5C and D, does deletion of one enhancer affect the level of H3K27Ac and chromatin accessibility at surrounding enhancer regions?
17.     Y-axis of H3K27Ac and ATAC-seq data in Fig5C and D should be kept constant across cells.
18.     In FigS6C, authors analyzed cells treated with Yamanaka-factors. In general, differentiation and reprograming are developmentally opposite processes. However, authors only focus on super enhancers that progressively obtain H3K27Ac signal during these processes. I speculate that enhancers that become active during differentiation are silenced after reprogramming. Authors should address this point.

Minor point
1.     In Fig1A, schematics of mESC and iPSC are drawn. But they are not analyzed in the Fig1. This is confusing for readers.
2.     Based on the result in FigS2A, authors state that super enhancers they found are mostly cell type specific. Are these enhancers actually quiescent in other cell types?
3.     In page 10, authors discuss relative portion of each type of super enhancers in mouse and humans, and propose that their temporal patterns are evolutionary conserved. Do repertories of their loci and target genes are also conserved across species?
4.     In Fig3, authors use FIRE score for their analysis without explaining method details in the main text. FIRE is not a very common term in the field. Authors should provide sufficient explanation in the main text to make it understandable for general readers.
5.     In Fig6A, it looks as if ES cells are reprogrammed from iPS cells, which I think not correct.

**Reviewer 4**

Using publicly available ChIP-seq data on H3K27ac during differentiation from human embryonic stem cells to primitive cardiomyocytes, Kai et al. showed that the SEs emerge through three distinct patterns; conserved (Con), temporally hierarchical (TH), and de novo (DN). Con SEs show H3K27ac peaks throughout differentiation, TH SEs show gradual establishment of peaks that constitute the entire SE over time, and DN SEs show high H3K27ac peaks only at the last stage. DN SEs mainly regulate cardiomyocyte-associated genes, whereas Con and TH SEs regulate genes with more general functions. Also, the DN SE-associated genes exhibit more drastic change in transcriptional activity during differentiation, compared to more stable expressions from Con and TH SE-associated genes. 3D chromatin organizations also differ among the three SE types, such that higher fraction of DN SEs are located in 'B to A' compartment, and Con and TH SEs are enriched at TAD boundaries. The three distinct patterns of SEs are observed in other systems as well, including mouse cardiomyocytes, mouse hematopoietic differentiation, and TF-directed reprogramming process. Lastly, the authors showed that both early- and late-emerged enhancers in TH SEs are indispensable for target gene expression. Taken together, they demonstrate the heterogeneity of the SE population and distinct functional roles of different SEs in gene regulation.  

Overall, the authors ask an interesting question of how super enhancers (SE) are established over time during cell differentiation, and the results they report are potentially significant. However, some figures are not easy to follow, and biological interpretations on their observations are missing. This is especially so when the authors analyze how distinct SE types show different 3D chromatin organizations. For example, what does it mean to have high enrichment of TH and Con SEs in TAD boundary? Does establishment of SEs lead to more chromatin interactions, or does frequent chromatin interactions facilitate SE establishment? I believe that clarifications on some of the figures and texts can improve the significance of this paper. Below are specific comments I have:

1)     Figure 1B: in the methods, it says that each column represents 0th, 33.3th, 67th, and 100th percentile of H3K27ac signal. Then why is the normalized H3K27ac intensity is not the highest in the 4th column for Con SEs and DN SEs? I see highest peak in 2nd/3rd columns on Con SEs, and in 1st column in DN SEs
2)     Figure 1C shows clear distinctions among Con, TH, and DN SEs. But the gradual establishment of SEs in TH SEs are not as obvious in Fig. 1B. Rather than seeing gradual increase in intensity through different columns or differentiation process, I only see the strongest signal in the 4th column. Please clarify.
3)     The authors claim that the H3K27ac signal intensity among three SE types are comparable (as shown in Figure 1D). But, again based on Fig. 1B, the signal looks much higher in 1B. There seems to be some discrepancy, unless I misunderstand something.
4)     In Figure 2B and C, the authors showed fold changes between day 0 and day 15 and claim that the Con and TH SEs' levels are more or less maintained during differentiation while DN SEs show increase. If we compare day 0 and day 15, this should be the case, given the late establishment of DN SEs. Also, in Fig. 2D, some genes in Con and TH SE categories seem to show increase in intensity during reprograming. I'd be interested in seeing a trend of average change in gene expression from day 0 to day 15 (instead of comparing day 0 and 15 only) to better characterize the change in gene expression dynamics in different SE classes.
5)     Can authors comment on their interpretations of how different SE classes show different chromatin organization? What does it mean to have high enrichment of TH and Con SEs in TAD boundary? Do SEs cause more chromatin interactions, or frequent chromatin interactions lead to distinct SE establishment?
6)     The authors show that removal of early or late enhancers affect gene expression in differentiated cells (I assume this is at the final stage?). Is gene expression in early stages affected upon deletion of the late enhancer? As the authors claim that gene expression is maintained constant during differentiation in TH SE-associated genes, I am wondering whether late enhancers (even if not fully established) are also important for early stages, or whether early and late enhancer's contribution to gene expression changes over time. Measurement of gene expression from cells in different stages will answer this question (the cells where early or late enhancers were removed).

**Authors’ response to reviewers**

We would like to thank the reviewers for their constructive and helpful comments. We have addressed all the issues raised by the reviewers and revised the text and figures accordingly. Please find below our point-by-point responses in blue.

To distinguish the figures in this letter from those in the manuscript, we labeled figures in this letter by “Figure R#”. Figures in the manuscript were referred as “Figure #” or “Supplementary Figure #”.

**Response to Reviewer #1**

The manuscript "Mapping the Evolving Landscape of Super-Enhancers During Cell Differentiation" by Yan Kai and colleagues focuses on the dynamic restructuring of super-enhancers (SEs) in differentiating cardiomyocytes. The authors propose clustering SEs into three groups based on their presence during development - conserved, temporarily hierarchical (TH), and de novo. They demonstrate that the acquisition of SEs during development results in elevated expression of their target genes. They also demonstrate that all three classes of SEs are associated with the open chromatin. The observed difference between the three classes of SEs is in the GO annotation of their target genes (with the de novo class being strongly associated with cardiomyocyte-specific gene activity), difference in bound transcription factors (TFs), distance to the transcription start site of their target gene, and local chromatin interactions. The experimental validation of a few enhancers within two SEs confirms the important contribution of individual SE enhancer modules to SE activity. Overall, it’s an interesting study focusing on a dynamically-active sub-class of SEs.

We thank the reviewer for the summary, positive comments, and appreciation of our work.

Major concerns:

1.    I’m surprised the authors haven’t been able to identify a sub-class of SEs that demonstrates a gradual loss of SEs during differentiation, a trend opposite to the TH SEs. Given the well-known activity of SEs during early development, I’d expect this class to exist. If this sub-class does exist, I find it essential to report and contrast it to the TH SE sub-class in order to present a full picture of the dynamic SE landscape changes during differentiation.

In the original manuscript, we have focused on enhancers that are active at the last stage of the development course. By definition, this excludes any enhancer that is decommissioned during development. To recover such subclasses, we applied our analysis procedure to the time-reversed SE landscape changes. Specifically, we identified SEs in hESCs and tracked how their constituent enhancers are decommissioned during the differentiation. Of note, the decommissioned SEs can also be classified into three groups that are analogous to conserved (Con), temporally hierarchical (TH), or de novo (DN) (see Figure R1A and B below). Furthermore, genes associated with the three types of SEs (Figure R1C) display a concordant pattern with the activity dynamics of SEs. As a result, DN SE-associated genes undergo the most dramatic changes of transcription activities, whereas Con and TH SEs-associated genes remain relatively stably expressed throughout differentiation (Figure R1D). Genes associated with the three types of SEs demonstrate a similar hierarchy in expression specificity, where DN SEs-associated genes are most specifically expressed in hESCs (Figure R1E). Moreover, GO enrichment analysis shows that the DN SEs are enriched in biological functions related to multi-organism cellular process which is specific to hESCs, while Con and TH SEs are enriched in general functions. In consistent, the analysis on SE decommission in erythroid differentiation (Figure R2) reveals the same patterns. Altogether, the pattern of SE decommission during differentiation is consistent with what we observed in the SE activation process. All these new results have been added to Figure 5 and Sup Figure 8 in the manuscript.

*Figure R1. Analysis of SE decommission in human cardiomyocytes differentiation reveals similar patterns as we observed in the SE activation process. (A) Heatmap showing the three sub-classes of SEs defined by progressing landscape during differentiation. (B) Dynamics of average SE activity during differentiation. For each SE, its signals during differentiation were represented by the signals relative to the maximum H3K27ac intensity across the five differentiation stages. (C) Dynamics of genes associated with the three types of SEs. For each gene, its expressions during differentiation were represented by the expression values relative to the maximum expression across the five stages. (D-E) Violin plot showing the fold changes (Day15/Day00) (D) and specificity (E) of genes associated with the three types of SEs. (F) GO analysis showing enriched functions for the three types of SEs.*

*Figure R2. Analysis of SE decommission during the differentiation of erythroid cells reveals similar patterns as we observed in the SE activation process. (A) Heatmap showing the three sub-classes of SEs defined by progressing landscape during differentiation. (B) Dynamics of average SE activity during differentiation. For each SE, its signals during differentiation were represented by the signals relative to the maximum H3K27ac intensity across the differentiation stages. (C) Dynamics of genes associated with the three types of SEs. For each gene, its expressions during differentiation were represented by the expression values relative to the maximum expression across the seven stages. (D-E) Violin plot showing the fold changes (EryB/LT-HSC) (D) and specificity (E) of genes associated with the three types of SEs. (F) GO analysis showing the enriched functions for the three types of SEs.*

2.   Assigning a SE to a single gene and doing so without chromatin contact data is a questionable assumption. SEs are long genomic regions that often span multiple gene loci. It is not uncommon for SEs to regulate multiple genes in their proximity. It might eliminate some concerns with this arbitrary selection of SE target genes, if the authors could demonstrate that an alternative assignment of SEs to all expressed genes in their proximity, for example, wouldn’t change the reported results. Using chromatin contact data for establishing the set of target genes for an SE would be an alternative option.

We thank the reviewer for this critical point. Correct assignment of genes to SEs is an important step to study the biological function of the three types of SEs. To demonstrate that the reported results on the differences of the three types of SEs are robust, we have applied two alternative ways of SE-gene assignment: (1) Nearest genes in each direction, i.e. each SE was assigned to the nearest gene in two directions respectively (Figure R3); (2) All expressed genes in proximity, i.e. each SE was assigned to the expressed genes (RPKM > 2) within 100kb of the SE (Figure R4). Both methods reveal consistent patterns with the reported results, in terms of the gene expression fold changes and differences in expression specificity. As shown in Figures R3 and R4, the main results regarding the expression patterns of genes associated with the three types of SEs were not changed, suggesting the robustness of the reported results on the differences of the three types of SEs.

In addition, we agree with the reviewer that it is common for SEs to regulate multiple genes in their proximity. Therefore, we have modified the method for SE-gene assignment in the following way: all expressed genes (RPKM > 2) were considered as the target genes of a SE if (1) they are within 100 kb of the SE (2) their gene expressions correlate well (Pearson correlation >= 0.75) with the H3K27ac signals of the SE during differentiation. In this way, multiple genes can be assigned to a SE. All relevant results in the revised manuscript were generated from this method.

*Figure R3. Expression level changes of genes associated with the three types of SEs in the human cardiomyocytes differentiation process. Two nearest genes in both directions of a SE are assigned as the target genes. (A) Dynamics of genes associated with the three types of SEs. (B-C) Violin plots showing the fold changes of genes associated with the three types of SEs (B) and the specificity (C) to the terminal stage.*

*Figure R4. Expression level changes of genes associated with the three types of SEs. All expressed genes (RPKM>=2) within 100kb of an SE were assigned as targeted genes. (A) Dynamics of genes associated with the three types of SEs. (B-C) Violin plots showing the fold changes of genes associated with the three types of SEs (B) and the specificity (C) to the terminal stage.*

3.   The role of SEs in A/B compartment switching during development is an interesting hypothesis, which finds no validation by this study. All three sub-classes of SEs are strongly enriched in the "Stable A" set and the differences between the three classes are not significant. Therefore, I’d strongly recommend either moving this analysis to Supplemental Materials or reevaluating the reported conclusions, as they are currently rather overenthusiastic and misleading.

We thank the reviewer for this comment. We re-evaluated the conclusions and found the same conclusion was reached. Therefore, we followed the reviewer’s suggestion and moved this analysis to Supplementary Figure 3 in the revised manuscript.

4.    One of my main concerns is the lack of focus on the TH SEs dataset, which is the key novelty of the presented study. The effects reported for the two other ("stable") datasets are rather expected and reiterate what has been reported on SEs in the past (elevated gene expression, open chromatin state, multiple chromatin contacts, presence of key activator binding sites, etc, etc). The TH sub-class is novel and represents a dynamic change in SE landscape during development. In the current version of the manuscript, it is characterized as an "intermediate" sub-class effectively "averaging" between the conserved and de novo sub-classes of SEs. There is little if any investigation of the role of TH SEs during development and the essentiality of this sub-class of SEs for differentiation.

This is a very important point. To investigate the roles of TH SEs, we performed GO analyses and showed that TH SEs are enriched in terms of relatively general biological functions, such as “post-transcriptional regulation of gene regulation” and “regulation of cellular amide metabolic progress” (Figure 2a in the manuscript). However, to study the roles of TH SEs during development and their essentiality for differentiation, a major approach is to perturb the SEs through genome editing techniques. Because SEs usually span over tens of kilobases and might contain genes, it is not practical to delete the whole SE region for such investigations. Furthermore, to gain systematic views of the TH SEs’ roles during differentiation, it is necessary to perform genome editing for many SEs, which is extremely challenging and beyond the scope of our study. Instead of investing in the roles of the TH SEs, we are exploring the roles of TH SEs’ individual elements that come into commission in different stages during differentiation for regulating target gene expression. The details of the experimental investigation can be seen in next point.

5.   Finally, I find the experimental component of the study interesting but rather irrelevant to the presented study itself. While the manuscript focuses on the three sub-classes of SEs, the experimental work seeks to distinguish between dynamically-active individual enhancers within a SE. The experiments focus on early, intermediate, and late enhancers within two selected TH SEs and were performed in a mature cell line. The observed result of the importance of individual enhancers for SE activity aligns well with what had been reported in the past. However, this addresses neither the dynamic activity of TH SEs during development nor even the dynamic activity of individual TH SE enhancers during development. Would the KO of two individual enhancers have the same effect during development when one of the enhancers is supposedly inactive? Would that imply that TH SEs gradually gain strength during development or there is an activation threshold in the number of individual enhancers, which acts as an on/off SE switch?

We thank the reviewer for the thoughtful comment. As we discussed in point #4, our experimental investigation focused on the roles of individual enhancers that become active at different stages. Our investigation in MEL cells (a mature erythroid cell-line) suggests that both the early and late enhancers are indispensable for maintaining the expression of target genes. Of note, our investigation is different from past studies because we selected the enhancers that come into commission at different stages, while past studies did not take the temporal differences into consideration.

We agree with the reviewer it is important to examine the effect of enhancer perturbation in the context of cell differentiation rather than a static system. To address this point, we carried out additional experiments to characterize the function of enhancer constituents within TH SEs. We did this for different developmental stages through CRISPR-cas9 mediated genetic perturbation in two well-established systems: Erythroid-Myeloid-Lymphoid (EML) cells and G1E erythroid cells. The results suggest that early enhancers play an important role at both the undifferentiated and differentiated stages, whereas late enhances play an important role at the differentiated stage. The details are described below.

EML cells reside in the murine bone marrow cells, and serve as multipotent hematopoietic precursor cells which can differentiate into erythroid, myeloid or lymphoid cells (PMID: 16352715). As such, it is an ideal system to test the role of enhancers at the undifferentiated stage. To this end, we selected the same loci (Ddit4 super-enhancer constituents) for testing so that the results can be compared with our previous data obtained from mature erythroid MEL cells (old Figure 5). In brief, we used CRISPR-Cas9-mediated genome editing to delete the early and late enhancer at the Ddit4 super-enhancer locus in undifferentiated EML cells and quantified transcripts of surrounding genes by qPCR.

We found that deletion of early enhancer leads to a significant decrease of Ddit4 transcripts (new Figure 6e in the manuscript and Figure R5a below), while deletion of late enhancer has no effects on Ddit4 gene expression (Figure R5a). Transcripts of Dnajb12 and Micu1 remain unchanged upon deletion of either early enhancer or late enhancer (Figure R5a). In contrast, in the differentiated erythroid cells represented by MEL cells, both early enhancer and late enhancer are required for Ddit4 expression (Figure 6f in the manuscript).

As an additional validation, we applied the same approach to characterize the function of Ddit4 SE constituents in murine G1E cells, resembling proerythroblasts, which is an immortalized GATA-1–null line commonly used as a model for immature erythroid cells (PMID: 15297311). Consistently, deletion of early enhancer, but not late enhancer, reduced Ddit4 transcripts in undifferentiated G1E cells (Figure R5b). Together with our previous results from MEL, these data indicate that late enhancers are dispensable at the early stage but required for transcriptional control at the differentiated stage, whereas the early enhancers are required for gene expression at both stages.

In addition, we also explored the possibility of comparing the dynamic response during EML differentiation. Consistent with our model, we found that deletion of the late enhancer caused increased differences of *Ddit4*’s expression comparing to the undifferentiated EML cells, whereas the deletion of early enhancer continues to have a profound effect on the target gene’s expression. However, our data is inconclusive because the difference is not statistically significant. A likely explanation is that the differentiation protocol we used is inefficient, as differentiated EML cells are able to express some beta-hemoglobin (*Hbb-b1*) but cannot express erythroid cell surface marker, such as Ter119. To avoid any confusion, we have decided not to discuss this aspect in our manuscript, although we agree it is important to reinvestigate in the future using other systems.

*Figure R5. Functional validation of the early and late enhancers within a TH SE. (A) Expression comparison in wild type (WT) and enhancer-knockout EML (undifferentiated hematopoietic progenitor cells). **, p < 0.01; n.s., not significant, Student’s t-test. (B) Expression comparison in wild type (WT) and enhancer-knockout G1E cells, resembling proerythroblasts. **, p < 0.01; n.s., not significant, Student’s t-test. (C) Expression comparison in wild type (WT) and enhancer-knockout in differentiated day 7 EML cells.*

Minor comments:

*    Line 175, why DN SEs show a lower enrichment in TAD boundaries as compared to Con and TH SEs but contain more frequently interacting regions?

We hypothesized that this is because TAD boundaries are usually enriched in chromatin interactions biased towards the interior domain (i.e. **in one direction**) but are depleted in interactions towards the other direction. The algorithm we used to identify frequently interacting regions measures the chromatin interactions **in both two directions**. Our results are consistent with the conclusions reported previously. For example, the genome-wide FIRE score was shown to have a dip around TAD boundaries (Figure 2A in PMID:27851967) and the identified FIREs were shown to be depleted in TAD boundaries (Figure 2E in PMID:27851967).

*    Line 279, the authors suggest a correlation between the order of elements and TF binding, but didn’t discuss why it is the ETS family and not other TF families is enriched in early enhancers? Is there a functional difference between early and late enhancers?

We hypothesized that early enhancers are enriched in ETS family factors because ETS factors are known to maintain general cellular functions (see PMID: 28275002), considering that early enhancers come into activity from early stages and are maintained at high levels throughout the differentiation.

*    Figure 5E, does the notation "EnhB" and "EnhA" implies "early" and "late" enhancers. Please clarify.

We apologize for missing this notation. “Early” and “late” notations have been added in revised Figure 6.

*    Figure 2B, the distribution shows a gradual increase in gene expression associated with the three sub-classes of SEs? Were there any cases of an opposite trend?

We thank the reviewer for raising this point. We added the result showing the dynamic expression during the whole differentiation process (Figure R6). Indeed, we saw a gradual increase in gene expression associated with all three types of SEs. The differences among the three types of SEs are that the DN class shows a rapid increase while the Con and TH classes show a moderate increase. This new analysis was added to main figures in the manuscript, such as Figure 2c and Figure 4c. The opposite trend was observed when we studied SE’s decommission process (Figure 5c). Please see details in Figure R1 and R2 in point #1.

*Figure R6. Expression dynamics for genes associated with the three types of SEs for the human cardiomyocytes differentiation (left) and mouse erythroid cells differentiation (right). For each gene, the expression values relative to its maximum during all differentiation stages were used for plotting.*

*    Figure 3D, the color-code legend is missing.

We have added the legend in the revised figure (Figure 3C in the revised manuscript).

*    There are multiple typos throughout the manuscript. The manuscript would definitely benefit from a careful proofreading.

We apologize for the typos. We have fixed all typos in our revised manuscript.

**Response to Reviewer #2**

The manuscript from Kai et al. uses a suite of computational approaches to uncover patterns of super-enhancer (SE) activation during differentiation.  The patterns they are most interested in describe whether SEs emerge as whole units at distinct stages or whether the constituent enhancers of SEs appear in a stepwise manner during a differentiation pathway.  To study these patterns, the authors use published high-throughput sequencing data from a range of differentiation systems.  The approaches applied to these data show that developmental SEs fall into both de novo and temporally hierarchical classes (as well as a third, stable class active in all differentiation stages).  The authors then classify individual enhancers within SEs into their patterns of activity and perform experiments to demonstrate that both constituent enhancers that appear in the middle of the differentiation and constituent enhancers that appear at the final stage matter for end-stage gene expression.

The existence of SEs and their roles in driving cell identity programs is largely well-established.  Indeed, the authors note several efforts at dissecting the relationship of constituent enhancers of SEs, but these have generally been performed at steady state in one cell identity.  The kinetics of SE activation remain less understood.  To that end, this manuscript makes an admirable first step toward understanding how differentiating cells "build" SEs.  The clever computational device used here, namely forcing SE constituents into a four-constituent array, makes biological and computational sense, and the authors use it to make important observations about SE biology.

I nevertheless have concerns about the method itself, largely around a reader’s ability to interpret the results from the figures presented.

We thank the reviewer for the comprehensive summary of our manuscript, and the positive comments of our work.

MAJOR POINTS:
1) I recognize that the data analyzed in this manuscript are in public repositories, but I wonder if the authors can briefly comment on whether each system has distinct biological stages with intermediate, meta-stable cell identities or whether each system is actually an artificial dissection of what is really a continuum of heterogeneous cells at each timepoint.  This is especially important to address given the authors’ exclusive focus on activation.  One expectation if these are indeed distinct cell identities is that SEs would be activated and deactivated at each timepoint.  Do any SEs become decommissioned?  Do SEs become partially activated in a TH manner only to not reach final SE status in the terminal state?

We thank the reviewer for raising this point. We have confirmed that all the systems have distinct biological stages, except the mouse reprogramming system in the study. Specifically, for mouse blood cells (PMID: 25103404) and mouse cardiomyocytes (PMID: 22981692), the cell populations at each stage were obtained by FACS using specific markers to each cell type. For the human cardiomyocyte differentiation system (PMID: 31427791), the engineered hESC cell line was differentiated into cardiomyocytes and their intermediate developmental stages utilizing a well-established Wnt-based cardiomyocyte differentiation protocol, and the differentiation efficiency was assessed at each developmental stage by flow cytometry using antibodies against markers specific to each stage. In the mouse cell reprogramming system (PMID: 28111071), the cells at the intermediate stages do not involve cell purification steps.

The reviewer’s comment regarding enhancer decommission is interesting. To analyze the temporal hierarchy of decommissioned enhancers, we performed similar analyses for SEs by reversing the time direction, focusing on those enhancers that are active at the pluripotent stage. Indeed, the decommissioned SEs can also be classified into three groups that are analogous to conserved (Con), temporally hierarchical (TH), or de novo (DN) (see Figure R1A and B). Furthermore, genes associated with the three types of SEs (Figure R1C) display a concordant pattern with the activity dynamics of SEs. As a result, DN SE-associated genes undergo the most dramatic changes of transcription activities, whereas Con and TH SEs-associated genes remain relatively stably expressed throughout differentiation (Figure R1D). Genes associated with the three types of SEs demonstrate a similar hierarchy in expression specificity, where DN SEs-associated genes are most specifically expressed in hESCs (Figure R1E). Moreover, GO enrichment analysis shows that the DN SEs are enriched in biological functions related to multi-organism cellular process which is specific to hESCs, while Con and TH SEs are enriched in general functions. In consistent, the analysis on SE decommission in erythroid differentiation (Figure R2) reveals the same patterns. Altogether, the pattern of SE decommission during differentiation is consistent with what we observed in the SE activation process. All these new results have been added to Figure 5 and Sup Figure 8 in the manuscript.

Because the time points are limited, it is difficult to reliably identify enhancers with transient activity at an intermediate time point. As such, we decided not to investigate the dynamic patterns of such transient SEs, although we agree with the reviewer it is a very interesting question.

2) The major innovation in this manuscript’s analysis is in a framework for analyzing SE constituents across all SEs, which are heterogeneous in their numbers of constituents.  The authors break SEs into four "interpolated" enhancer constituents.  The most signal-rich and least signal-rich constituents are represented by actual data, but the signal of intermediate signal-rich constituents are interpolated geometrically.  This is a clever technique.  I am, however, having substantial difficulty interpreting heatmap figures (e.g. 1B, 4A) in these terms.  My main difficulty is created by observing multiple patterns: Con constituents are generally most signal-rich in the middle two columns for each cell type; TH constituents are generally most signal-rich in the right-most column for each cell type; DN constituents are generally most signal-rich in the left-most column for each cell type.  This phenomenon appears to be consistent across systems as well.  To put a fine point on it, what does each of the four columns represent with respect to the SE they’re contained in?  They do not appear to be ordered by their dominant signal, as otherwise the right-most column would have the same general character in all three SE groups.  These figures would benefit strongly from a better labeled X axis.

We thank the reviewer for the helpful comment. The four representative enhancers at each SE locus were not ranked by their dominant signal (e.g. the mean signal during the differentiation stages), but by their variation level (i.e. Relative Standard Deviation (std/mean)). We chose to rank the enhancers by variation because it is desirable to make sure that constituent enhancers with similar dynamics (e.g. variable or stable) between different SEs are compared with each other in the clustering step. To make the figure easier to follow, we added this information to the *x-*axis and in the figure legends of Supplementary Figure 1b.

3) One of the initial attempts at understanding the functions of SEs and the heterogeneous activities of their constituents was performed by the original SE-identifying group (PMID: 25801169).  The major takeaway is that SE constituents are generally bound by master transcription factors and specific subsets of constituents are bound by the terminal transcription factors of signaling pathways.  It has also been observed that, during differentiation, master TFs tend to recruit signaling TFs to their sites (PMID: 22036566).  Although potentially following a slightly different chronology, the authors’ data show signs that this model is applicable to SE development.  Indeed, nuclear factors, STATs, TCFs, NFkB, etc. whose motifs are observed as enriched in differentiation-specific SEs in 5B, SF7 are such signaling TFs.  It would be useful to understand whether these signaling factors are specifically enriched at a special subset of constituents.  This may be especially interesting because of the stringent 2 RPKM cutoff for assigning TF motif hits to a given TF, as many signaling TFs can recognize highly similar sequences.

We thank the reviewer for this insightful comment. As the reviewer pointed out, from the motif enrichment results, we can see that those signaling factors, such as STAT factors in the human cardiomyocyte system, are enriched in the enhancers within DN SEs, suggesting that these signaling factors are enriched in late enhancers. To further investigate whether the signaling factors are specifically enriched at a subset of enhancer constituents, we used Binding Analysis for Regulation of Transcription (BART), a tool to infer functional transcription factors that bind to a set of genomic regions. BART leverages thousands of real ChIP-seq datasets from the public domain for regulator prediction, which does not require the 2 RPKM cutoff for motif enrichment. Using the erythroid system as an example, we found that BART analysis confirms the results from the motif enrichment and also reveals new insights (Figure R7 top panel). First, the master regulators for erythropoiesis, such as GATA1 and TAL1, were found more enriched in late enhancers than early enhancers or conserved enhancers, consistent with the motif enrichment result. Second, the architecture proteins CTCF and RAD21 also show an increasing enrichment in late enhancers, likely suggesting that late enhancers are more involved in regulation related to higher-order chromatin structure. Notably, this enrichment is repeatedly observed in other systems (Figure R7), suggesting the robustness of the finding. We added this new analysis to Supplementary Figure 10f.

*Figure R7. BART analysis revealing the enriched factors on different sets of enhancers.*

4) The data structure developed here is of great utility in asking about SE building during differentiation.  There are multiple questions that, if asked with this same structure, would strengthen the impact of this paper on the SE literature.
---Figure 1C shows an SE that grows from the leftmost constituent to the rightmost.  Another model for SE growth, partially supported by 5D is that the inside constituents precede the outer ones.  What is the fraction of SEs that grow from the inside vs the outside?
---These data could be better integrated with the looping data to answer which event happens first: enhancer signal at a given constituent or its interaction with other enhancers?

We thank the reviewer for the helpful suggestions. We investigated these two interesting problems below:

(1) To reveal the direction of SE growth, we measured the consistency between enhancers’ temporal order and their position within a TH SE. As illustrated in Figure R8a, a typical SE growing from the left to the right would have a high positive Spearman rank correlation between the temporal order of enhancers and their positions, and a SE growing from inside to outside would have a relatively low correlation. Specifically, we used Relative Standard Deviation (RSD, std/mean) for individual constituent enhancers to measure their temporal order, similar to the method we used in the SE classification (Figure 1b and Supplementary Figure 1b). An early enhancer would have low RSD and late enhancers would have high variation. The distribution of such correlations in human cardiomyocyte and mouse erythroid cells’ differentiation was plotted below (Figure R8b). To select the SEs with strong grow direction preference, we defined that a SE grows from outside if the absolute value of spearman rank correlation is higher than 0.8, and a SE grows from inside to outside if the absolute value of the correlation is lower than 0.2. As shown in Figure R8c, our data show that most of the SEs do not show a strong preference in growing direction, and there are more SEs growing from inside to outside than those growing from outside.

F*igure R8. Analysis of SE growth direction. (A) Illustrations of our approach to dissect the growing direction of SEs. (B) Histograms showing the distribution of spearman rank correlation between the temporal order of enhancers and their positions. The high absolute value of spearman rank correlation indicates a SE growing from outside, and the low absolute value of such correlation indicates a SE growing from inside. (C) Pie charts showing the number of SEs showing strong preferences in growing directions.*

(2) We agree with the reviewer that integration with the looping information would be interesting to reveal the temporal order of the two events: the activation of enhancers and the formation of loops. However, we found that such integration requires very high resolution of the looping data from Hi-C, and our attempts indicate that the quality of the loops called from the Hi-C data in this study is not good enough for this integration.

Specifically, we called loops from the Hi-C data using Fithic2 (v2.0.7) with a statistical cutoff (q = 1e-5) and obtained 71,541 loops from the Day 15 Hi-C data. Visual inspection of the loops suggests that the majority of the called loops are just delineating the strong interactions located around the TAD boundaries and the genomic coordinates of the loop anchors have high fluctuations, as shown in Figure R9. Further characterization of the loops suggests that only 4% of all the loops (2,877 out of 71,541) are connecting one H3K27ac peak to another. Furthermore, only 273 out of the 2,877 H3K27ac loops are involving a TH-SE constituent enhancer. These results suggest that the current loops are not good enough for further integration analysis to dissect the question on the temporal order of the two events. Therefore, we did not carry on with this analysis.

*Figure R9. An example showing that the called loops are largely involving TAD boundaries but not enhancers. The top panel shows the called loops in a contact map. Each yellow dot represents a loop. The bottom panel shows the called loops around the TAD boundaries and H3K27ac peaks. The called loops form a loop cluster largely represents the strong interaction between the TAD boundaries but not H3K27ac peaks.*

5) I have some basic technical concerns about the computational aspects of the approach:
5a. In my own experience, I have found that MACS2 broad-peaks mode has a substantial false positive rate, where a false positive refers to passing an eye test while viewing coverage tracks.  I suspect the authors ran into this same issue, which is why they chose to remove peaks narrower than 300bp.  I can see several instances where peaks called by MACS2 are not credible based on local background (Figure 2E constituent #3, Figure 5C constituent #6, Figure 5D constituent #1, #4).  I recommend attempting these analyses with peaks called by MACS 1.4.  The peak-calling is much more stringent in my own experience and has been used by many of the SE-calling papers (Whyte et al. 2013, Hnisz et al. 2013, etc.) to identify H3K27ac constituents.

We used MACS2 in our original analysis because it is the standard practice now. On the other hand, we agree with the reviewer its performance in broad-peak detection sometimes is not as good as the earlier MACS1.4 version. Following the reviewer’s suggestion, we repeated the analysis by replacing MACS2.0 with MACS1.4 for peak calling. Indeed, we found that peaks called by MACS1.4 are more stringent than MACS2. In the mouse erythroid system, MACS1.4 reduces the peak number from 33,209 (q=0.01, MACS2) to 15,539 (p=1e-5, MACS1.4). Moreover, as shown in the genome browser tracks below (Figure R10), some suspicious peaks pointed out by the reviewer (highlighted in yellow) were removed in the peak set called by MACS1.4. Similar results were observed in the human cardiomyocyte differentiation system. For example, the peak number was reduced from 45,019 (q=0.01, MACS2) to 37,077 (p=1e-5, MACS1.4). Therefore, we repeated all the analyses using the MACS1.4 peaks in this study and replaced previous figures. All major conclusions are consistent with those in the previous manuscript.

*Figure R10. Genome browser examples comparing the peaks identified by MACS2 and MACS1.4 in the mouse erythroid cells. Yellow bars highlight the suspicious peaks identified by MACS2 and not identified by MACS1.4.*

5b. Assigning enhancers to their likely target genes is an outstanding challenge in the field.  The authors apply a multi-step approach using both proximity and expression correlation to pick single targets of individual genes.  An approach identifying the single most proximal gene to an SE is supported by high-confidence looping in at least the vast majority of cases (83%, PMID: 25303531), so I am confident in the assignment to proximal genes.  I am less convinced that applying a correlation metric (enhancer signal vs. gene expression) is safely applicable here.  My main issue is in applying it to de novo enhancers.  One would expect the target genes of de novo enhancers to be largely unexpressed in the system until the final timepoint.  Therefore, the majority of the values in the correlation would be zero or almost zero, incorporating much noise into such a correlation.  I would not be too concerned about this assignment procedure if it weren’t for the authors’ attempt to argue that de novo enhancers are further away from their likely target genes.

We thank the reviewer for raising this critical point. Correct assignment of genes to SEs is an important step for studying the biological function of the three types of SEs. To demonstrate that the reported results on the differences of the three types of SEs are robust, we tried two alternative ways of SE-gene assignment: (1) Nearest genes in each direction, i.e. each SE was assigned to the nearest gene in two directions respectively (see details in Figure R3); (2) All expressed genes in proximity, i.e. each SE was assigned to the expressed genes (RPKM > 2) within 100kb of the SE (see details in Figure R4). Both methods reveal consistent patterns with the reported results, in terms of the gene expression fold change and differences in expression specificity. In addition, considering it is common for SEs to regulate multiple genes in their proximity, we modified the method for SE-gene assignment in the original manuscript in the following way: all expressed genes (RPKM > 2) were considered as the target genes of a SE if (1) they are within 100 kb of the SE (2) their gene expressions correlate well (Pearson correlation > 0.75) with the H3K27ac signals of the SE during differentiation. In this way, multiple genes can be assigned to a SE.

We also modified the analysis on that de novo SEs are further away from their likely target genes. Instead of directly comparing the genomic distance between SEs and their likely target genes, we now compare the gene density within a certain range of different types of SEs (Figure R11), which is not dependent on the SE-gene assignment. We found that DN SEs tend to locate in more gene-sparse regions than Con and TH SEs. We repeated this analysis in all the systems we interrogated and same results were obtained, suggesting the robustness of the conclusion. This analysis has been added to the figures in the manuscript (Figure 2f and Figure 4g and Supplementary Figures 4-8g)

*Figure R11. Comparison of gene density near the three types of SEs (human cardiomyocytes).*

MINOR POINTS:
1) The authors provide helpful context for the special characteristics of SEs and in characterizing the subtypes of SEs in some figures but do not carry the same technique throughout.  Specifically, I believe the background of typical enhancers shown in Figure 3 would provide helpful context for understanding all characterization analyses and not just the compartment analyses (e.g. the TAD boundary analysis (3B), the FIRE analyses (3C, D).  If this is not desirable, I would suggest a background shuffling approach would provide a similar context.

We thank the reviewer for this helpful suggestion. In the revised manuscript, we have added the analyses of typical enhancers as the background in all relevant figures, such as Figure 2c-e.

2) Figure 1D is missing a comparison between Con and DN for completeness.

We have revisited the comparison of SE length and intensity after using MACS1.4 peaks. It seems that the three types of SEs show an inconsistent relationship with the MACS2 results and across different systems we analyzed in the study. Therefore, we have removed these analyses in our revised manuscript.

3) ACTN2 is called out as being associated with a DN SE, but it is not displayed as a track similar to MYH7 is in Figure 2E.  I also have trouble believing MYH7 is actually a DN SE, as it has signal at constituent #1 in D5, D7, and D15, while constituent #2 has appreciable signal only at D7 and D15; arguably, constituents #3 and #4 never have appreciable signal, which may reflect my major point 5a above.

We have added the track showing the *ACTN2* SE and it is shown in Supplementary Figure 2e together with *MYH7* SE in Supplementary Figure 2d.

MYH7 was identified as a DN SE in our analysis. It was not identified as a TH SE because TH SEs were featured by a constitutively active element throughout the differentiation (i.e. from Day00 to Day15). We agree with the reviewer that the *MYH7* SE shows some degree of progressing nature during the differentiation, but when looking from an extended timeline (Day00 to Day15 instead of Day05 to Day15), *MYH7* SE’s temporal dynamic pattern is more similar to a DN one.

4) The applications of the temporal approaches to other systems (murine cardiomyocytes, murine hematopoiesis, and transdifferentiation) would benefit from sample tracks showing genes and SEs and their change over differentiation.

We thank the reviewer for the comment. We have added sample tracks for different systems in our revised manuscript (Supplementary Figure 9).

5) The "peak by time point matrix" mentioned in the text would be a useful supplemental table, which might contain the coordinates of the peak, the signal in each sample, and whether that enhancer forms part of an SE in given samples.

We followed the reviewer’s suggestion and added the “peak by time point matrix” for all systems analyzed in this study as an excel file in the Supplementary Table 4 in our revised manuscript.

6) 2E is really a de novo SE?

Please see details in the minor point (3) above.

**Response to Reviewer #3**

In this paper, Kai et al., analyzed the process of super enhancer formation during cell differentiation and reprogramming. Using time-course H3K27Ac ChIP-seq data, they showed that super enhancers can be divided into three different groups, conserved, temporally hierarchical and de novo. Analysis of RNA-seq data showed that temporal changes of super enhancer activity correlate with the level of target gene expression. Specifically, de novo super enhancers are suggested to regulate cell-type specific genes via long-range interaction. On the other hand, conserved and temporally hierarchical super enhancers are frequently located around TAD boundaries, suggesting that each group of super enhancers has distinct characteristics. Genome editing analysis of endogenous loci revealed that both early and late enhancers are required for the activity of temporally hierarchical super enhancers. I found that the results of the study are potentially interesting. However, to warrant publication in Genome Biology, the current form of manuscript should be extensively revised by addressing number of issues.

We thank the reviewer for the nice summary of the study and the appreciation of our work. We have extensively revised our manuscript below as the reviewer suggested.

Major point
1.    First of all, authors abuse abbreviations in the text and figures, which makes it hard for readers to understand the paper. They should revise the manuscript to minimize the use of abbreviations.

We thank the reviewer for pointing this out. To address this concern, we have carefully examined the manuscript and figures and removed many unnecessary abbreviations in our revised manuscript. In addition, we provided a list of abbreviations at the end of the manuscript and Supplementary Table 1 to facilitate the reading.

2.    Throughout this study, definition of super enhancers is solely dependent on H3K27Ac, which was recently shown to be dispensable for enhancer activity in mouse ES cells (Zhang et al., Genome Biol 2020). To avoid potential mis-annotation of super enhancers in this study, authors should also take account the profile of H3K4me1 and other key regulatory factors such as Mediator, Brd4, CBP/P300 and cohesin. They are all shown to be enriched for super enhancers (Whyte et al., Cell 2013; Hnisz et al., Cell 2013).

We used the ROSE algorithm developed by Dr. Richard Young’s lab to define super-enhancers. To our knowledge, this has been accepted by the community as the standard definition. Although this may seem over-simplistic and many other factors, including those mentioned by the reviewer, have been noted to play an important role in gene regulation, we are not aware of any standard pipeline that integrates such information. More importantly, unfortunately we do not have such data for most of the systems we have examined. To be generally applicable, we decided to use the standard ROSE algorithm despite its limitations.

3.    In all figures except Fig3B, authors only focus on super-enhancers, and thus it is not clear if the finding and conclusion are unique features of super enhancers or conserved for canonical enhancers as well. All data should be compared with the analysis of canonical enhancers.

We have added canonical enhancers to all the involved analyses in our revised manuscript as suggested, such as Figure 1c and Figure 2c-e. Our analyses have shown that the behavior of such canonical enhancers is distinct from super-enhancers.

4.    In Fig1B, authors show temporal changes of the level of H3K27Ac. To address if these dynamic transitions are functionally important, authors should also examine the binding profile of other regulatory proteins such as Mediator, Brd4 and CBP/P300.

As pointed out in our response to the reviewer’s comment #2, the binding profiles of these additional factors are not publicly available. Therefore, we are not able to examine the binding profiles of these factors although we agree they could provide additional insights.

5.    In Fig1B and other places, authors show only three classes of super enhancers. I’m wondering if there are any enhancers whose activities are initially high and progressively lost during differentiation. These enhancers might be important for the maintenance of pluripotency in stem cells.

We agree with the reviewer that how the SEs in pluripotent cells are lost during the differentiation is an essential and interesting problem. To analyze the temporal hierarchy of decommissioned enhancers, we performed similar analyses for SEs by reversing the time direction, focusing on those enhancers that are active at the pluripotent stage. Indeed, the decommissioned SEs can also be classified into three groups that are analogous to conserved (Con), temporally hierarchical (TH), or de novo (DN) (see Figure R1A and B below). Furthermore, genes associated with the three types of SEs (Figure R1C) display a concordant pattern with the activity dynamics of SEs. As a result, DN SE-associated genes undergo the most dramatic changes of transcription activities, whereas Con and TH SEs-associated genes remain relatively stably expressed throughout differentiation (Figure R1D). Genes associated with the three types of SEs demonstrate a similar hierarchy in expression specificity, where DN SEs-associated genes are most specifically expressed in hESCs (Figure R1E). Moreover, GO enrichment analysis shows that the DN SEs are enriched in biological functions related to multi-organism cellular process which is specific to hESCs, while Con and TH SEs are enriched in general functions. In consistent, the analysis on SE decommission in erythroid differentiation (Figure R2) reveals the same patterns. Altogether, the pattern of SE decommission during differentiation is consistent with what we observed in the SE activation process. All these new results have been added to Figure 5 and Sup Figure 8 in the manuscript.

6.    In page 6, authors wrote that "each SE was mapped to one target gene based on genomic distance as well as correlation between H3K27ac signal intensity and gene expression". In the case of Fig1C, which one particular gene is assigned as a target of defined super enhancer? In the left panel, one might consider that four different enhancers are regulating individual HIST2H genes. Is it reasonable to assume that one super enhancer is regulating only one gene in the HIST2H cluster?

We agree with the reviewer that it is not reasonable to assume one super-enhancer regulates only one gene, especially when considering that a super-enhancer usually acts as a regulatory hub and can regulate multiple genes. To address this problem, we have considered all the expressed genes in proximity (<100kb) of an SE as the potential targets, and then assigned genes whose expression profile exhibit a high correlation (Pearson correlation >=0.75) with the super-enhancer’s H3K27ac intensity profile throughout differentiation. By doing so, a super-enhancer can be assigned to multiple genes in their proximity, agreeing with the role of regulatory hubs of super-enhancers. We have updated this result in the revised manuscript.

7.    In FigS2B and other parts of the figure, authors compare the distance between super enhancers and target genes. I speculate that this analysis is sensitive to the definition of enhancer-target gene pairs. Does author’s conclusion hold true even when they use different methods for the enhancer-gene assignment?

The assignment of enhancer elements to target genes is an important yet unsolved issue. To demonstrate that the reported results on the differences of the three types of SEs are robust, we tried two alternative ways of SE-gene assignment: (1) Nearest genes in each direction, i.e. each SE was assigned to the nearest gene in two directions respectively (see details in Figure R3); (2) All expressed genes in proximity, i.e. each SE was assigned to the expressed genes (RPKM > 2) within 100kb of the SE (see details in Figure R4). Both methods reveal consistent patterns with the reported results, in terms of the gene expression fold change and differences in expression specificity, suggesting that the reported differences in the three types of SEs are robust.

8.    In Fig2B, what does FC stand for? It is not defined in the main text or legend. Also, in Fig2C, what does the number in Y-axis mean? This kind of insufficient description can be found in many places. For example, in FigS3D, labeling of Y-axis in the color bar is missing. In Fig3D, it is not explained which color corresponds to which cell types. In Fig5E and F, it is not explained what kind of experiment did the authors perform to quantify the expression level. In addition, "EnhA" and "EnhB" are not defined in Fig5E. In the current form of the manuscript, readers will have difficulties in understanding the data. Authors should extensively revise text, legends and figures to clarify these and other issues.

We apologize for the insufficient description in the figures and text. In the revised manuscript, the text, figures, and figure legends have been extensively improved.

Specifically,

(1) In Figure 2b (new Figure 2d), we replaced “log2(FC)” with “log2(Fold change)” in the *y-*axis.

(2) In Figure 2c (new Figure 2e), the *y-*axis (i.e. specificity) represents how the genes associated with the three types of SEs are specifically expressed in the terminal stages, comparing to all other earlier stages. For example, if there are two genes whose dynamic expression profiles during the five stages are [1, 5, 5, 5, 5] and [1, 1, 1, 1, 5] respectively, we consider that the latter one is more specific to the terminal stage although they have the same fold changes.

(3) We added the labeling of the color bar (-log10(P-value)) in Figure S3D (new Supplementary Figure 4f) and other similar figures.

(4) We added the annotation of cell types in Figure 3d (new Figure 3c).

(5) For Figure 5e and f (new Figure 6e-f), we added the type of experiments in the text, and replaced the “EnhA/B” annotation with “KO Early” or “KO Late” in the figure.

9.    In Fig2D, authors show the expression profiles of ATP11A and LDB3. Doesn’t the level of their neighboring genes such as ATP11AUN and OPN4 change over time? If they exhibit similar patterns, then is it reasonable to assign super enhancer shown in Fig1C to only one particular target gene? I also noticed that authors examined the expression of HITS genes as a group instead of analyzing individual gene in the cluster. This appears to be inconsistent with enhancer-target gene assignment approach authors employed. By the way, gene name should be written in italic.

The expression profile of *ATP11A* near the temporally hierarchical SE is [0.03, 0.08, 0.07, 0.10, 0.24] (as measured by the RPKM level), which was considered not expressed (expressed/not-expressed cutoff: 1 RPKM) throughout the differentiation course. In contrast, *ATP11A*’s expression profile is [9.2, 9.2, 18.6, 20.6, 22.8]. Therefore, *ATP11AUN* does not show a similar pattern as *ATP11A*. Similar observation happens to the *OPN4* gene, whose expression profile during the differentiation is [0, 0.01, 0.04, 0.10, 0.19] which was not expressed, while the *LDB3* is highly expressed ([0.2, 0.2, 36.6, 147.7, 197.6]).

As mentioned in point 7, we revised the assignment rules in the revised manuscript. Thus, a super-enhancer can be assigned to multiple genes, which agrees better with the regulatory hub’s role of super-enhancers.

10.    Also, in Fig2D, these trends can be seen in genome-wide?

We have added the genome-wide analysis of the expression dynamics for genes associated with the three types of super-enhancers (see Figure R6), and the genome-wide trend agrees well with the example shown.

11.    In the main text, authors discuss MYH7 and ACTN2 by referring Fig2E. However, only MYH7 locus is shown in the figure, which is confusing. More critically, based on the result shown in FigS2C, authors mention that there was significant increase in the expression of MYH7 after differentiation. However, the data of Day 15 replicate 2 appears not to support this conclusion. In addition, H3K27Ac profile and expression data are separately shown in different figures, and information of H3K27Ac is missing for ACTN2, MYH6, TNNI1 and GATA4. Both time-course H3K27Ac profiles and expression data should be shown in the same figure side-by-side for all analyzed genes.

We thank the reviewer for this point. In the revised manuscript, the *ACTN2* track is shown in Supplementary Figure 2e together with the *MYH7* track in Supplementary Figure 2d.

We double-checked the gene expression for *MYH7* in the Day15 replicates, and the discrepancy pointed out by the reviewer is likely due to the extremely high expression in Day15 replicate 1. Specifically, the expression values (in terms of RPKM values) for *MYH7* across all the samples are [0.48(Day00_rep1), 0.31(Day00_rep2), 0.53(Day02_Rep1), 0.36(Day02_Rep2), 7.56(Day05_Rep1), 1.81(Day05_Rep2), 22.29(Day07_Rep1), 32.48(Day07_Rep2), 359.80(Day15_Rep1), 92.41 (Day15_Rep2)]. We can see that there is a great increase for *MYH7* in Day15 Rep2 comparing to other time points, supporting our statement.

Moreover, we followed the reviewer’s suggestion to show the H3K27ac profile side by side with the gene expression profile. The new figures are shown in Supplementary Figure 2b-c.

12.    In Fig3B, why the minimum value of Y-axis is 0.5, but not 0?

The *y-*axis represents the fold enrichment of super-enhancers over TAD boundaries. We did not use 0 as the minimum value for aesthetics considerations.

13.    Do genes regulated by enhancers with higher FIRE scores tend to exhibit higher expression level?

Yes. To reveal this relationship, we divided the SE-associated genes into three groups of equal size by the SE’s FIRE score: low, medium, and high, and compared their expression levels. As shown in Figure R12 below, the genes associated with SEs that are characterized with higher FIRE scores tend to exhibit higher expression levels.

Besides, in the original study (PMID:27851967) which designed FIRE scores, the authors performed a comprehensive study on FIRE elements and gene expression and showed that genes regulated by enhancers with higher FIRE scores tend to exhibit higher expression level.

*Figure R12. Boxplot showing the gene expressions for three groups of SE-associated genes. *, p<0.05, **, p<0.01, Mann-Whitney U test.*

14. In Fig5C, early enhancer is located in the intronic region of the gene. Did the authors confirm if deletion affects splicing of Aff1 transcripts or not? It might be possible that decreased level of Aff1 in Fig5E is mainly attributed to destabilization of aberrantly processed mRNA rather than enhancer deletion.

The Aff1 results have been removed from the revised manuscript.

15.    In Fig5D, tested enhancers appear to be more proximal to Dnajb12 or Micu1, but result indicates that they specifically regulate distal Ddit4 gene. Is this experimentally verified enhancer-gene pair consistent with computational enhancer-gene pair prediction used in Fig2 and elsewhere?

Yes. According to the new approach for super-enhancer-gene assignment, the Dnajb12 SE was assigned to two genes: Dnajb12 and Ddit4, whose expression profiles show a Pearson correlation 0.97 and 0.82 with the super-enhancer’s H3K27ac intensity profile, respectively.

16.    Both in Fig5C and D, does deletion of one enhancer affect the level of H3K27Ac and chromatin accessibility at surrounding enhancer regions?

We agree with the reviewer this is a very interesting question. The main goal of this study is to characterize the overall differences among SEs in terms of developmental control, rather than dependency among different constitutive elements. While this is certainly an interesting question, it is beyond the scope of the current study. Therefore, we have decided to address this question in the future.

17.    Y-axis of H3K27Ac and ATAC-seq data in Fig5C and D should be kept constant across cells.

We have kept the tracks from different cells with constant scales in the y-axis in the revised figures.

18.    In FigS6C, authors analyzed cells treated with Yamanaka-factors. In general, differentiation and reprograming are developmentally opposite processes. However, authors only focus on super enhancers that progressively obtain H3K27Ac signal during these processes. I speculate that enhancers that become active during differentiation are silenced after reprogramming. Authors should address this point.

During the differentiation process, there are SEs activated and deactivated at each time point. Considering the complexity of enhancer dynamics during the whole differentiation course, in the current manuscript, we did not discuss the intermediate SEs specific to each time point but focus on the SE activation process. However, we agree with the reviewer that SE decommission is also an essential and interesting question.

To this end, we applied our analysis procedure to the time-reversed SE landscape changes. Specifically, we identified SEs in hESCs and tracked how their constituent enhancers are decommissioned during the differentiation. Of note, the decommissioned SEs can also be classified into three groups that are analogous to conserved (Con), temporally hierarchical (TH), or de novo (DN) (see Figure R1A and B below). Furthermore, genes associated with the three types of SEs (Figure R1C) display a concordant pattern with the activity dynamics of SEs. As a result, DN SE-associated genes undergo the most dramatic changes of transcription activities, whereas Con and TH SEs-associated genes remain relatively stably expressed throughout differentiation (Figure R1D). Genes associated with the three types of SEs demonstrate a similar hierarchy in expression specificity, where DN SEs-associated genes are most specifically expressed in hESCs (Figure R1E). Moreover, GO enrichment analysis shows that the DN SEs are enriched in biological functions related to multi-organism cellular process which is specific to hESCs, while Con and TH SEs are enriched in general functions. In consistent, the analysis on SE decommission in erythroid differentiation (Figure R2) reveals the same patterns. Altogether, the pattern of SE decommission during differentiation is consistent with what we observed in the SE activation process. All these new results have been added to Figure 5 and Sup Figure 8 in the manuscript.

Minor point
1.    In Fig1A, schematics of mESC and iPSC are drawn. But they are not analyzed in the Fig1. This is confusing for readers.

We thank the reviewer for the comment. Figure 1a was used as a summary of all interrogated lineages/systems that have been analyzed in the study. We believe it is helpful for readers to have a glance at all systems that are involved in the study.

2.    Based on the result in FigS2A, authors state that super enhancers they found are mostly cell type-specific. Are these enhancers actually quiescent in other cell types?

To answer this question, we compared the three types of SEs with the SEs identified in multiple cell types in ENCODE (Figure R13). We found that majority of the SEs (>=70%) are quiescent in other cell types, while a higher fraction of Con SEs is active in other cell types than the TH and DN SEs. We added this analysis to Figure 2b in the revised manuscript.

*Figure R13. Heatmap showing the overlap between the three types of SEs identified in human cardiomyocytes with the other 24 cell types. The SEs of the 24 cell types were downloaded from SEdb (*[*http://www.licpathway.net/sedb/index.php*](http://www.licpathway.net/sedb/index.php)*). Numbers in the heatmap stand for the percentage of SEs identified in human cardiomyocytes with each cell type.*

3.    In page 10, authors discuss relative portion of each type of super enhancers in mouse and humans, and propose that their temporal patterns are evolutionary conserved. Do repertories of their loci and target genes are also conserved across species?

To see whether repertories of the three types of SEs’ target genes are also conserved, we performed the overlapping analysis of the genes for each type of SEs between human and mouse cardiomyocytes, as shown in Figure R14 below. We did not compare the SEs’ loci between mouse and human because the SEs usually are too broad for lift-over analysis between mouse and human. We found that the degree of the overlapping in SE-target genes tends to increase from Con to DN SEs. Considering that the DN SEs are more enriched in cell-identity programs defining cardiomyocytes, this analysis may suggest that the genes associated with cell identity are more conserved between mouse and human than other programs.

*Figure R14. Overlapping analyses showing the degree of overlap for target genes of the three types of SEs. For background analysis, 600 genes in mouse and human were randomly selected and compared 1000 times, and the medium number of overlapped genes were reported. Jaccard index was calculated for all the comparisons as a measure of the degree of overlap.*

4.    In Fig3, authors use FIRE score for their analysis without explaining method details in the main text. FIRE is not a very common term in the field. Authors should provide sufficient explanation in the main text to make it understandable for general readers.

We thank the reviewer for raising this point. In the revised manuscript, an explanation of FIREs in the text and a brief description in the associated figure legends have been added to improve the readability.

5.    In Fig6A, it looks as if ES cells are reprogrammed from iPS cells, which I think not correct.

We thank the reviewer for pointing it out. The reviewer is correct that the ES cells were not reprogrammed from the iPS cells. We followed the strategy used by the source study of the datasets (PMID: 28111071) and used the ES cells’ data to represent the final stage.

**Response to Reviewer #4**

Using publicly available ChIP-seq data on H3K27ac during differentiation from human embryonic stem cells to primitive cardiomyocytes, Kai et al. showed that the SEs emerge through three distinct patterns; conserved (Con), temporally hierarchical (TH), and de novo (DN). Con SEs show H3K27ac peaks throughout differentiation, TH SEs show gradual establishment of peaks that constitute the entire SE over time, and DN SEs show high H3K27ac peaks only at the last stage. DN SEs mainly regulate cardiomyocyte-associated genes, whereas Con and TH SEs regulate genes with more general functions. Also, the DN SE-associated genes exhibit more drastic change in transcriptional activity during differentiation, compared to more stable expressions from Con and TH SE-associated genes. 3D chromatin organizations also differ among the three SE types, such that higher fraction of DN SEs are located in ‘B to A’ compartment, and Con and TH SEs are enriched at TAD boundaries. The three
distinct patterns of SEs are observed in other systems as well, including mouse cardiomyocytes, mouse hematopoietic differentiation, and TF-directed reprogramming process. Lastly, the authors showed that both early- and late-emerged enhancers in TH SEs are indispensable for target gene expression. Taken together, they demonstrate the heterogeneity of the SE population and distinct functional roles of different SEs in gene regulation.

We thank the reviewer for the comprehensive summary of our study.

Overall, the authors ask an interesting question of how super enhancers (SE) are established over time during cell differentiation, and the results they report are potentially significant. However, some figures are not easy to follow, and biological interpretations on their observations are missing. This is especially so when the authors analyze how distinct SE types show different 3D chromatin organizations. For example, what does it mean to have high enrichment of TH and Con SEs in TAD boundary? Does establishment of SEs lead to more chromatin interactions, or does frequent chromatin interactions facilitate SE establishment? I believe that clarifications on some of the figures and texts can improve the significance of this paper. Below are specific comments I have:

We thank the reviewer for the positive feedback and helpful critiques of our work.

1)    Figure 1B: in the methods, it says that each column represents 0th, 33.3th, 67th, and 100th percentile of H3K27ac signal. Then why is the normalized H3K27ac intensity is not the highest in the 4th column for Con SEs and DN SEs? I see highest peak in 2nd/3rd columns on Con SEs, and in 1st column in DN SEs.

The four representative enhancers at each SE locus were not ranked by their dominant signal (e.g. the mean signal during the differentiation stages), but by their variation level (i.e. Relative Standard Deviation (std/mean)). We chose to rank the enhancers by RSD because it is desirable to make sure that constituent enhancers with similar dynamics (e.g. variable or stable) between different SEs are compared with each other in the clustering step. To make the figure easier to follow, we added this information to the *x-*axis and in the figure legends of Supplementary Figure 1b.

2)    Figure 1C shows clear distinctions among Con, TH, and DN SEs. But the gradual establishment of SEs in TH SEs are not as obvious in Fig. 1B. Rather than seeing gradual increase in intensity through different columns or differentiation process, I only see the strongest signal in the 4th column. Please clarify.

To show the differences in a gradual increase in H3K27ac signal intensity between the three types of SEs, we plotted their average SE dynamics (relative to the maximum signal) below. The three types of SEs demonstrate significant differences in their dynamics, where DN SEs show low signals in early stages and have the most dramatic changes in later stages and TH SEs show less variation throughout differentiation.

*Figure R15. Dynamics of SE activity during human cardiomyocyte differentiation. For each SE, its signals during differentiation were represented by the signals relative to the maximum H3K27ac intensity across the five differentiation stages.*

3)    The authors claim that the H3K27ac signal intensity among three SE types are comparable (as shown in Figure 1D). But, again based on Fig. 1B, the signal looks much higher in 1B. There seems to be some discrepancy, unless I misunderstand something.

The od Figure 1D compares the ranking of SE intensity of the three types of SEs and shows that there are no significant differences in the ranking, while Figure 1B shows the signal intensity of the SEs. After revisiting these analyses using the SEs with new peak calling method (see point 5a from reviewer 2) and testing in different systems, we realized that the three types of SEs display different ranking in terms of SE intensity and SE length across systems (data not shown), suggesting that there are no inherent differences between the three types of SEs in SE intensity and length. Therefore, we removed old Figure 1D and 1E in the revised manuscript.

4)    In Figure 2B and C, the authors showed fold changes between day 0 and day 15 and claim that the Con and TH SEs’ levels are more or less maintained during differentiation while DN SEs show increase. If we compare day 0 and day 15, this should be the case, given the late establishment of DN SEs. Also, in Fig. 2D, some genes in Con and TH SE categories seem to show increase in intensity during reprogramming. I’d be interested in seeing a trend of average change in gene expression from day 0 to day 15 (instead of comparing day 0 and 15 only) to better characterize the change in gene expression dynamics in different SE classes.

We thank the reviewer for the helpful comment. In the revised manuscript, we have added the average gene expression dynamics for the differentiation process (Figure 2b and 4b, also see Figure R16 below for the gene expression dynamics for the human cardiomyocytes differentiation (left) and mouse erythroid cells differentiation (right)). Genes associated with each type of SEs show a certain level of increase, whereas DN SEs-associated genes demonstrate the most dramatic changes and Con SEs-associated genes show relatively stable changes.

*Figure R16. Average expression dynamics for genes associated with the three types of SEs during human cardiomyocyte differentiation (left) and mouse erythroid cells differentiation (right).*

5)    Can authors comment on their interpretations of how different SE classes show different chromatin organization? What does it mean to have high enrichment of TH and Con SEs in TAD boundary? Do SEs cause more chromatin interactions, or frequent chromatin interactions lead to distinct SE establishment?

Previous studies have shown that TAD boundaries are enriched in constitutively actively transcribed regions. We believe our results showing the enrichment of TH and Con SEs in TAD boundaries echoes this discovery, considering that Con and TH SEs show constant activity throughout the differentiation process. Whether SEs cause more chromatin interactions or gain of chromatin interactions facilitate SE establishment is an intriguing question. However, we found that this question requires very high resolution of the looping data from Hi-C, and our attempts indicate that the quality of the loops called from the Hi-C data in this study is not good enough for answering this question. Specifically, we called loops from the Hi-C data using Fithic2 (v2.0.7) with a statistical cutoff (q = 1e-5) and obtained 71,541 loops from the Day 15 Hi-C data. Visual inspection of the loops suggests that the majority of the called loops are just delineating the strong interactions located around the TAD boundaries and the genomic coordinates of the loop anchors have high fluctuations, as shown in Figure R9. Further characterization of the loops suggests that only 4% of all the loops (2,877 out of 71,541) are connecting one H3K27ac peak to another. Furthermore, only 273 out of the 2,877 H3K27ac loops are involving a TH-SE constituent enhancer. These results suggest that the current loops are not good enough for further integration analysis to dissect the question on the temporal order of the two events. Therefore, we did not carry on with this analysis.

A recent study (PMID: 32483172) explored this problem in mouse erythroid differentiation by coupling high-resolution Tiled-C data and other epigenomics data such as chromatin accessibility, and showed that the “enhancer-promoter interactions are not established prior to gene expression, but formed gradually during differentiation, concomitant with progressive upregulation of gene activity”. However, consider the resolution limit in the Hi-C data we have, we are unable to interrogate this problem.

6)    The authors show that removal of early or late enhancers affect gene expression in differentiated cells (I assume this is at the final stage?). Is gene expression in early stages affected upon deletion of the late enhancer? As the authors claim that gene expression is maintained constant during differentiation in TH SE-associated genes, I am wondering whether late enhancers (even if not fully established) are also important for early stages, or whether early and late enhancer’s contribution to gene expression changes over time. Measurement of gene expression from cells in different stages will answer this question (the cells where early or late enhancers were removed).

We agree with the reviewer that it is important to test the function of the early and late enhancers in different stages. To address this point, we carried out additional experiments to characterize the function of enhancer constituents within TH SEs in Erythroid-Myeloid-Lymphoid (EML) cells through CRISPR-cas9 mediated genetic perturbation.

EML cells reside in the murine bone marrow cells, and serve as multipotent hematopoietic precursor cells which can differentiate into erythroid, myeloid or lymphoid cells (PMID: 16352715). As such, it is an ideal system to test the role of TH SEs at the undifferentiated stage. To this end, we selected the same loci (*Dnajb12-Ddit4* super-enhancer constituents) for testing so that the results can be compared with our previous data obtained from mature erythroid MEL cells (old Fig. 5). In brief, we used CRISPR-Cas9-mediated genome editing to delete the early and late enhancer at the *Ddit4* super-enhancer locus in undifferentiated EML cells and quantified the transcription levels of surrounding genes by qPCR.

We found that deletion of early enhancer leads to a significant decrease of *Ddit4* transcripts (new Figure 6e in the manuscript), while deletion of late enhancer has no effects on *Ddit4* gene expression (new Figure 6e in the manuscript). Transcripts of *Dnajb12* and *Micu1* remain unchanged upon deletion of either early enhancer or late enhancer (new Figure 6e in the manuscript). In contrast, in the differentiated erythroid cells represented by MEL cells, both early enhancer and late enhancer are required for *Ddit4* expression (new Figure 6f in the manuscript).

**Second round of review**

**Reviewer 1**

The authors have addressed all my prior concerns and I would be happy to see this manuscript published.

Some minor points:
1. Figure 5G is not referred anywhere in the main text.
2. Figure 6E, the legend is not aligned.
3. In regard to the Figure 1B and the Figure 5A, the number of temporally hierarchical (191:178) and the number of de novo SE (563:581) are very similar for the emerged and decommissioned SE, which seems rather strange.

**Reviewer 2**

I commend the authors on addressing all of my concerns from the initial submission.  The manuscript is timely, creative, and important.

**Reviewer 4**

The revised manuscript addressed all my concerns and comments. I think the revision has significantly improved the manuscript, and is suitable for publication in Genome Biology.

I read Reviewer 3's comments and the authors' response, and I believe that the authors successfully addressed most of the points raised by Reviewer 3.

These include 1) analysis of canonical enhancers to show that superenhancers behave differently from canonical enhancers, 2) classification of superenhancers that are initially high and progressively lost during differentiation, 3) reassignment of the superenhancer target genes, 4) text changes to facilitate the reading, and 5) changes in figures for correct labeling and better visualization.

There are some points that the authors failed to address, especially regarding the use of ChIP-seq profiles for other transcription factors or co-factors including Mediator, Brd4, and CBP/P300, etc. The authors noted that they couldn’t do the analysis because these data are not publicly available. Given that they didn't generate their own data, I think the authors made a reasonable choice to use H3K27ac ChIP-seq results to define superenhancer regions, which is commonly done in the field.
